# Combining location-and-scale batch effect adjustment with data cleaning by latent factor adjustment

**DOI:** 10.1186/s12859-015-0870-z

**Published:** 2016-01-12

**Authors:** Roman Hornung, Anne-Laure Boulesteix, David Causeur

**Affiliations:** Department of Medical Informatics, Biometry and Epidemiology, University of Munich, Marchioninistr. 15, Munich, D-81377 Germany; Applied Mathematics Department, Agrocampus Ouest, 65 rue de St. Brieuc, Rennes, 35042 France

**Keywords:** Batch effects, High-dimensional data, Data preparation, Prediction, Latent factors

## Abstract

**Background:**

In the context of high-throughput molecular data analysis it is common that the observations included in a dataset form distinct groups; for example, measured at different times, under different conditions or even in different labs. These groups are generally denoted as batches. Systematic differences between these batches not attributable to the biological signal of interest are denoted as batch effects. If ignored when conducting analyses on the combined data, batch effects can lead to distortions in the results. In this paper we present FAbatch, a general, model-based method for correcting for such batch effects in the case of an analysis involving a binary target variable. It is a combination of two commonly used approaches: location-and-scale adjustment and data cleaning by adjustment for distortions due to latent factors. We compare FAbatch extensively to the most commonly applied competitors on the basis of several performance metrics. FAbatch can also be used in the context of prediction modelling to eliminate batch effects from new test data. This important application is illustrated using real and simulated data. We implemented FAbatch and various other functionalities in the R package bapred available online from CRAN.

**Results:**

FAbatch is seen to be competitive in many cases and above average in others. In our analyses, the only cases where it failed to adequately preserve the biological signal were when there were extremely outlying batches and when the batch effects were very weak compared to the biological signal.

**Conclusions:**

As seen in this paper batch effect structures found in real datasets are diverse. Current batch effect adjustment methods are often either too simplistic or make restrictive assumptions, which can be violated in real datasets. Due to the generality of its underlying model and its ability to perform well FAbatch represents a reliable tool for batch effect adjustment for most situations found in practice.

**Electronic supplementary material:**

The online version of this article (doi:10.1186/s12859-015-0870-z) contains supplementary material, which is available to authorized users.

## Background

In practical data analysis, the observations included in a dataset sometimes form distinct groups—denoted as “batches”; for example, measured at different times, under different conditions, by different persons or even in different labs. Such batch data is common in the context of high-throughput molecular data analysis, where experimental conditions typically have a high impact on the measurements and only few patients are considered at a time. Taking a more general point of view, different batches may also represent different studies concerned with the same biological question of interest. Independently of the particular scenario, in this paper all systematic differences between batches of data not attributable to the biological signal of interest are denoted as batch effects. If ignored when conducting analyses on the combined data, batch effects can lead to distorted and less precise results.

It is clear that batch effects are more severe when the sources from which the individual batches originate are more disparate. Batch effects—in our definition—may also include systematic differences between batches due to biological differences of the respective populations unrelated to the biological signal of interest. This conception of batch effects is related to an assumption made on the distribution of the data of recruited patients in randomized controlled clinical trials (see, e.g., [1]). This assumption is that the distribution of the (metric) outcome variable may be different for the actual recruited patients than for the patients eligible for the trial, i.e. there may be biological differences, with one important restriction: the difference between the means in treatment and control group must be the same for recruited and eligible patients. Here, the population of recruited patients and the population of eligible patients can be perceived as two batches (ignoring that the former population is a—very small—subset of the latter) and the difference between the means of the treatment and control group would correspond to the biological signal.

Throughout this paper we assume that the data of interest is high-dimensional, i.e. there are more variables than observations, and that all measurements are (quasi-)continuous. Possible present clinical variables are excluded from batch effect adjustment. Various methods have been developed to correct for batch effects. See for example [2, 3] for a general overview and for an overview of methods suitable in applications involving prediction, respectively. Two of the most commonly used methods are ComBat [4], a location-and-scale batch effect adjustment method and SVA [5, 6], a non-parametric method, in which the batch effects are assumed to be induced by latent factors. Even though the assumed form of batch effects underlying a location-and-scale adjustment as done by ComBat is rather simple, this method has been observed to greatly reduce batch effects [7]. However, a location-and-scale model is often too simplistic to account for more complicated batch effects. SVA is, unlike ComBat, concerned with situations where it is unknown which observations belong to which batches. This method aims at removing inhomogeneities within the dataset that also distort its correlation structure. These inhomogeneities are assumed to be caused by latent factors. When the batch variable is known, it is natural to take this important information into account when correcting for batch effects. Also, it is reasonable here to make use of the data-cleaning ability of the latent factor-adjustment by applying it within batches. This has the effect of reducing such inhomogeneities within batches, which are unrelated to the biological signal of interest. By doing so it can be expected that the homogeneity of the data is further increased across batches as well.

In this paper we suggest a method, denoted as “FAbatch” in the following, where “FA” stands for “**F**actor **A**djustment”. The method combines the location-and-scale adjustment (as performed by ComBat) with data cleaning by latent factor adjustment (as performed by SVA). Care has to be taken in the latent factor estimation in the context of data-cleaning. Inhomogeneities within the dataset are naturally not only induced by sources of unwanted noise but also by the biological signal of interest. If one would not take this interference between batch effects and signal into account, removing the corresponding estimated latent factor loadings would lead to removing a large part of the biological signal of interest. An obvious, yet problematic way, of protecting the signal of interest would be to remove it temporarily before estimating the latent factors by regressing each of the variables in the dataset on the variable representing the biological signal. However, this can lead to an artificially increased signal, as outlined in the Section “FAbatch”. As a solution for the case of a binary variable representing the biological signal, in our method we fit preliminary *L*_2_-penalized logistic regression models and use them to predict the probabilities of the individual observations to belong to the first and the second class, respectively. These predicted probabilities are then used in place of the actual values of the binary variable when protecting the signal of interest during latent factor estimation. See the Section “FAbatch” for details. In its current form our method is thus only applicable when the signal variable is binary, but extensions to other types of variables are possible, see the Section “Discussion”.

**Fig. 1:**
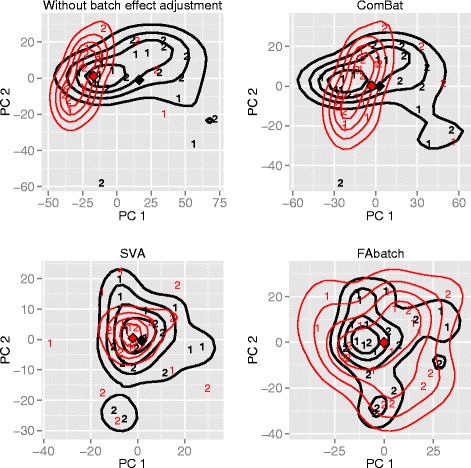
Visualization of batch effect adjustment. First two principal components out of PCA performed on the covariate matrix of a microarray dataset studying alcoholism: raw, after batch effect adjustment according to ComBat, SVA using three factors and FAbatch using three factors. The first batch is depicted in bold and the numbers distinguish the two classes “alcoholic” (2) vs. “healthy control” (1). The contour lines represent batch-wise two-dimensional kernel estimates and the diamonds represent the batch-wise centers of gravities of the points

As an illustration, Fig. 1 shows plots of the first two principal components obtained by Principal Component Analysis (PCA) on a raw dataset (upper-left) and after running the three different batch effect adjustment methods described above, respectively. The dataset, composed of two batches, contains the gene expressions of 20 alcoholics and 19 healthy controls. It is downloadable from ArrayExpress [8], accession number: E-GEOD-44456. After ComBat adjustment, the centers of gravity of the first principal components separated into the two batches become very similar (upper-right panel). However, the shapes of the point clouds corresponding to the two batches do not change substantially in comparison to the results obtained on the raw data (upper-left panel) and the two clouds do not fully overlap. After SVA adjustment—as with ComBat—the two batch centers are also similar (lower-left panel). The forms of the point clouds change more strongly compared to ComBat. Nevertheless, there are still regions in the plots with suboptimal overlap between the two clouds. The two batch centers are not distinguishable in the plot showing the result obtained after applying our method (lower-right panel). Moreover, the overlap between the two clouds is very high. This illustrative example suggests that the adjustment for batch effects can be improved by combining location-scale-adjustment with data-cleaning by factor adjustment.

An important area of application for high-throughput molecular data is the prediction of phenotypes via so-called prediction rules. Here, the training data used to obtain the prediction rule often constitutes a different batch than the validation data the prediction rule is applied to. Batch effect adjustment can be used here to make the validation data more similar to the training data before applying a prediction rule that was previously fitted on the training data. Such a procedure, termed “addon batch effect adjustment” in the following, is not specific to our method, but a general concept. Here, batch effect adjustment is first conducted based on the available original dataset. Some methods require that the values of the target variable are known in this dataset. Subsequently, batch effect adjustment for independent batches is performed. To facilitate this, it is required that several observations from each batch are available simultaneously (“frozen SVA” is an exception here, see the Section “Addon adjustment of independent batches”). This second phase does not affect the data prepared in the first phase. See the Section “Addon adjustment of independent batches” for details. We refer to such scenarios as cross-batch prediction in the rest of this paper. Our new FAbatch method allows such an addon batch effect adjustment.

The structure of this paper is as follows: In the Section “Methods” we introduce our new approach and treat addon batch effect adjustment. Moreover, we present the design of an extensive comparison study based on simulations and real data applications. In this study our method is compared with commonly used competitors with respect to diverse metrics measuring the effectiveness of batch effect adjustment [2, 9]. Our main focus lies in studying the performance of FAbatch here, but the results of this comparison study can also be used to aid researchers in choosing appropriate batch effect adjustment methods for their applications. The considered methods are: FAbatch (fabatch), ComBat (combat), SVA (sva), mean-centering (meanc), standardization (stand), ratio-A (ratioa) and ratio-G [3] (ratiog). The results of this study are described in the Section “Results”. In this section we also present an analysis demonstrating the use of batch effect adjustment methods in cross-batch prediction. Moreover, we argue that SVA can lead to an artificial increase of the biological signal of interest and demonstrate this using simulated data. The Section “Discussion” mostly reviews the models behind FAbatch and other approaches, and the Section “Conclusions” summarizes important conclusions from the paper.

## Methods

### FAbatch

#### Model

We assume the following model for the observed data *x*_*ijg*_:

(1) $$\begin{array}{*{20}l} &x_{ijg} = \alpha_{g} + \boldsymbol{a}_{ij}^{T} \boldsymbol{\beta}_{g} + \gamma_{jg} + \sum_{l=1}^{m_{j}} b_{jgl} Z_{ijl} + \delta_{jg} \epsilon_{ijg},\\ &Z_{ij1},\ldots,Z_{ij{m_{j}}} \sim N(0,1), \quad \epsilon_{ijg} \sim N(0, {\sigma_{g}^{2}}), \end{array} $$

Here *i* is the index for the observation, *j* the index for the batch and *g* the index for the variable. The term $\boldsymbol {a}_{\textit {ij}}^{T}\boldsymbol {\beta }_{g}$ parametrizes the effect of experimental conditions or, in general, any factors of interest ***a***_*ij*_ on the measurements of variable *g*. In this paper, ***a***_*ij*_ is a dummy variable representing the binary variable of interest *y*_*ij*_, with ***a***_*ij*_=1 if *y*_*ij*_=2 and ***a***_*ij*_=0 if *y*_*ij*_=1, respectively. The term *ε*_*ijg*_ represents random noise, unaffected by batch effects. The term *γ*_*jg*_ corresponds to the mean shift in location of variable *g* in the *j*-th batch compared to the unobserved—hypothetical—data $x^{*}_{\textit {ijg}}$ unaffected by batch effects. The term *δ*_*jg*_ corresponds to the scale shift of the residuals for variable *g* in the *j*-th batch. As in the SVA model (Appendix A.2, Additional file 1), *Z*_*ijl*_ are random latent factors. In contrast to the latter model, in our model the distribution of the latent factors is independent of the individual observation. However, since the loadings *b*_*jgl*_ of the latent factors are batch-specific, the latter induce batch effects in our model as well. More precisely, they lead to varying correlation structures in the batches. In the SVA model, by contrast, all batch effects are induced by the latent factors. Without the summand $\sum _{l=1}^{m_{j}} b_{\textit {jgl}} Z_{\textit {ijl}}$ model (1) would equal the model underlying the ComBat-method, see Appendix A.1 (Additional file 1).

The unobserved data $x^{*}_{\textit {ijg}}$ not affected by batch effects is assumed to have the form

(2) $$ x^{*}_{ijg} = \alpha_{g} + \boldsymbol{a}_{ij}^{T}\boldsymbol{\beta}_{g} + \epsilon_{ijg}, \quad \epsilon_{ijg} \sim N(0, {\sigma_{g}^{2}}).  $$

The remaining batch effect adjustment methods considered in this paper are described in Appendix A.3 (Additional file 1).

#### Using estimated probabilities instead of actual classes

As already noted in the Section “Background”, a further peculiarity of our method is that we do not use the actual classes when protecting the biological signal of interest in the estimation algorithm. Instead, we estimate the probabilities of the observations to belong to either class and use these in place of the actual classes, see the next paragraph and the next subsection for details.

This procedure has two major advantages. Firstly, it makes the batch effect correction method applicable to prediction problems involving new test observations with unknown classes. Secondly, using the actual classes might lead to an artificial increase of separation between the two classes in the dataset. This is because, as will be seen in the next subsection, it is necessary to use the estimated, instead of the true, but unknown, class-specific means when centering the data before factor estimation. Due to sampling variance, these estimated class-specific means often lie further away from each other than the true means, in particular for variables for which the true means lie close to each other. Subtracting the estimated factors’ influences leads to a reduction of the variance. Now, if centering the variable values within the classes before factor estimation, removing the estimated factor influences would lead to a reduction of the variance around the respective estimated class-specific means. In those—frequently occurring—cases, in which the estimated class-specific means lie further from each other than the corresponding true means, this would lead to an artificial increase of the discriminatory power of the corresponding variable in the adjusted dataset.

All analyses which are concerned with the discriminatory power of the covariate variables with respect to the target variable would be biased if performed on data adjusted in this way. More precisely, the discriminatory power would be overestimated. This mechanism is conceptually similar to the over-fitting of prediction models on the data they were obtained on. SVA suffers from a very similar kind of bias, also related to using the class information in protecting the biological signal. See the Section “Artificial increase of measured class signal by applying SVA” for a detailed description of this phenomenon and the results of a small simulation study performed to assess the impact of this bias on data analysis in practice.

The probabilities of the observations to belong to either class, that are considered in FAbatch, are estimated using models fitted from data other than the corresponding observations. Using these probabilities instead of the actual classes attenuates the artificial increase of the class signal described above. The idea underlying the protection of the signal of interest is to center *x*_*ijg*_ before factor estimation by subtracting the term

(3) $$\begin{array}{*{20}l} &\mathbb{E}(\alpha_{g} + \boldsymbol{a}_{ij}^{T} \boldsymbol{\beta}_{g} + \gamma_{jg} | x_{ij1,},\dots,x_{ijp}) =\\ &\quad \text{Pr}(y_{ij}=1 | x_{ij1,},\dots,x_{ijp}) \; (\alpha_{g} + \gamma_{jg}) + \\ &\quad \text{Pr}(y_{ij}=2 | x_{ij1,},\dots,x_{ijp}) \; (\alpha_{g} + \boldsymbol{\beta}_{g} + \gamma_{jg}). \end{array} $$

Note that we perform this adjustment slightly differently in the FAbatch-estimation algorithm. See the next subsection for details.

#### Estimation

In the following we detail the estimation procedure of FAbatch:

1. Standardize the values *x*_*ijg*_ per batch:
  (4) $$ x_{ijg,S} := \frac{x_{ijg} - \widehat{\mu_{jg}}}{\sqrt{\widehat{\sigma_{jg}^{2}}}},  $$
  where $\widehat {\mu _{\textit {jg}}} = (1/n_{j}) \sum _{i} x_{\textit {ijg}}$ and $\widehat {\sigma _{\textit {jg}}^{2}} = [1/(n_{j} - 1)] \sum _{i} (x_{\textit {ijg}} - \widehat {\mu _{\textit {jg}}})^{2}$. Here, the number of observations in batch *j* is denoted as *n*_*j*_.
2. Using *L*_2_-penalized logistic regression, for each observation estimate the probability to belong to class 2:
  (5) $$ \widehat{\pi_{ij}} := \widehat{\text{Pr}}(y_{ij}=2 | x_{ij1,S},\dots,x_{ijp,S}).  $$
  Here, we employ the following cross-validation related procedure. For batch *j*∈{1,…,*K*}: 1) Fit a *L*_2_-penalized logistic regression model using all observations except those in batch *j*; 2) Use the model fitted in step 1) to predict the probabilities *π*_*ij*_ of the observations from batch *j*. By using different observations for fitting the models than for predicting the probabilities we avoid overfitting in the sense of the problems occurring when the actual classes are used as described in the previous subsection. The reason why we perform cross-batch prediction for estimating the probabilities here instead of ordinary cross-validation is that we expect the resulting batch adjusted data to be more suitable for the application in cross-batch prediction (see the Section “Addon adjustment of independent batches”). Here, for estimating the probabilities in the test batch we have to use a prediction model fitted on other batches. If the probabilities in the training data were estimated via ordinary cross-validation they would be more optimistic—i.e. closer to zero and one, respectively—than those in the test data. This is because in ordinary cross-validation it can occur that observations from the same batch are in training and test data. By doing cross-batch prediction for the estimation of the *π*_*ij*_ we mimic the situation encountered in cross-batch prediction applications. The only, but important, exception where we perform ordinary cross-validation for estimating the *π*_*ij*_ is when the data come from only one batch (this occurs in the context of cross-batch prediction, when the training data consist of one batch).
  The shrinkage intensity tuning parameter of the *L*_2_-penalized logistic regression model is optimized with the aid of cross-validation [10]. For computational efficiency this optimization is not repeated in each iteration of the cross-batch prediction. Instead, it is performed beforehand on the complete dataset. The overoptimism resulting from this procedure compared to that of the gold-standard technique “nested cross-batch prediction” can be assumed to be negligible in the considered context.
3. Calculate the class adjusted values *x*_*ijg*,*S*,*CA*_, which should contain considerably less class signal than *x*_*ijg*,*S*_:
  (6) $$ x_{ijg,S,CA} := x_{ijg,S} - (1 - \widehat{\pi_{ij}})\widehat{\mu_{g, S}}^{(1)} - \widehat{\pi_{ij}}\widehat{\mu_{g, S}}^{(2)},  $$
  where $\widehat {\mu _{g, S}}^{(c)} = (1/\# L_{c}) \sum _{\{i^{*},j^{*}\} \in L_{c}} x_{i^{*}j^{*}g, S}$ with *L*_*c*_={{*i*,*j*}:*y*_*ij*_=*c*,*i*∈{1,…,*n*_*j*_},*j*∈{1,…,*J*}} and *c*∈{1,2}.
4. Using *x*_*ijg*,*S*,*CA*_, estimate the latent factors $Z^{*}_{ij{m_{j}}}$ and their loadings $b^{*}_{jg{m_{j}}}$ by an EM-algorithm presented in [11], again considered by Friguet et al. [12] in a specific context for microarray data. For the estimation of the number of factors see [12].
5. Subsequently the estimated factor contributions are removed:
  (7) $$ x_{ijg,S,FA} := x_{ijg,S} - \widehat{b^{*}_{jg1}} \widehat{Z^{*}_{ij1}} - \dots - \widehat{b^{*}_{jg{m_{j}}}} \widehat{Z^{*}_{ij{m_{j}}}},  $$
  where $\widehat {b^{*}_{jg1}}, \dots, \widehat {b^{*}_{jg{m_{j}}}}$ are the estimated, batch-specific factor loadings and $\widehat {Z^{*}_{ij1}}, \dots, \widehat {Z^{*}_{ij{m_{j}}}}$ are the estimated latent factors. Note that only the factor contributions as a whole are identifiable, not the individual factors and their coefficients.
6. Finally, in each batch the *x*_*ijg*,*S*,*FA*_-values are transformed to have the global means and pooled variances estimated before batch effect adjustment:
  (8) $$ \widehat{x^{*}_{ijg}} = \left(\frac{x_{ijg,S,FA} - \widehat{\mu_{g,S,FA}}}{\sqrt{\widehat{\sigma_{g,S,FA}^{2}}}}\right) \sqrt{\widehat{{\sigma_{g}^{2}}}} + \widehat{\mu_{g}},  $$
  $$\begin{array}{*{20}l} & \text{where} && \widehat{\mu_{g,S,FA}} = \left(1/\sum_{j} n_{j}\right) \sum_{j} \sum_{i} x_{ijg,S,FA},\\ & && \widehat{\sigma_{g,S,FA}^{2}} = \left[1/\left(\sum_{j} n_{j} - 1\right)\right] \\ & && \quad \sum_{j} \sum_{i} (x_{ijg,S,FA} - \widehat{\mu_{g,S,FA}})^{2},\\ & && \widehat{\mu_{g}} = \left(1/\sum_{j} n_{j}\right) \sum_{j} \sum_{i} x_{ijg}\\ & \text{and} && \widehat{{\sigma_{g}^{2}}} \,=\, \left[\!\!1/\!\left(\!\!\sum_{j} n_{j} \,-\, 1\!\right)\!\!\right] \sum_{j} \sum_{i} (x_{ijg} \,-\, \widehat{\mu_{g}})^{2}. \end{array} $$
  Note that by forcing the empirical variances in the batches to be equal to the pooled variances estimated before batch effect adjustment we overestimate the residual variances ${\sigma _{g}^{2}}$ in (1). This is because we do not take into account that the variance is reduced by the adjustment for latent factors. However, unbiasedly estimating ${\sigma _{g}^{2}}$ appears difficult due to the scaling before estimation of the latent factor contributions.

#### Verification of model assumptions on the basis of real data

Due to the flexibility of its model FAbatch should adapt well to real datasets. Nevertheless it is important to check its validity based on real data, because the behaviour of high-dimensional biomolecular data does not become apparent by mere theoretical considerations. Therefore, we demonstrate that our model is indeed suited for such data using the dataset BreastCancerConcatenation from Table 1. This dataset was chosen because here the batch effects can be expected to be especially strong due to the fact that the batches involved in this dataset are themselves independent datasets. We obtained the same conclusions for other datasets (results not shown). Because our model is an extension of the ComBat-model by batch-specific latent factor contributions, we compare the model fit of FAbatch to that of ComBat.

**Table 1:** Overview of datasets used in empirical studies

Label	Num. of	Num. of	Num. of	Prop. with	Data type	Source (Acc.num.)
	observ.	batches	variables	y = 2		
ColonGastricEsophagealcSNPArray	93	3	50000	0.54	comparative genomic hybridization	ArrayExpr.: E-GEOD-36458
AgeDichotomTranscr	243	15	27568	0.49	DNA methylation profiling	ArrayExpr.: E-GEOD-36194
EthnicityMethyl	133	3	50000	0.45	DNA methylation profiling	ArrayExpr.: E-GEOD-39672
BipolardisorderMethyl	94	2	27537	0.50	DNA methylation profiling	ArrayExpr.: E-GEOD-38873
PostpartumDepressionMethyl	50	5	50000	0.46	DNA methylation profiling	ArrayExpr.: E-GEOD-44132
AutismTranscr	439	5	24526	0.53	transcription profiling	ArrayExpr.: E-GEOD-37772
BreastcTranscr	410	23	20180	0.50	transcription profiling	ArrayExpr.: E-GEOD-44281
BreastCancerConcatenation	168	5	22277	0.65	transcription profiling	ArrayExpr.: E-GEOD-27562,
						E-GEOD-21422, E-GEOD-22544,
						E-GEOD-20266, E-TABM-276
IUGRTranscr	67	2	48701	0.40	transcription profiling	ArrayExpr.: E-GEOD-35574
IBSTranscr	63	6	54671	0.70	transcription profiling	ArrayExpr.: E-GEOD-36701
SarcoidosisTranscr	58	3	54675	0.66	transcription profiling	NCBI GEO: GSE19314
pSSTranscr	49	3	54675	0.63	transcription profiling	ArrayExpr.: E-GEOD-40611
AlcoholismTranscr	39	2	28869	0.51	transcription profiling	ArrayExpr.: E-GEOD-44456
WestNileVirusTranscr	39	2	47323	0.46	transcription profiling	ArrayExpr.: E-GEOD-43190

The following information is given: number of observations, number of batches, number of variables, proportion of observations with disease, biomolecular data type, accession number

Additional file 1: Figure S1 and Figure S2 show, for each batch, a plot of the data values against the corresponding fitted values of FAbatch and ComBat respectively. While there seem to be no deviations in the mean for both methods, the association between data values and predictions is a bit stronger for FAbatch—except in the case of batch 4. This stronger association between fitted values and predictions for FAbatch can be explained by the fact that the factor contributions absorb part of the variance of the data values. In the case of batch 4, the estimated number of factors was zero, explaining why the variance is not reduced here in comparison to ComBat. Additional file 1: Figure S3 and Figure S4 correspond to the previous two figures, except that here the deviations from the fitted values instead of the data values are plotted against the corresponding fitted values. We observe that for batches 2, 3 and 5 the variance of these residuals depends slightly less on the mean for FAbatch in comparison to ComBat. Batchwise density estimates of these residuals divided by their standard deviations are shown in Additional file 1: Figure S5 and Figure S6 for FAbatch and ComBat, respectively. For both methods outliers are observed. However, the distributions of the residuals differ between the two methods. In the case of ComBat the distributions are skewed for part of the batches, slightly for batches 3 and 5 and more strongly for batch 2. In the case of FAbatch the distributions are symmetric. A probable reason for the skewness of the distributions in the case of ComBat is that the residuals still contain the biological signal, as it is not included in the fixed part of the model.

### Addon adjustment of independent batches

As already described in the Section “Background”, an important feature of batch effect adjustment methods is that they offer the possibility of making validation data more similar to training data of the same kind studying the same biological question of interest. Here, the training and the validation data may themselves each consist of different batches. This feature of batch effect adjustment can be used for prediction purposes in particular. In the following we detail how batch effect adjustment is conceptionally performed for incorporating independent batches in general and treat the respective procedures for the particular methods considered in this paper.

#### General procedure

A batch effect adjustment method (implicitly or explicitly) assumes a specific model for the observed data. One part of parameters involved in this model is connected with the observed data within the batches *x*_*ijg*_ and another part with the unobserved batch effect free data $x^{*}_{\textit {ijg}}$. While the values of the former kind of parameters in most cases depend on the individual batches, the latter kind are the same for all observations, i.e. these are batch-unspecific. When incorporating independent batches after having adjusted the training data, we are interested in transforming the data in the independent batches in such a way that its distribution becomes similar to that of the already adjusted training data without having to change the latter. This is achieved by performing the same kind of transformation on the independent batches with the peculiarity that for the involved batch-unspecific parameters the estimates obtained on the training data are used. We refer to these procedures as addon batch effect adjustment procedures.

Using the above definition, for those batch effect adjustment methods, for which the corresponding adjustment does not involve estimated batch-unspecific parameters, the addon procedure is the same as the corresponding batch effect adjustment method. From the batch effect adjustment methods considered in this paper, this is the case for mean-centering, standardization, ratio-A and ratio-G. Here the batch effect adjustment is performed batch by batch. The adjustment according to ComBat, FAbatch and SVA, respectively, does by contrast involve estimated batch-unspecific parameters.

#### ComBat

For ComBat, Luo et al. [3] present the addon procedure for the situation of having only one batch in the training data. The addon batch effect adjustment with ComBat consists of applying the standard ComBat-adjustment to the validation data without the term $\boldsymbol {a}_{\textit {ij}}^{T}\boldsymbol {\beta }_{g}$ and with all batch-unspecific parameters *α*_*g*_, ${\sigma _{g}^{2}}$ and ***β***_*g*_ estimated using the training data.

M-ComBat [13] is a similar method, applicable in the situation of one batch in the training data. This method can be seen to perform a location-and-scale adjustment of the validation data, i.e., in contrast to original ComBat, this method does not use shrinkage by empirical Bayes. According to our definition of addon batch effect adjustment from the previous subsection, M-ComBat thus represents the addon batch effect adjustment procedure for the following method: location-and-scale batch effect adjustment when having one batch in the training data.

#### FAbatch

The adjustment with FAbatch involves estimates of the same batch-unspecific parameters as that with ComBat (according to Eq. (1)): *α*_*g*_, ${\sigma _{g}^{2}}$ and ***β***_*g*_. However, unlike in the adjustment with ComBat, in FAbatch the term $\boldsymbol {a}_{\textit {ij}}^{T}\boldsymbol {\beta }_{g}$ is considered additionally. This is achieved—roughly—by estimating $\mathbb {E}(\boldsymbol {a}_{\textit {ij}} | x_{ij1,},\dots,x_{\textit {ijp}})$ and ***β***_*g*_ using *L*_2_-penalized logistic regression. See again the Section “Estimation” for details. The addon procedure for FAbatch is straightforwardly derived from the general definition of addon procedures given above: the estimation scheme in the Section “Estimation” is performed with the peculiarity that for all occurring batch-unspecific parameters, the estimates obtained in the adjustment of the training data are used.

#### SVA

For SVA there exists a specific procedure denoted as “frozen SVA” [6], abbreviated as “fSVA,” for preparing independent data for prediction. More precisely, Parker et al. [6] describe two versions of fSVA: the “exact fSVA algorithm” and the “fast fSVA algorithm”. In Appendix A.2.1 we demonstrate that the “fast fSVA algorithm” corresponds to the addon procedure for SVA.

In the fSVA algorithms the training data estimated factor loadings (and other informations in the case of the fast fSVA algorithm) are used. This requires that the same sources of heterogeneity are present in training and test data, which might not be true for a test data batch from a different source. Thus, frozen SVA is only fully applicable when training and test data are similar, as stated by Parker et al. [6]. Nevertheless in the Section “Application in cross-batch prediction” we apply it in cross-batch prediction to obtain indications on whether the prediction performance of classifiers might even deteriorate through the use of frozen SVA when training and test data are very different.

Above we have presented the addon procedures for the batch effect adjustment methods that are considered in this paper. However, using our general definition of addon procedures, such algorithms can readily be derived for other methods as well.

### Comparison of FAbatch with existing methods

A comprehensive evaluation of the ability of our method to adjust for batch effects in comparison to its competitors was performed—using both simulated as well as real datasets. The simulation enables us to study the performance, subject to basic settings and to use a large number of datasets. Nevertheless simulated data can never capture all properties found in real datasets from the area of the application. Therefore, in addition, we studied 14 publicly available real datasets, each consisting of at least two batches.

The value of batch effect adjustment contains different aspects, which are connected with the adjusted data itself or with the results of certain analyses performed using the latter. Therefore, when comparing batch effect adjustment methods it is necessary to consider several criteria, where each is concerned with a certain aspect. We calculated seven different metrics measuring the performance of each batch effect adjustment method on each simulated and each real dataset.

In the following, we first outline the seven metrics considered in the comparison study described above. Subsequently, we introduce the simulation designs and give basic information on the real datasets. The results of these analyses are presented and interpreted in the Section “Ability to adjust for batch effects”.

#### Performance metrics

Here we describe the performance metrics used to assess batch effect adjustment. Several of them are, in their original form, restricted to the case of only two batches. For datasets with more than two batches they are extended as follows: 1) Calculate the original metric for all possible pairs of batches; 2) Calculate the weighted average of the values in 1) with weights proportional to the sum of the sample sizes in the two respective batches.

##### Separation score (sepscore)

We derived this metric from the mixture score presented in [2]. The latter was not applicable here, because it depends on the relative sizes of the two involved batches *j* and *j*^∗^. Roughly speaking the mixture score measures the degree of mixing between the observations belonging to the two batches after batch effect adjustment. The separation score by contrast measures the degree of separation between the two batches. At first for each observation in *j*, its *k* nearest neighbours are determined in both batches simultaneously with respect to the euclidean distance. Here, the proportion of nearest neighbours belonging to batch *j*^∗^ is calculated. Then the average—denoted as MS_*j*_—is taken over the *n*_*j*_ proportions obtained in this way. This value is the mixture score as in [2]. To obtain a measure for the separation of the two batches the absolute difference between MS_*j*_ and its value expected in the absence of batch effects is taken: $| \text {MS}_{j} - n_{j^{*}} /(n_{j} + n_{j^{*}} - 1) |\phantom {\dot {i}\!}$. The separation score is defined as the simple average of the latter quantity and the corresponding quantity when the roles of *j* and *j*^∗^ are switched. The number *k* of nearest neighbours considered was set to 10. Smaller values of the separation score are better.

##### Average minimal distance to other batch (avedist)

A very similar metric for two batches is the average minimal distance to the other batch after batch effect adjustment, see also [2]. For each observation in batch *j* the euclidean distance to the nearest observation in batch *j*^∗^ is calculated. Consecutively the roles of *j* and *j*^∗^ are switched and finally the average is computed over all $n_{j} + n_{j^{*}}\phantom {\dot {i}\!}$ minimal distances. To obtain a metric independent of the scale, we standardize the variables before the calculation to have zero mean and uniform variance. Here, smaller values are better.

##### Kullback-Leibler divergence between density of within and between batch pairwise distances (klmetr)

This metric, used in [9] in a similar form is again based on the distances of the observations within and between batches. At first the distances between all pairs of observations in batch *j*—denoted as {*dist*_*j*_}—and the distances between all such pairs in batch *j*^∗^—denoted as $\{{dist}_{j^{*}}\}\phantom {\dot {i}\!}$—are calculated. Then for each observation in *j* the distances to all observations in *j*^∗^ are calculated, resulting in $n_{j} \times n_{j^{*}}\phantom {\dot {i}\!}$ distances denoted as $\{{dist}_{j j^{*}}\}\phantom {\dot {i}\!}$. Consecutively we estimate the Kullback-Leibler divergence between the densities of {*dist*_*j*_} and $\{{dist}_{j j^{*}}\}\phantom {\dot {i}\!}$ and that between the densities of $\{{dist}_{j^{*}}\}\phantom {\dot {i}\!}$ and $\{{dist}_{j j^{*}}\}\phantom {\dot {i}\!}$—using the *k*-nearest neighbours based method by Boltz et al. [14] with *k*=5. Finally, we take the weighted mean of the values of these two divergences with weights proportional to *n*_*j*_ and $n_{j^{*}}\phantom {\dot {i}\!}$. As in the case of avedist the variables are standardized before the calculation to make the metric independent of scale. Smaller values of this metric are better.

##### Skewness divergence score (skewdiv)

This metric presented in [15] is concerned with the values of the skewness of the observation-wise empirical distributions of the data. Because batch effect adjustment should make the distribution of the data similar for all batches, these skewness values should not differ strongly across batches after a successful batch effect adjustment. The metric is obtained as follows for two batches *j* and *j*^∗^ after batch effect adjustment: 1) for each observation calculate the difference between the mean and the median of the data in batch *j* and *j*^∗^, respectively, as a measure for the skewness of the distribution of the variable values; 2) determine the area between the two batch-wise empirical cumulative density functions of the values out of 1). The value obtained in 2) can be regarded as a measure for the disparity of the batches with respect to the skewness of the observation-wise empirical distributions. Again, standardization is conducted before the calculation. Smaller values indicate a more successful batch effect adjustment with respect to the homogeneity of the skewness values.

##### Proportion of variation induced by class signal estimated by Principal Variance Components Analysis (pvca)

Principal Variance Component Analysis [16] allows the estimation of the contributions of several sources of variability. Here, first principal component analysis is performed on the *n*×*n* covariance matrix between the observations. Then, using a random effects model, the principal components are regressed on arbitrary factors of variability, such as “batch” and “(phenotype) class”. Ultimately, estimated proportions of variance induced by each factor, and that of the residual variance are obtained; for details see [16]. We included the factors “batch”, “class” and the interaction of these two into the model and used the proportion of variance explained by “class” as a metric. Naturally, higher values of this metric indicate a better preservation or exposure, respectively, of the biological signal of interest.

##### Performance of differential expression analysis (diffexpr)

This metric is similar to the idea presented in [2] which consists in comparing the list of genes deemed differentially expressed the strongest using a batch effect adjusted dataset to the corresponding list obtained using an independent dataset. Having no independent data available here we had to consider a slightly different approach: 1) For each batch *j* leave this batch out and perform batch effect adjustment using the rest of the dataset. Derive two lists of the 5 % of variables deemed differentially expressed the strongest (see next paragraph for details): one using the batch effect adjusted dataset—where batch *j* was left out—and one using the data from batch *j*. Calculate the number of variables appearing in both lists and divide this number by the common length of the lists. 2) Calculate a weighted average of the values obtained in 1) with weights proportional to the number of observations in the corresponding left-out batches. Note that in the case of the simulated datasets we would be able to estimate the true discovery rate instead of calculating the metric described above. However, for the sake of comparability, we applied the procedure described above for the simulated data as well.

Now we describe the procedure performed for estimating those 5 % of variables which are most differentially expressed. Our original idea to use the p-values of simple two-sample t-tests between the two classes was soon discarded. The reason for this was that this procedure might have favoured batch effect adjustment methods that produce more normally distributed values of the variables. The p-values of classical non-parametric tests, such as the Mann-Whitney-Wilcoxon rank sum test would also not have been suitable here, because of the fact that here the p-values can only adopt a limited number of possible values. Therefore, it would have occurred in many cases that more than 5 % of the variables adopt the smallest of possible p-values, making a selection of 5 % of variables with the smallest p-values impossible. As a solution, for each variable we drew a randomized p-value out of the Whitney-Wilcoxon rank sum test, see [17] for details. These randomized p-values can adopt any possible value between zero and one and were consequently suitable for ordering the variables according to their degree of differential expression between the two classes. We ultimately considered those 5 % variables that were associated with the smallest p-values. Higher values of this metric are better.

##### Mean Pearson’s correlation of the variable values before and after batch effect adjustment (corbeaf)

This metric suggested by Lazar et al. [2] is not a measure for the performance of batch effect adjustment. However, it may be used occasionally to decide between two methods performing similarly: in such cases the method that least affects the data—i.e. that with smaller corbeaf-values—could be preferred [2].

#### Simulation design

Three basic scenarios were considered: 1) “ComCor”: Common correlation structure in all batches; 2) “BatchCor”: Batch-specific correlation structures; 3) “BatchClassCor”: Batch- and class-specific correlation structures. For each of these the correlations were induced in two ways (see below for details): 1) simulating from a latent factor model with normally distributed residuals; 2) drawing from multivariate normal distributions with specified correlation matrices. The second scheme was considered to avoid favouring FAbatch and SVA by restricting the simulation to factor-based data generation mechanisms. We simulated datasets consisting of four batches with 25 observations each. The number of variables was 1000. For each of the six (3 × 2) settings 500 datasets were simulated. The values of the parameters occurring in the simulation models were based on corresponding estimates obtained from two publicly available microarray datasets: a dataset also used in the real data study, denoted as AutismTranscr (Table 1) and a dataset studying colon cancer, denoted as ColoncbTranscr. The latter is downloadable from ArrayExpress [8], accession number: E-GEOD-44861.

All six settings can be expressed using the following most general model:

(9) $$\begin{array}{*{20}l} &\boldsymbol{x}_{ij} = \boldsymbol{\alpha} + \boldsymbol{a}_{ij}\boldsymbol{\beta} + \boldsymbol{\gamma}_{j} + \boldsymbol{\epsilon}^{*}_{ij},\\ &\boldsymbol{\epsilon}^{*}_{ij} \sim MVN(\boldsymbol{0}, \boldsymbol{\Sigma}_{j,\boldsymbol{a}_{ij}}), \end{array} $$

with ***x***_*ij*_=(*x*_*ij*1_,…,*x*_*ijp*_)^*T*^, ***α***=(*α*_1_,…,*α*_*p*_)^*T*^, ***a***_*ij*_∈{0,1}, ***β***=(*β*_1_,…,*β*_*p*_)^*T*^, ***γ***_*j*_=(*γ*_*j*1_,…,*γ*_*jp*_)^*T*^, $\boldsymbol {\epsilon }^{*}_{\textit {ij}} = (\epsilon ^{*}_{ij1},\dots,\epsilon ^{*}_{\textit {ijp}})^{T}$, *j*∈{1,…,*K*} and *p*=1000.

The entries of ***α*** and ***γ***_*j*_ (*j*∈{1,…,*K*}) were drawn from normal distributions with means and variances based on corresponding estimates obtained from ColoncTranscr. For details see the corresponding commented R code provided in Additional file 2. The vector of the class differences ***β*** contains 300 (30 %) non-zero values. Half of these are negative and half positive. The values were drawn from gamma distributions, where the choice of parameters was again based on ColoncTranscr. Here, in the case of the negative entries of ***β***, the sign of the originally drawn values was changed.

The six settings differ with respect to the specification of $\boldsymbol {\Sigma }_{j,\boldsymbol {a}_{\textit {ij}}}\phantom {\dot {i}\!}$. The differences are outlined in the following.

##### Design A: Simulating from latent factor model

The residuals of the fixed part of the model $\boldsymbol {\epsilon }^{*}_{\textit {ij}}$ were simulated in the following ways for the corresponding scenarios:

(10) $$\begin{array}{*{20}l} &\text{1. ComCor:} & \epsilon^{*}_{ijg} :=& \sum_{m=1}^{5} b_{0gm} Z_{ijm} + \delta_{jg} \epsilon_{ijg} \end{array} $$

(11) $$\begin{array}{*{20}l} &\text{2. BatchCor:} & \epsilon^{*}_{ijg} :=& \sum_{m=1}^{5} b_{0gm} Z_{ijm} + \\ &&& \sum_{m=1}^{5} b_{jgm} \overset{*}{Z}_{ijm} + \delta_{jg} \epsilon_{ijg} \end{array} $$

(12) $$\begin{array}{*{20}l} &\text{3. BatchClassCor:} & \epsilon^{*}_{ijg} :=& \sum_{m=1}^{5} b_{0gm} Z_{ijm} + \\ &&& \sum_{m=1}^{5} \widetilde{b}_{\boldsymbol{a}_{ij}gm} Z_{ijm} + \\ &&& \sum_{m=1}^{5} b_{jgm} \overset{*}{Z}_{ijm} + \delta_{jg} \epsilon_{ijg}, \end{array} $$

where $\epsilon _{\textit {ijg}} \stackrel {iid}{\sim } N(0, {\sigma _{g}^{2}})$ and $Z_{\textit {ijm}},\; \overset {*}{Z}_{\textit {ijm}} \stackrel {iid}{\sim } N(0,1)$. *b*_0*gm*_, *b*_*jgm*_ and $\widetilde {b}_{\boldsymbol {a}_{\textit {ij}}gm}$ were drawn from normal distributions and $\delta _{\textit {jg}}^{2}$ and ${\sigma _{g}^{2}}$ from inverse gamma distributions. The parameters of the latter distributions are again based on corresponding estimates obtained from ColoncTranscr.

In Eqs. (10), (11) and (12) the factors *Z*_*ij*1_,…,*Z*_*ij*5_ model the biological correlation between the variables. The factors $\overset {*}{Z}_{ij1},\dots,\overset {*}{Z}_{ij5}$ in (11) and (12) model distortions that affect the correlation in the batches. In setting “ComCor” all observations have the same correlation structure—independent of the batch. In setting “BatchCor” the correlation structure is different in each batch, due to the batch-specific loadings of the factors $\overset {*}{Z}_{ij1},\dots,\overset {*}{Z}_{ij5}$. In the third setting, “BatchClassCor”, the correlations differ not only by batch but also according to which of the two classes the observations are in, i.e. we have batch- and class-specific correlations. In each setting the variances are different in the batches.

##### Design B: Drawing from multivariate distributions with specified correlation matrices

In Design B, all correlation matrices appearing in the three scenarios were estimated using real data. Here we first calculated the approximate positive definite correlation matrix using the R function cor and then applied the R function nearPD from the R package Matrix to the result to calculate the nearest positive definite correlation matrix. We used the 1000 genes from the AutismTranscr dataset, which showed themselves to be most related to the binary outcome according to variable-wise two-sample t-tests. Before estimating the correlation matrices, the data was further centered by class in each batch to adjust for excess correlations due to class differences. The variances are the same in all three scenarios. They were set to be equal to those in scenario “ComCor” of Design A, i.e. $\sum _{m=1}^{5} b_{0gm}^{2} + \delta _{\textit {jg}}^{2} {\sigma _{g}^{2}}$.

The correlation matrices were obtained as follows for the three scenarios:

1. ComCor: A single correlation matrix was used for all batches here. It was estimated from the data of a single batch in AutismTranscr.
2. BatchCor: A separate correlation matrix was used for each batch here, each estimated from the data of a batch in AutismTranscr.
3. BatchClassCor: A separate correlation matrix was used for each combination of batch and class here, where each was estimated on a corresponding batch-class-combination in AutismTranscr.

After obtaining the correlation matrices, the corresponding covariance matrices were calculated. The latter was done by multiplying each entry in the correlation matrices with the respective pair of standard deviations.

#### Datasets

We used 14 high-dimensional datasets with a binary target variable and at least two batches. They were downloaded from the ArrayExpress database (www.ebi.ac.uk/arrayexpress) [8] or the NCBI GEO database (www.ncbi.nlm.nih.gov/geo) [18].

In searching for suitable datasets on ArrayExpress and NCBI GEO, we entered the search term “batch” and manually surveyed the search hits. This proceeding was chosen in order to maximise the number of possibly eligible datasets. Exclusion criteria were: number of samples too low, abscence of a batch variable, and impossibility of forming a suitable binary target variable. We state that the selection of the datasets was not in any way based on the results they yielded with the different methods, thus following Rule 4 from [19] (“do not fish for datasets”).

Three datasets featured too many variables to be manageable for our systems. Therefore, in these cases, we randomly selected 50,000 variables. When missing values occurred in the measurements of datasets we took the following approach. First, we excluded variables with too many missing values. Consecutively the remaining missing values were simply imputed by the median of the observed values of the corresponding variable in the corresponding batch. This simplistic imputation procedure can be justified by the very low numbers of variables with missing values in all datasets. Outlier analysis was performed by visually inspecting the principal components out of PCA applied to the individual datasets. Here, suspicious samples were removed. Additional file 1: Figure S7 shows the first two principal components out of PCA applied to each of the used datasets after imputation and outlier removal.

Table 1 gives an overview on the datasets. Information on the nature of the binary target variable is given in Appendix D (Additional file 1). The dataset BreastCancerConcatenation is a concatenation of five independent breast cancer datasets. For the remaining 13 datasets the reason for the batch structure could be ascertained in only four cases. In three of these, batches were due to hybridization and in one case due to labeling. For details see Appendix E (Additional file 1).

For further details regarding the background of the datasets and the preprocessing the reader may look up the accession numbers online and consult the corresponding R scripts, respectively, written for preparation of the datasets, which are available in Additional file 2. Here we also provide all R code necessary to reproduce our analyses.

## Results

### Ability to adjust for batch effects

**Fig. 2:**
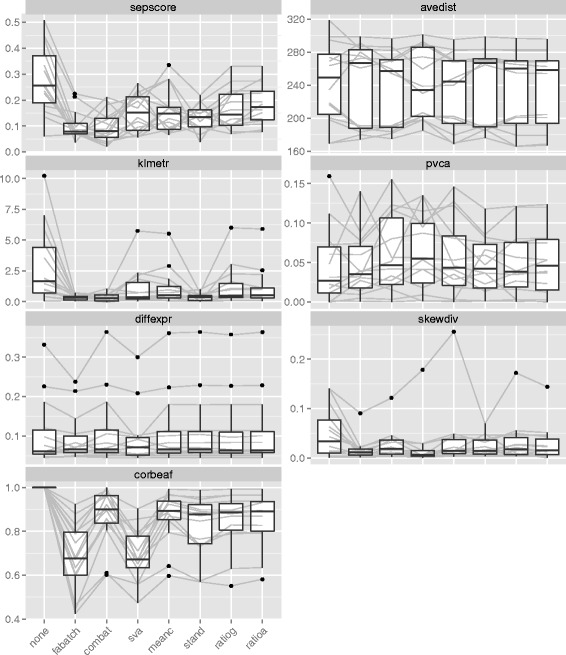
Metric values in real datasets. Boxplots of values for all 14 datasets separated into method for the following metrics: sepscore, avedist, klmetr, pvca, diffexpr, skewdiv and corbeaf. The grey lines connect values corresponding to the same datasets

Additional file 1: Figure S8 to S14 show the values of the individual metrics obtained on the simulated data and Fig. 2 shows the corresponding results obtained on the 14 real datasets. Additional file 1: Tables S1 to S7 for the simulated and Tables 2 and 3 for the real data, respectively show the means of the metric values separated by method (and simulation scenario) together with the mean ranks of the methods with respect to the individual metrics. In most cases, we observe that the simulation results differ only slightly between the settings with respect to the ranking of the methods by their performance. Therefore, we will only occasionally differentiate between the scenarios in the interpretations. Similarly, simulations and real data analyses often yield similar results. Differences will be discussed whenever relevant.

**Table 2:** Means of the metric values and of their ranks among the different methods over the 14 studied datasets separated into method for the following metrics: sepscore, avedist, klmetr and pvca

sepscore								
Mean values	combat	**fabatch**	stand	sva	meanc	ratiog	ratioa	none
	0.09895	**0.10227**	0.13238	0.15217	0.15807	0.16618	0.18314	0.2806
Mean ranks	combat	**fabatch**	stand	sva	meanc	ratiog	ratioa	none
	2.28571	**3.35714**	3.71429	4.42857	4.64286	4.78571	5.92857	6.85714
avedist								
Mean values	meanc	ratiog	ratioa	combat	stand	**fabatch**	sva	none
	233.32619	235.27321	235.39525	235.52757	237.86855	**239.53197**	240.55365	243.10948
Mean ranks	meanc	combat	ratiog	ratioa	**fabatch**	stand	none	sva
	3.07143	3.57143	3.57143	3.57143	**5.14286**	5.21429	5.78571	6.07143
klmetr								
Mean values	**fabatch**	combat	stand	sva	meanc	ratioa	ratiog	none
	**0.32312**	0.33748	0.35524	1.08835	1.13029	1.15025	1.23577	2.85956
Mean ranks	combat	**fabatch**	stand	sva	meanc	ratioa	ratiog	none
	2.85714	**3**	3.14286	4.57143	4.71429	4.92857	5.42857	7.35714
pvca								
Mean values	sva	combat	meanc	ratioa	ratiog	stand	**fabatch**	none
	0.06364	0.06015	0.05636	0.0502	0.04933	0.04741	**0.04569**	0.04477
Mean ranks	sva	combat	meanc	ratioa	stand	ratiog	**fabatch**	none
	2.92857	3.14286	3.57143	5	5.07143	5.21429	**5.35714**	5.71429

In each row the results are listed in descending order according to mean performance in terms of the original values and their ranks, respectively. The results of FAbatch are printed in bold

**Table 3:** Means of the metric values and of their ranks among the different methods over the 14 studied datasets separated into method for the following metrics: diffexpr, skewdiv and corbeaf

diffexpr								
Mean values	combat	stand	ratioa	meanc	ratiog	none	sva	**fabatch**
	0.11044	0.10958	0.10891	0.1088	0.10796	0.10526	0.09517	**0.09364**
Mean ranks	combat	stand	ratioa	meanc	none	ratiog	**fabatch**	sva
	3.28571	3.57143	3.78571	4.14286	4.5	4.64286	**5.85714**	6.21429
skewdiv								
Mean values	**fabatch**	sva	stand	combat	ratioa	ratiog	meanc	none
	**0.01724**	0.02206	0.02377	0.02688	0.02875	0.03257	0.03671	0.05041
Mean ranks	sva	**fabatch**	combat	stand	meanc	ratioa	ratiog	none
	2.21429	**2.92857**	4.28571	4.78571	5.07143	5.42857	5.5	5.78571
corbeaf								
Mean values	none	combat	meanc	ratioa	ratiog	stand	sva	**fabatch**
	1	0.86857	0.86742	0.85516	0.84931	0.82754	0.69313	**0.67795**
Mean ranks	none	combat	meanc	ratiog	ratioa	stand	sva	**fabatch**
	1	2.92857	2.92857	4.21429	4.35714	5.85714	7.14286	**7.57143**

In each row the results are listed in descending order according to mean performance in terms of the original values and their ranks, respectively. The results of FAbatch are printed in bold

According to the values of the separation score (Additional file 1: Figure S8 and Fig. 2, Additional file 1: Table S1 and Table 2) ComBat, FAbatch and standardization seem to lead to the best mixing of the observations across the batches. For the real datasets, however, standardization was only slightly better on average than other methods.

The results with respect to avedist are less clear. The simulation with factors (Design A) suggests that FAbatch and SVA are associated with greater minimal distances to neighboring batches, compared to the other methods. However, we do not clearly observe this for Design B other than for the setting with common correlations. The real data results also suggest no clear ordering between the methods with respect to this metric; see in particular the means over the datasets in Table 2. The values of this metric were not appreciably improved by batch effect adjustment in general on the real datasets.

The values of klmetric, which is conceptionally very similar to the separation score, allows a very similar conclusion as the latter metric (Additional file 1: Figure S10 and Fig. 2, Additional file 1: Table S3 and Table 2): ComBat, FAbatch and standardization performed best here. While this conclusion could be obtained on both simulated and real data, other results differed between the different simulation scenarios and the real data analyses: SVA performed considerably worse here for Design A than B and mean-centering performed better on the simulated data in general.

The estimates of the proportions of the variation explained by the class signals obtained via Principal Variance Components Analysis (pvca) are depicted in the Additional file 1: Figure S11 and Fig. 2 and summarized in the Table S4 (Additional file 1) and Table 2. SVA appears to be associated with the highest proportion of variation induced by the class signal. However, the comparison to the other methods is not fair here: SVA makes use of the target variable and is therefore associated with an artificially increased class signal. See the Section “Artificial increase of measured class signal by applying SVA” for details on this mechanism related to overoptimism. FAbatch performed well only on the simulated data here, but not on the real datasets, where it had the lowest mean value with the exception of no batch effect adjustment. Figure 2 reveals that those three datasets for which pvca was considerably smaller after batch effect adjustment by FAbatch were, at the same time, the three datasets with the highest pvca-values before batch effect adjustment. Datasets with high pvca-values are datasets where the biological signal is relatively strong in comparison to the batch effects. Our results suggest that for such datasets, batch effect adjustment with FAbatch might be counterproductive. The distinguishing feature of FAbatch in comparison to a mere location-scale adjustment as performed by ComBat is that it aims at additionally adjusting for batch effects not explainable by location and scale shifts. While FAbatch aims at protecting the biological signal in the factor estimation, it cannot be protected entirely here due to the uncertainty in the estimation of the class probabilities. When reducing the total heterogeneity by FAbatch in cases of weak batch effects, the merit of removing heterogeneity due to batch effects becomes smaller in comparison to the harm that affects the signal. ComBat performed better than other methods here on the real data (with the exception of SVA as mentioned before).

For the performance metric related to differential expression analysis diffexpr (Additional file 1: Figure S12 and Fig. 2, Additional file 1: Table S5 and Table 3) the results for FAbatch and SVA are quite different between simulated and real data. In the simulation, the two methods performed best compared to the others (with the exception of FAbatch for Design B with common correlation). However, for the real data they performed worst—even worse than no batch effect adjustment in the mean. For FAbatch we examined those datasets which yielded substantially worse diffexpr-values after batch effect adjustment than before. As can already be seen from Fig. 2, two of these datasets have high diffexpr-values on the data before batch effect adjustment. This implies that for these datasets the biological signal is well preserved in the batches—in other words they seem to be less affected by batch effects. A possible reason why FAbatch performs worse for mild batch effects has already been outlined above. The other datasets connected with worse diffexpr-values than “no batch effect adjustment” in the case of FAbatch were those datasets for which some “outlying” batches were very different from the others—according to the PCA plots given in (Additional file 1: Figure S7). We conjecture that, in this case, pooling the data of the outlying batch(es) with the other batches and estimating the *L*_2_-penalized logistic regression model can result in a predictor with bad performance. The combined data might be too heterogeneous for the *L*_2_-penalized logistic regression model, which assumes that all observations follow the same distribution. If the predictions of the class probabilities by the *L*_2_-penalized logistic regression rule are bad, the biological signal is less protected in the latent factor estimation. Therefore, the removal of the estimated latent factor influences will affect the biological signal more. There were no noteworthy differences between the other methods with respect to diffexpr. For the real datasets there were also no improvements over no batch effect adjustment. This indicates that differential expression analysis might not benefit from batch effect adjustment in general.

For the skewness divergence score (Additional file 1: Figure S13 and Fig. 2, Additional file 1: Table S6 and Table 3) no clear ranking between the methods is seen in the case of the simulated data. However, for the real datasets, SVA and FAbatch clearly outperform the other methods with respect to this metric.

Finally, both in the simulated and real data, FAbatch and SVA have considerably lower corbeaf-values (Additional file 1: Figure S14 and Fig. 2, Additional file 1: Table S7 and Table 3), which is not very surprising considering their high complexity.

### Application in cross-batch prediction

In this illustrative analysis we apply all batch effect adjustment methods considered above together with the corresponding addon procedures described in the Section “Addon adjustment of independent batches” in cross-batch prediction in a real data example and using simulated data. A more extensive real data study was conducted by Luo et al. [3] who used several datasets to compare all of the methods considered here, except for frozen SVA (“fSVA”) and FAbatch, with respect to their performance in cross-batch prediction.

We use the dataset IUGRTranscr. The reasons for choosing this dataset were that it features a relatively strong class signal and is at the same time strongly affected by batch effects—judging from the principal component analysis plot in Additional file 1: Figure S7. This dataset contains miRNA-measurements obtained from 67 human placentas using the Illumina Human-6 v2 Expression BeadChip. Of these 67 samples, 27 were obtained from placentas of embryos suffering from intrauterine growth restriction (IUGR), the remaining 40 samples originate from placentas of healthy embryos. The dataset consists of two batches of sizes 20 and 47, where in the first batch 9 (45 %) and in the second batch 18 (≈38 *%*) samples originate from IUGR embryos.

As classification algorithm for the dependent variable “IUGR (yes vs. no)” Linear Discriminant Analysis (LDA) using Partial Least Squares (PLS) components as covariates [20] was chosen, where the number of components used was tuned on the grid 1,2, …, 10 employing 3-fold CV.

Just as Luo et al. [3] in their extensive real data study, we use Matthews Correlation Coefficient (MCC) as performance metric. This measure has the advantage over the more commonly considered misclassification error rate, that it is independent of the class frequencies in the test data. It takes values in [ −1,1], where a MCC-value of 1 would indicate a perfect prediction, a MCC-value of 0 would correspond to a completely random prediction and a MCC-value of -1 to a total disagreement between prediction and reality.

**Fig. 3:**
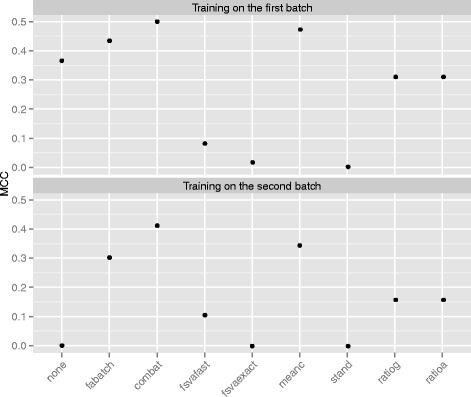
Cross-batch prediction—MCC-values. MCC-values out of using the individual batch effect adjustment methods in cross-batch prediction when training on the first and second batch. fsvafast and fsvaexact denote the fast and the exact fSVA algorithm, respectively

Figure 3 depicts the MCC-values resulting when applying the different batch effect adjustment methods in predicting from one batch to the other and than switching the training and test set roles between the two batches. When training on the first batch only ComBat, mean-centering and FAbatch lead to a higher MCC-value in comparison to no batch effect adjustment. The two fSVA algorithms and standardization lead to a very strong deterioration of the prediction performance, where the fast fSVA algorithm was slightly better than the exact fSVA algorithm. When training on the second batch, the prediction performance without batch effect adjustment corresponded to random guessing as indicated by the MCC-value of zero here. Except for standardization and the exact fSVA algorithm, all methods lead to a more or less strong improvement of prediction performance here. The ranking between the methods is almost entirely the same compared to that when training on the first batch.

**Fig. 4:**
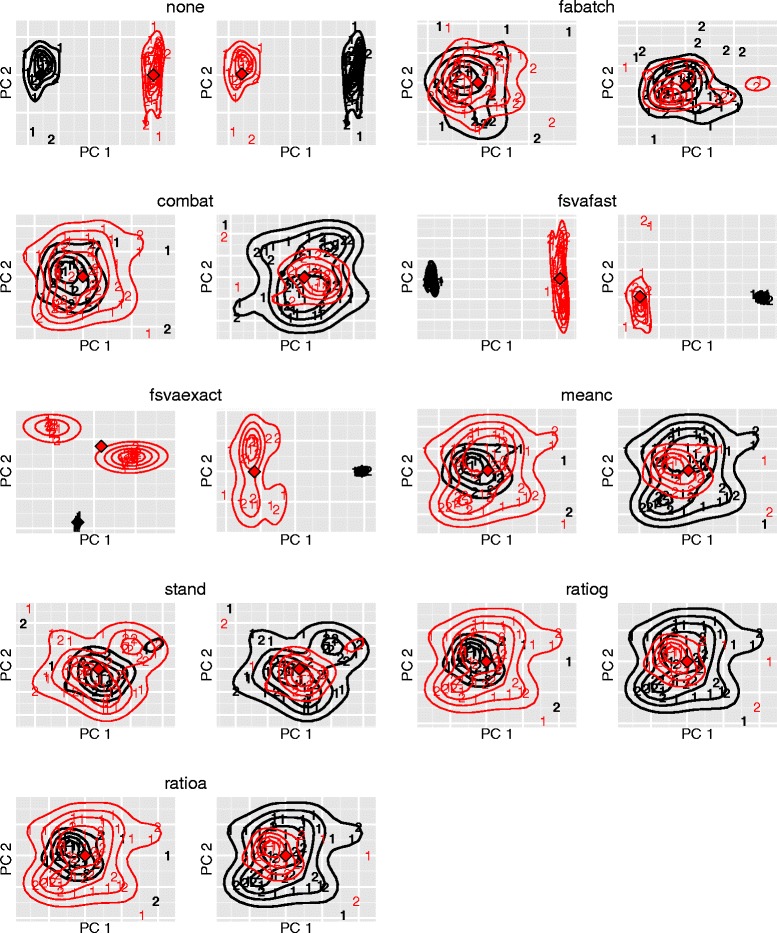
Visualization of the assimilation of validation batch to training batch after batch effect adjustment. First two principal components out of PCA performed on the following data matrix: the training batch after batch effect adjustment combined with the validation batch after addon batch effect adjustment. The training batch in each subplot is depicted in bold and the numbers distinguish the two classes “IUGR yes” (2) vs. “IUGR no” (1). The contour lines represent batch-wise two-dimensional kernel estimates and the diamonds represent the batch-wise centers of gravities of the points

In Additional file 1: Figure S7 and in Fig. 1 we used PCA plots to visualize batch effects in raw data and in data after batch effect adjustment, respectively. In this section we utilize such plots for a slightly different purpose: to study to what extent the validation batch is similar to the training batch after addon batch effect adjustment using the different batch effect adjustment methods. In each panel of Fig. 4 the training batch is depicted in bold. In each case PCA was applied to the following data matrix: the training batch after batch effect adjustment combined with the validation batch after addon batch effect adjustment using the respective method indicated in each case. The stronger the two point clouds overlap, the closer the validation batch is to the training batch after addon batch effect adjustment. Before batch effect adjustment the two batches are obviously grossly disparate. While the shapes of the point clouds are rather similar, their location differs strongly. FAbatch lead to the greatest overlap between the training and validation batches. ComBat and standardization were second place here. Note that despite the decent overlap between training and validation batches using standardization, this method delivered bad MCC-values in the analysis above. Mean-centering, ratio-A and ratio-G were connected with a worse overlap and the point clouds do hardly differ between these methods. The two fSVA algorithms made the two point clouds even more disparate than before batch effect adjustment. The bad performance of fSVA observed here indicates that in this example it seems not to be appropriate to assume that the same sources of heterogeneity operate in the two batches—an assumption required for the application of fSVA. In Section “Addon adjustment of independent batches” we noted that for the methods mean-centering, standardization, ratio-A and ratio-G no specific addon batch effect adjustment methods are required, because they treat each independently of the others. Therefore, for each of these methods, in the two corresponding subplots of Fig. 4 the point clouds are identical, irrespective of which batch is used as training and validation batch, respectively.

**Fig. 5:**
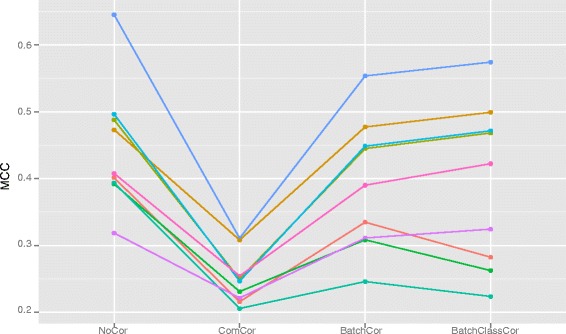
MCC-values from simulation study. The colors differentiate the methods: none (), fabatch (), combat (), fsvafast (), fsvaexact (), meanc (), stand (), ratiog (), ratioa (). For better interpretability the results to the same methods are connected

Note again that the above real data analysis is only illustrative. Simulations give more accurate results and allow for the study of the impact of specific aspects of the underlying data distribution. In this simulation we are interested in demonstrating that FAbatch is best suited in situations with correlated predictors. We considered four simulation settings. These are: the three settings of Design B presented in Section “Design B: Drawing from multivariate distributions with specified correlation matrices” and an additional setting in which no correlation between the predictors was induced. Design B was chosen instead of Design A in order to prevent a possible optimistic bias with respect to FAbatch and fSVA, since these involve adjustment for latent factor influences. The additional fourth setting was generated by simply setting the correlations in Design B to zero. For each setting we simulated 100 datasets and proceeded as in the analysis of the real dataset presented above—with two differences. The first difference was that in the simulation we have to consider ${4 \choose 2} \times 2 = 12$ instead of two combinations of training and validation batches per dataset, because the simulated datasets feature four instead of only two batches. The second difference concerns the evaluation of the results, because the MCC values could not be calculated in cases where both the numerator and denominator in the calculation were zero. Therefore for each combination of setting and batch effect adjustment method we summed up the true positives, the true negatives, the false positives and the false negatives over all prediction iterations in all 100 datasets and calculated the MCC-value using the standard formula. Figure 5 shows the results. In many respects the simulation results concur with the results obtained using the real dataset. The most striking difference is that standardization was best here, while it was bad for the real data analysis. The good performance of standardization in the simulation should however not be over-interpreted as it was the least performant method in the study of Luo et al. [3]. FAbatch was the second-best method in all settings except for that without correlation between the predictors. In the latter setting, FAbatch is outperformed by ComBat and mean-centering. This confirms that FAbatch is best suited in situations with more correlated variables. Ratio-G performed poorly here—other than in the study by Luo et al. [3] and in the real-data analysis above. Both frozen SVA algorithms performed bad here as well.

### Artificial increase of measured class signal by applying SVA

In the Section “FAbatch” we detailed why using the actual values of the target variable in protecting the biological signal during the latent factor estimation of FAbatch would lead to an artificially increased class signal. SVA does use the values of the target variable and indeed suffers from the problem of an artificially increased class signal. In the following, we will outline the reason why SVA suffers from this problem. A crucial problem with the weighting of the variable values by the estimated probabilities that the corresponding variable is associated with unmeasured confounders but not with the target variable is the following: these estimated probabilities depend on the values of the target variable, in particular for smaller datasets. Naturally, due to the variability in the data, for some variables the measurements are, by chance, separated overly strong between the two classes. Such variables, for which the observed separation between the classes is larger than the actual—biologically motivated—separation, are connected with smaller estimated weights. This means that such variables are affected less strongly by the removal of the estimated latent factor influences compared to variables which are not connected with such a randomly increased separation. Phrased differently, the stronger the apparent—not the actual—signal of a variable is, the less its values are affected by the adjustment of latent factors. As a result, after applying SVA the classes are separated to a stronger degree than they would be if biological differences between the classes were the only source of separation—as is required in a meaningful analysis. This phenomenon is pronounced more strongly in smaller datasets. The reason for this is that for larger datasets the measured signals of the variables get closer to the actual signals, wherefore the overoptimism due to working with the apparent instead of the actual signals becomes less pronounced here. Accordingly, in the real data example from the previous subsection fSVA performed considerably worse when using the smaller batch as training data.

Using datasets with artificially increased signals in analyses can lead to over-optimistic results, which can have dangerous consequences. For example, when the result of cross-validation is over-optimistic, this may lead to overestimating the discriminatory power of a poor prediction rule. Another example is searching for differentially expressed genes. Here, an artificially increased class signal could lead to an abundance of false-positive results.

The observed deterioration of the MCC-values in the real data example by performing frozen SVA when training on the smaller batch may, admittedly, also be due to random error. In order to investigate whether the effects originating from the mechanism of artificially increasing the discriminative power of datasets by performing SVA are strong enough to have actual implications in data analysis, we performed a small simulation study. We generated datasets with 40 observations, 1000 variables, two equally sized batches, standard normally distributed variable values and a binary target variable with equal class probabilities. Note that there is no class signal in this data. Then using 5-fold cross-validation repeated two times we estimated the misclassification error rate of PLS followed by LDA for this data. Consecutively, we applied SVA to this data and again estimated the misclassification error rate of PLS followed by LDA using the same procedure. We repeated this procedure for the number of factors to estimate set to 1, 2 and 3, respectively. In each case we simulated 50 datasets. The mean of the misclassification error rates was 0.504 for the raw datasets and 0.431, 0.356 and 0.306 after applying SVA with 1, 2 and 3 factors. These results confirm that the artificial increase of the class signal by performing SVA can be strong enough to have implications in data analysis. Moreover, the problem seems to be more severe for a higher number of factors estimated. We did the same analysis with FAbatch, again using 1, 2 and 3 factors, where we obtained the misclassification error rates 0.505, 0.521 and 0.509, respectively, suggesting that FAbatch does not suffer from this problem in the investigated context.

## Discussion

In this paper, with FAbatch, we introduced a very general batch effect adjustment method for situations in which the batch membership is known. It accounts for two kinds of batch effects simultaneously: 1) coarse, easily observable batch effects expressed as location and scale shifts of the variable values across the different batches; 2) more complicated batch effects, modelled by latent factor influences, which affect the correlations between the variables in the batches. The model behind FAbatch is an extension of the model underlying ComBat, where the latter is designed to address the first kind of the batch effects described above. FAbatch uses latent factors to model batch effects in the spirit of SVA. In contrast to SVA, however, FAbatch assumes that the batch membership of the observations is known and that the latent factor models are batch-specific, i.e. that in each batch different sources of heterogeneity may operate. In Appendix A.2 (Additional file 1) it is shown that in the SVA model it is implicitly assumed that the distribution of the vector of latent factors may be different for each observation. This is a very general assumption. However, it is unclear how well SVA can deal with specific datasets originating from such a general model, because the link between the singular value decomposition used in the estimation and this model is not evident. Our algorithm by contrast was explicitly motivated by its underlying model, which is quite general and reasonable. In cases in which the data in question is approximately uniform with this model, FAbatch should perform reasonably well. In the form presented here, FAbatch is only applicable in the presence of a binary target variable. However, it can also be extended to other types of target variables. For example, when having a metric target variable one could use ridge regression instead of *L*_2_-penalized logistic regression when protecting the biological signal of interest in the factor estimation.

In an illustrative analysis we applied the batch effect adjustment methods studied in the main analyses in the important case of cross-batch prediction. FAbatch—other than fSVA—performed reasonably well in this example. Moreover, by a small simulation study we obtained evidence that the artificial increase of the measured biological signal of interest faced when performing SVA can have noticeable negative effects in applications. In FAbatch, this artificial increase is prevented by employing the following idea: for each observation the parameters involved in the transformations performed for protecting the biological signal are estimated using training data, which does not contain the respective observation to be transformed. This idea may also be applied in the protection of the biological signal of SVA, i.e. when multiplying the variable values by the estimated probabilities that the corresponding variables are associated with unmeasured confounders, but not with the binary variable representing the biological signal. More precisely these probabilities could be estimated in a cross-validation procedure—taking up again the idea also used in FAbatch.

All batch effect adjustment methods considered in this paper, together with the corresponding addon procedures and all metrics used in the comparisons of the methods, were implemented/adopted into the new R package bapred available online from CRAN [21].

## Conclusions

FAbatch leads to a good mixing of the observations across the batches in comparison to other methods, which is reassuring given the diversity of batch effect structures in real datasets. In the case of very weak batch effects and in the case of strongly outlying batches, the observed biological signal may be slightly altered by FAbatch. In our extensive comparison study of existing and new batch effect correction methods, we found that no method was best with respect to all metrics. It is thus difficult to formulate general recommendations: the choice of the method may primarily depend on the goal of the researcher as reflected by the choice of the metric. Performing no batch effect correction at all is in any case not recommended.

## Additional files

Additional file 1
**This file contains all Supporting Information referenced in the paper.** A Description of existing batch effect adjustment methods. B Plots used in verification of model assumptions. C Visualizations of the batch effects in the used datasets. D Target variables of datasets used in comparison study. E Reasons for batch effect structures of datasets used in comparison study. F Boxplots of the metric values for simulated datasets per method and simulation scenario. G Tables of the means of the metric values and their ranks for simulated datasets per method and scenario. (PDF 1707 kb)

Additional file 2
**This folder contains all necessary R-Code to reproduce and evaluate the real-data analyses and simulations, as well as Rda-files enabling fast evaluation of the corresponding results.** (ZIP 2406 kb)

## Abbreviations

avedist: average minimal distance to other batch
BatchClassCor: batch- and class-specific correlation structures
BatchCor: batch-specific correlation structures
ComCor: common correlation structure in all batches
corbeaf: mean Pearson’
s correlation of the variable values before and after batch effect adjustment; diffexpr: performance of differential expression analysis
fSVA: frozen SVA
fsvaexact: exact fSVA algorithm
fsvafast: fast fSVA algorithm
klmetr: Kullback-Leibler divergence between density of within and between batch pairwise distances
pvca: proportion of variation induced by class signal estimated by Principal Variance Components Analysis
sepscore: separation score
skewdiv: skewness divergence score
