# Supplementary material for: Combining location-and-scale batch effect adjustment with data cleaning by latent factor adjustment
Source: BMC Bioinformatics. 2016 Jan 12;17:27. doi: 10.1186/s12859-015-0870-z (PMC4710051; doi:10.1186/s12859-015-0870-z)
Supplement: Additional file 1 — This file contains all Supporting Information referenced in the paper. A Description of existing batch effect adjustment methods. B Plots used in verification of model assumptions. C Visualizations of the batch effects in the used datasets. D Target variables of datasets used in comparison study. E Reasons for batch effect structures of datasets used in comparison study. F Boxplots of the metric values for simulated datasets per method and simulation scenario. G Tables of the means of the metric values and their ranks for simulated datasets per method and scenario. (PDF 1707 kb) [file 12859_2015_870_MOESM1_ESM.pdf]

Supporting information for the BMC Bioinformatics article:

Combining location-and-scale batch effect adjustment with data cleaning by latent factor adjustment

Roman Hornung*, Anne-Laure Boulesteix, David Causeur

*Corresponding author: e-mail: hornung@ibe.med.uni-muenchen.de, Phone: +4989-440077754, Fax: +4989-440077491, Department of Medical Informatics, Biometry and Epidemiology, University of Munich, Marchioninistr. 15, D-81377, Munich, Germany

A Description of existing batch effect adjustment methods

A.1 ComBat

This method assumes the following model for the observed data x_{ijg}:

$$x_{ijg} = \alpha_g + \boldsymbol{a}_{ij}^T \boldsymbol{\beta}_g + \gamma_{jg} + \delta_{jg}\epsilon_{ijg}, \quad \epsilon_{ijg} \sim N(0, \sigma_g^2).$$

Here all involved parameters are as in the Section "Methods" of the main paper. Note that the restriction to binary target variables, which is not necessary in general for the application of ComBat, is required for the application of our method.

The unobserved counterpart x_{ijg}^* of x_{ijg} not affected by batch effects is assumed to be

$$x_{ijg}^* = \alpha_g + \boldsymbol{a}_{ij}^T \boldsymbol{\beta}_g + \epsilon_{ijg}, \quad \epsilon_{ijg} \sim N(0, \sigma_g^2).$$

The goal of batch effect correction via ComBat is to estimate these unobserved x_{ijg}^*-values. The following transformation of the observed x_{ijg}-values would provide the true x_{ijg}^*-values:

$$\begin{aligned}
&\sqrt{\mathrm{Var}(x_{ijg}^*)}\left(\frac{x_{ijg} - \mathrm{E}(x_{ijg})}{\sqrt{\mathrm{Var}(x_{ijg})}}\right) + \mathrm{E}(x_{ijg}^*) \\
&= \sigma_g\left(\frac{x_{ijg} - (\alpha_g + \boldsymbol{a}_{ij}^T\boldsymbol{\beta}_g + \gamma_{jg})}{\delta_{jg}\sigma_g}\right) + \alpha_g + \boldsymbol{a}_{ij}^T\boldsymbol{\beta}_g \qquad (1) \\
&= \alpha_g + \boldsymbol{a}_{ij}^T\boldsymbol{\beta}_g + \epsilon_{ijg} = x_{ijg}^*.
\end{aligned}$$

In practice, however, the parameters involved in Eq. (1) are not known and have to be estimated. In particular, γ_{jg}/σ_g and δ_{jg} are estimated using empirical Bayes to obtain more robust results. See [1] for details on the estimation procedure. Note that in the analyses performed in the main paper we do not include the term $\boldsymbol{a}_{ij}^T\boldsymbol{\beta}_g$ in the adjustment. The first reason for this is that in the Section "Application in cross-batch prediction" of the main paper we study cross-batch prediction. Here the class values $\boldsymbol{a}_{ij}$ are not known in the test data. The second reason is that using the class values $\boldsymbol{a}_{ij}$ together with the estimates of $\boldsymbol{\beta}_g$ may lead to an artificially increased class signal, because the estimates of $\boldsymbol{\beta}_g$ depend on the class values $\boldsymbol{a}_{ij}$. This kind of mechanism is discussed in detail, but in slightly other contexts, in the Sections "Using estimated probabilities instead of actual classes" and "Artificial increase of measured class signal by applying SVA" of the main paper.

A.2 SVA

The model for the observed data is given by:

$$\begin{aligned}
x_{ijg} &= \alpha_g + \boldsymbol{a}_{ij}^T\boldsymbol{\beta}_g + \sum_{l=1}^{m} b_{gl} Z_{ijl} + \epsilon_{ijg}, \qquad (2) \\
\mathrm{Var}(\epsilon_{ijg}) &= \sigma_g^2.
\end{aligned}$$

Here α_g and $\boldsymbol{a}_{ij}^T\boldsymbol{\beta}_g$ are as in the previous subsection and $Z_{ij1}, \dots, Z_{ijm}$ are random latent factors with loadings $b_{g1}, \dots, b_{gm}$.

The unobserved, batch-free data is correspondingly:

$$x_{ijg}^* = \alpha_g + \boldsymbol{a}_{ij}^T\boldsymbol{\beta}_g + \epsilon_{ijg}, \quad \mathrm{Var}(\epsilon_{ijg}) = \sigma_g^2. \tag{3}$$

Note again that in the SVA-model the batch membership is assumed to be unknown. For judging the appropriateness of the SVA algorithm it is important to specify the model underlying SVA as precisely as possible. Out of the following two facts it can be followed that the distribution of the latent factors can be different for each observation—in the extreme case. Firstly, the assumed form of the batch-free data in Eq. (3) implies that the distortions between the batches are induced fully by the latent factors. Secondly, each observation may come from a different batch with own mean-, covariance- and correlation-structure.

The SVA batch effect adjustment is performed by substracting $\sum_{l=1}^m b_{gl}Z_{ijl}$ from x_{ijg}:

$$x_{ijg} - \sum_{l=1}^m b_{gl}Z_{ijl} = \alpha_g + \boldsymbol{a}_{ij}^T\boldsymbol{\beta}_g + \epsilon_{ijg} = x_{ijg}^*.$$

The latent factors are estimated as the first m right singular vectors from a singular value decomposition (SVD). In the Section "Background" of the main paper we stressed that inhomogeneities in datasets are not only due to batch effects, but also due to the biological signal of interest, i.e. the term $\boldsymbol{a}_{ij}^T\boldsymbol{\beta}_g$ in Eq. (2) and (3). Therefore, we noted that the biological signal of interest has to be protected during factor estimation in FAbatch. In SVA, to protect the biological signal, before performing the SVD on the transposed covariate matrix, the variable values are weighted by the estimated probabilities that the corresponding variables are associated with unmeasured confounders, but not with the binary variable representing the biological signal. The factor loadings are estimated by linear models. The "frozen SVA" procedure [2] is an extension of SVA [3], which we will detail in the following subsection.

A.2.1 frozen SVA (fSVA)

In addition to describing the two algorithms gathered under the designation "frozen SVA", in this subsection we demonstrate that the "fast fSVA algorithm" is the addon procedure for SVA in the vein of the Section "Addon adjustment of independent batches" of the main paper.

The b_{gl}- and the $\boldsymbol{\beta}_g$-values are two of the batch-unspecific parameters involved in the SVA adjustment. The $\boldsymbol{\beta}_g$-values are implicitly involved, namely when multiplying the variable values by the estimated probabilities that the corresponding variable is associated with unmeasured confounders, but not with the binary variable representing the biological signal. In both frozen SVA algorithms, when adjusting for batch effects in new observations the estimates of the b_{gl}-values obtained on the training data are used. Also, for multiplying the variable values of a new observation by the estimated probabilities that the corresponding variable is associated with unmeasured confounders but not with the target variable, both algorithms use the estimates obtained on the training data. The distinguishing feature between the two algorithms is the way estimates of the factors Z_{ijl} for new observations are obtained.

In the first frozen SVA algorithm, denoted as "exact fSVA algorithm" in [2], the latent factor vector for a new observation is estimated in the following way: 1) Combine the training data with the values of the new observation and multiply by the probabilities estimated on the training data; 2) Re-perform the SVD on the combined data from 1) and use the right singular vector corresponding to the new observation as the estimate of its vector of latent factors. This algorithm is, however, not an addon procedure. In this algorithm, the estimate of the latent factor vector for the test observation originates from a different SVD than the estimated latent factors of the training observations. Therefore, this new estimated latent factor behaves—at least to some extent—differently than that of the training data. As a consequence, when adjusting the new observation a feature of addon procedures is not given: the same kind of transformation must be performed for independent batches. This problem can be assumed to have a lower impact for larger training datasets. Here the latent factor model estimated on the training data depends less on whether a single new observation is included into the SVD or not. A solution to the problem of differently behaving latent factor estimates in training and test data would be the following: for adjusting the training data use the estimates of the latent factors (and their loadings) obtained in the second SVD performed after including the test observation. This would, however, again not correspond to an addon procedure, because then the adjusted training data would be changed each time a new observation is included, which is not allowed as stated in our definition of addon procedures given in the Section "Addon adjustment of independent batches" of the main paper.

The second frozen SVA algorithm, denoted as "fast fSVA algorithm" in [2] takes a different approach. Here, the SVD is not re-performed entirely on the combination of the training data and the new observation. Instead, one essentially performs a SVD for calculating the right singular vector corresponding to the new observation, in which the left singular vectors and singular values are fixed to the values of these parameters obtained in the SVA, which had been performed on the training data. Thus in this adjustment, it is taken into account that the left singular vectors and singular values are batch-unspecific parameters. The resulting estimated latent factor vector of the new observation behaves in the same way as that of the training data, because here it originates from the same SVD. This algorithm does correspond to an addon procedure, because the same kind of transformation is performed for independent batches, i.e. observations in the SVA model, without the need to change the training data.

A.3 Further batch effect adjustment methods considered in the comparison studies

A.3.1 Mean-centering

From each measurement the mean of the values of the corresponding variable in the corresponding batch is substracted:

$$\widehat{x_{ijg}^{*}} = x_{ijg} - \widehat{\mu_{jg}}, \tag{4}$$

where $\widehat{\mu_{jg}} = (1/n_j)\sum_j x_{ijg}$.

A.3.2 Standardization

The values of each variable are centered and scaled per batch:

$$\widehat{x^*_{ijg}} = \frac{x_{ijg} - \widehat{\mu_{jg}}}{\sqrt{\widehat{\sigma^2_{jg}}}},$$

where $\widehat{\mu_{jg}}$ as in (4) and $\widehat{\sigma^2_{jg}} = [1/(n_j - 1)] \sum_i (x_{ijg} - \widehat{\mu_{jg}})^2$.

A.3.3 Ratio-A

Each measurement is divided by the arithmetic mean of the values of the variable in the corresponding batch [4]:

$$\widehat{x^*_{ijg}} = \frac{x_{ijg}}{\widehat{\mu_{jg}}},$$

where $\widehat{\mu_{jg}}$ is that same as in (4).

A.3.4 Ratio-G

Each measurement is divided by the geometric mean of the values of the variable in the corresponding batch [4]:

$$\widehat{x^*_{ijg}} = \frac{x_{ijg}}{\widehat{\mu_{g,geom}}},$$

where $\widehat{\mu_{g,geom}} = \sqrt[n_j]{\prod_i^{n_j} x_{ijg}}$.

B Plots used in verification of model assumptions

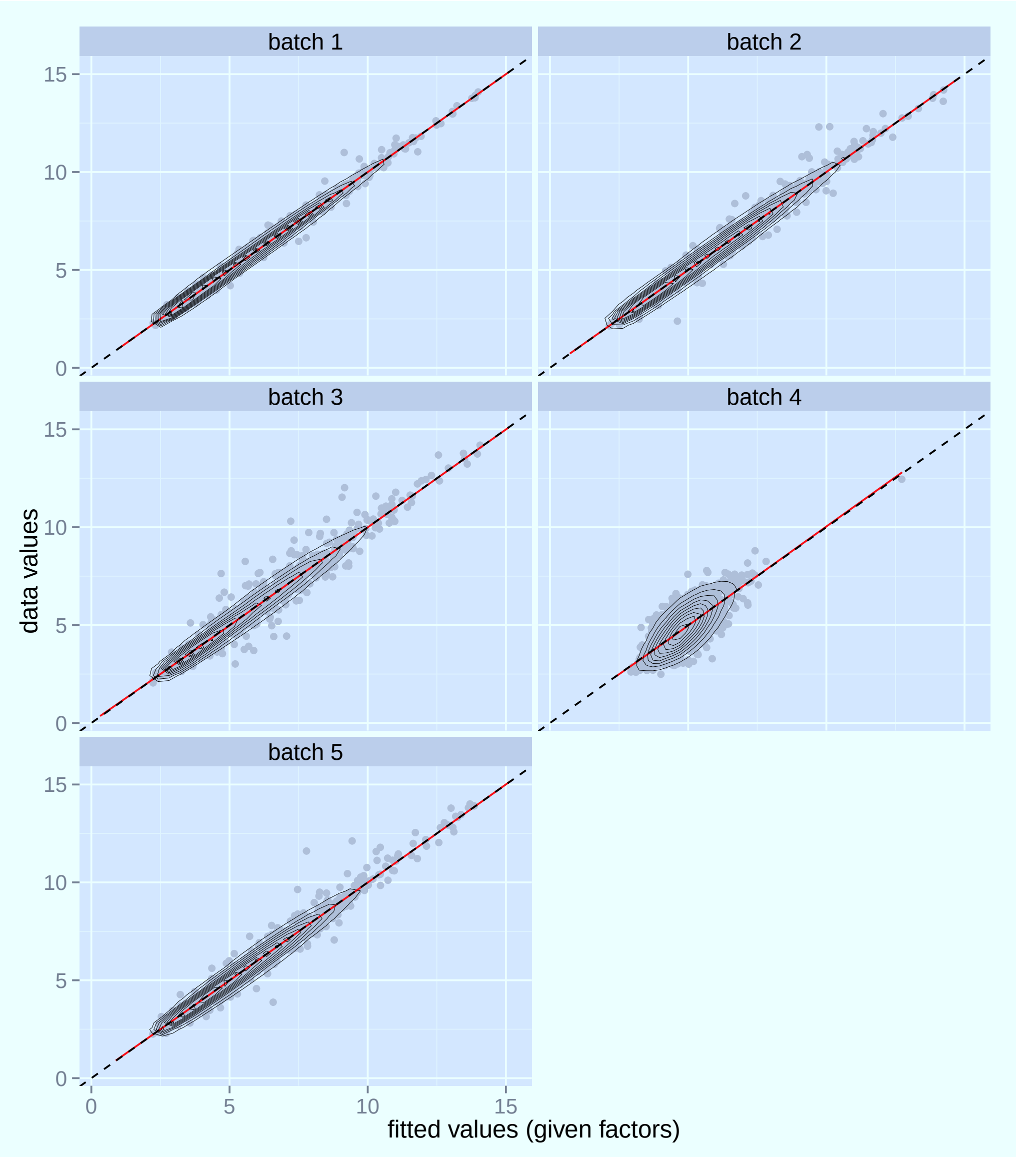

Fig. S1: Data values against fitted values resulting from FAbatch method. The contour lines represent two-dimensional kernel density estimates. The dashed line mark the bisectors and the red lines are LOESS estimates of the associations. The grey dots are in each case random subsets of size 1000 of all values.

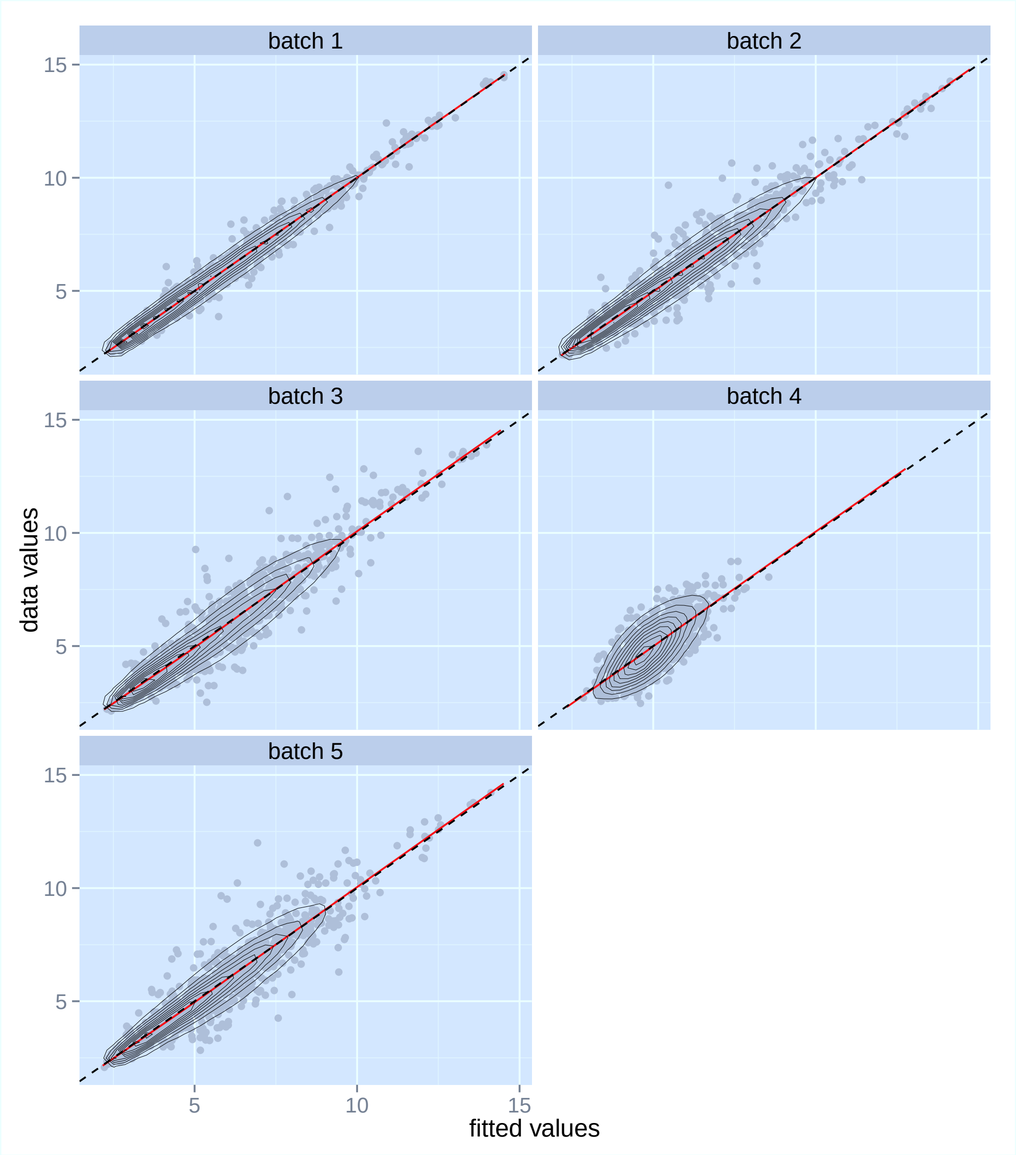

Fig. S2: Data values against fitted values resulting from ComBat method. The contour lines represent two-dimensional kernel density estimates. The dashed line mark the bisectors and the red lines are LOESS estimates of the associations. The grey dots are in each case random subsets of size 1000 of all values.

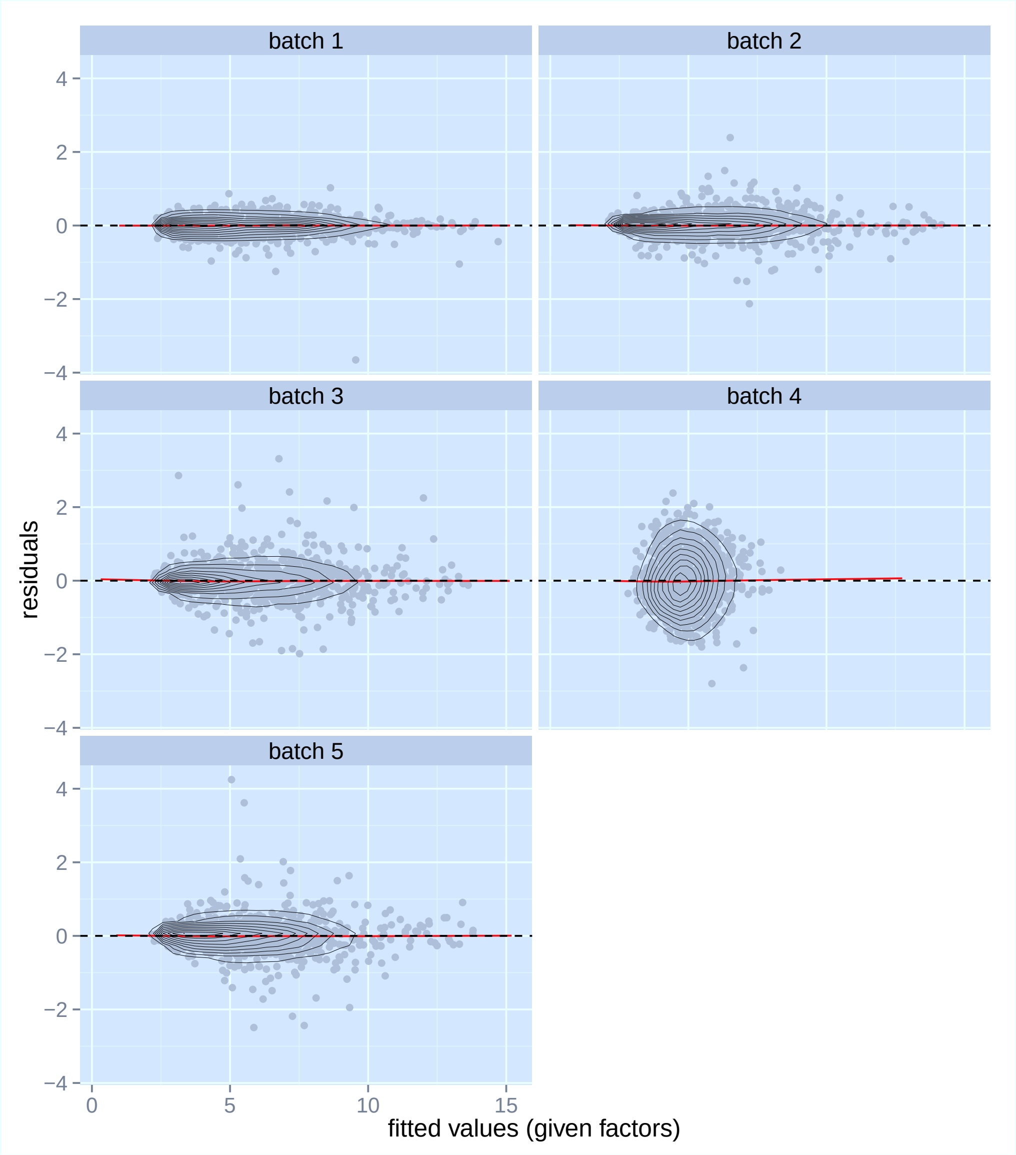

Fig. S3: Deviations from fitted values resulting from FAbatch method against corresponding fitted values. The contour lines represent two-dimensional kernel density estimates. The dashed lines mark the horizontal zero lines and the red lines are LOESS estimates of the associations. The grey dots are in each case random subsets of size 1000 of all values.

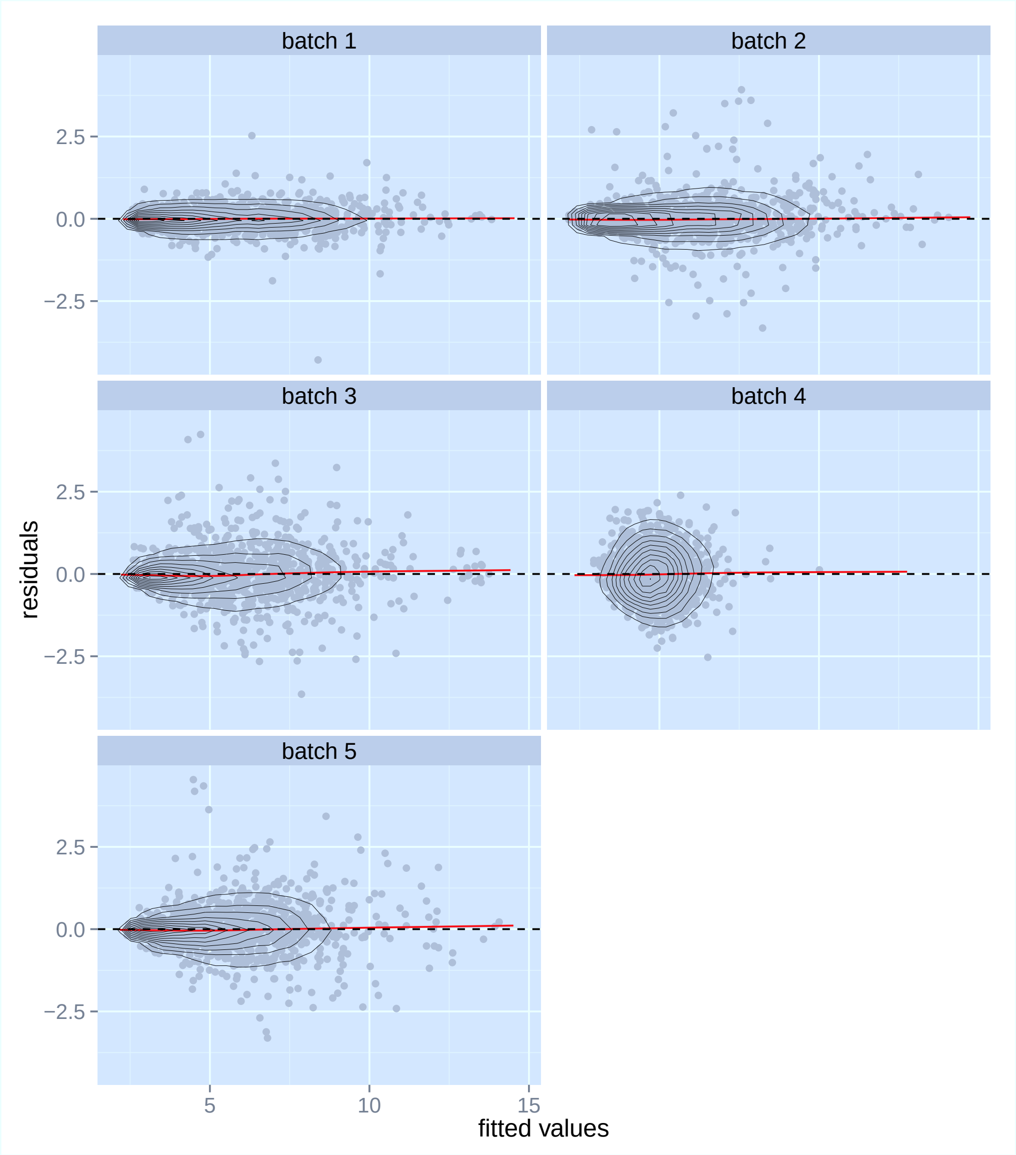

Fig. S4: Deviations from fitted values resulting from ComBat method against corresponding fitted values. The contour lines represent two-dimensional kernel density estimates. The dashed lines mark the horizontal lines and the red lines are LOESS estimates of the associations. The grey dots are in each case random subsets of size 1000 of all values.

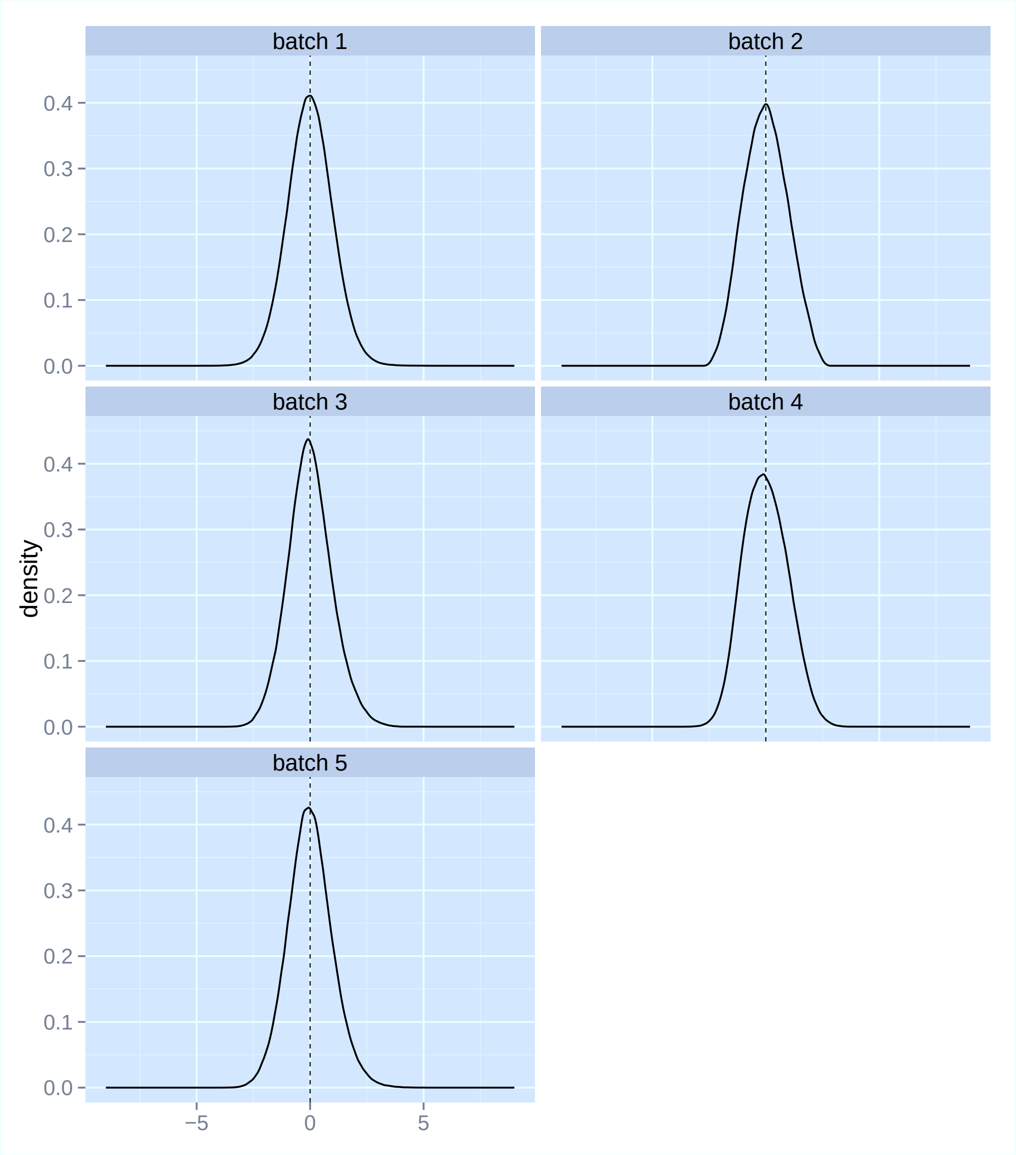

Fig. S5: Density estimates of the deviations from the fitted values divided by their standard deviations for the FAbatch method. The dashed lines mark the vertical zero lines.

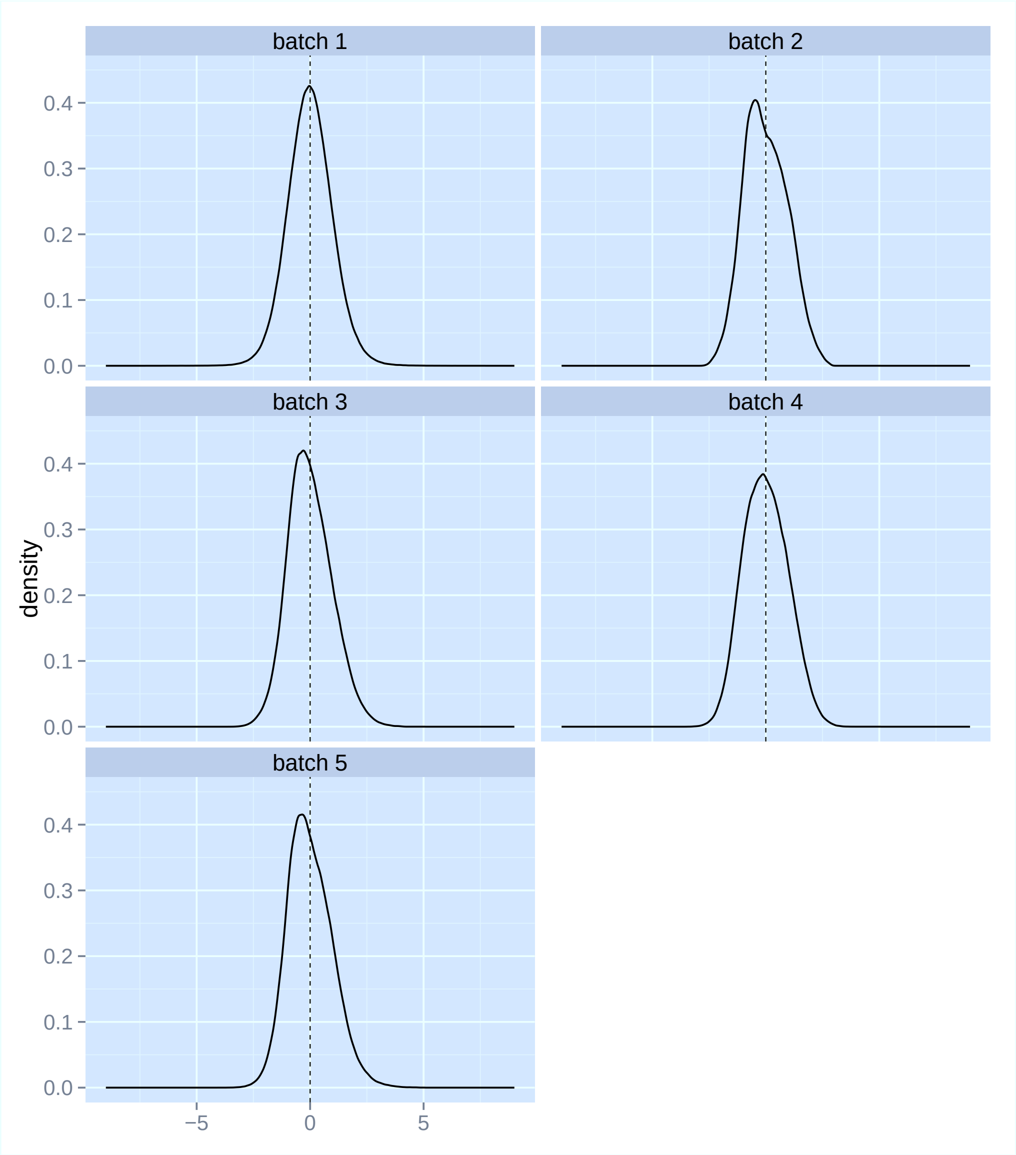

Fig. S6: Density estimates of the deviations from the fitted values divided by their standard deviations for the ComBat method. The dashed lines mark the vertical zero lines.

C Visualizations of the batch effects in the used datasets: plots of the first two principal components out of Principal Component Analysis

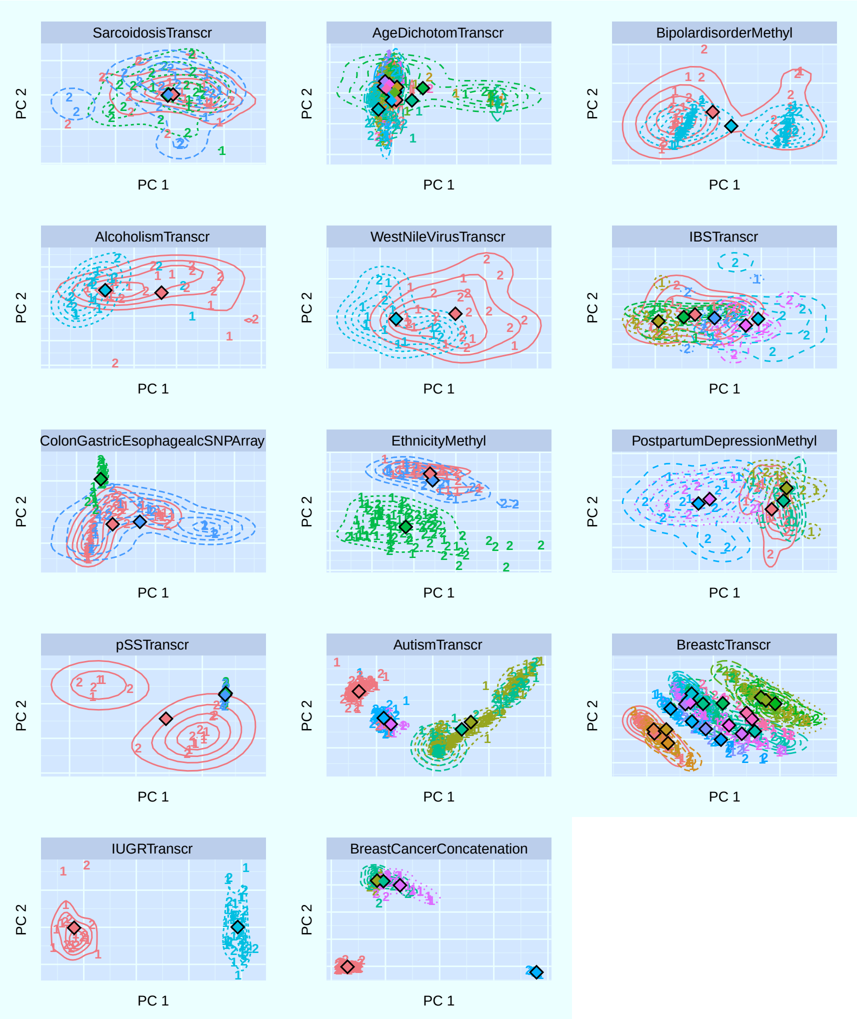

Fig. S7: Each subplot shows the first two principal components out of PCA performed on the covariate matrix of one of the datasets used. In each case the colors distinguish the batches, and the numbers distinguish the two classes "diseased" (2) vs. "healthy control" (1). The contour lines represent batch-wise two-dimensional kernel estimates and the diamonds represent the batch-wise centers of gravities of the points. The plots are arranged in ascending order according to the strength of batch effects with respect to the following criterion: Average over the euclidean distances between all possible pairs of points in the plot from different batches divided by the analoguous mean over all such pairs from the same batches.

D Target variables of datasets used in comparison study

ColonGastricEsophagealcSNPArray: “gastric cancer” ($y = 2$) vs. “healthy” ($y = 1$)

AgeDichotomTranscr: “chronic alcoholic” ($y = 2$) vs. “healthy” ($y = 1$)

EthnicityMethyl: “Caucasian, from Utah and of European ancestry” ($y = 2$) vs. “Yorubian, from Ibadan Nigeria” ($y = 1$)

BipolardisorderMethyl: “bipolar disorder” ($y = 2$) vs. “healthy” ($y = 1$)

PostpartumDepressionMethyl: “depression post partum” ($y = 2$) vs. “healthy” ($y = 1$)

AutismTranscr: “autistic” ($y = 2$) vs. “healthy” ($y = 1$)

BreastcTranscr: “breast cancer” ($y = 2$) vs. “healthy” ($y = 1$)

BreastCancerConcatenation: “breast cancer” ($y = 2$) vs. “healthy” ($y = 1$)

IUGRTranscr: “intrauterine growth restriction” ($y = 2$) vs. “healthy” ($y = 1$)

IBSTranscr: “constipation-predominant/diarrhoea-predominant irritable bowel syndrome” ($y = 2$) vs. “healthy” ($y = 1$)

SarcoidosisTranscr: “sarcoidosis” ($y = 2$) vs. “healthy” ($y = 1$)

pSSTranscr: “Sjogren’s/sicca syndrome” ($y = 2$) vs. “healthy” ($y = 1$)

AlcoholismTranscr: “alcoholic” ($y = 2$) vs. “healthy” ($y = 1$)

WestNileVirusTranscr: “severe West Nile virus infection” ($y = 2$) vs. “asymptomatic West Nile virus infection” ($y = 1$)

E Reasons for batch effect structures of datasets used in comparison study

EthnicityMethyl: "To limit the potential bias due to experimental batches, samples were randomized by population identity and hybridized in three batches." [5]

BreastcTranscr: "To minimize possible processing and chip lot effects, samples were assigned to processing batches of seven to nine pairs, and batches had similar distributions of age, race, and date of enrollment. For array hybridization, each batch was assigned to one of two different chip lots ('A' and 'B') in a manner designed to ensure a balance of these same characteristics. [...] Laboratory personnel were blind to case control status and other phenotype information." [6]

BreastCancerConcatenation: Concatenation of five independent datasets.

IUGRTranscr: Citation from the description on the ArrayExpress-website: "[...] were collected during the years of 2004-2008 and hybridized in two batches to microarrays. Samples were randomized across arrays to control for array and batch variability."

AlcoholismTranscr: The batch variable in the sdrf.txt-file is designated as "labeling batch", from which we deduced that the batch structure is due to labeling for this dataset.

F Boxplots of the metric values for simulated datasets per method and simulation scenario

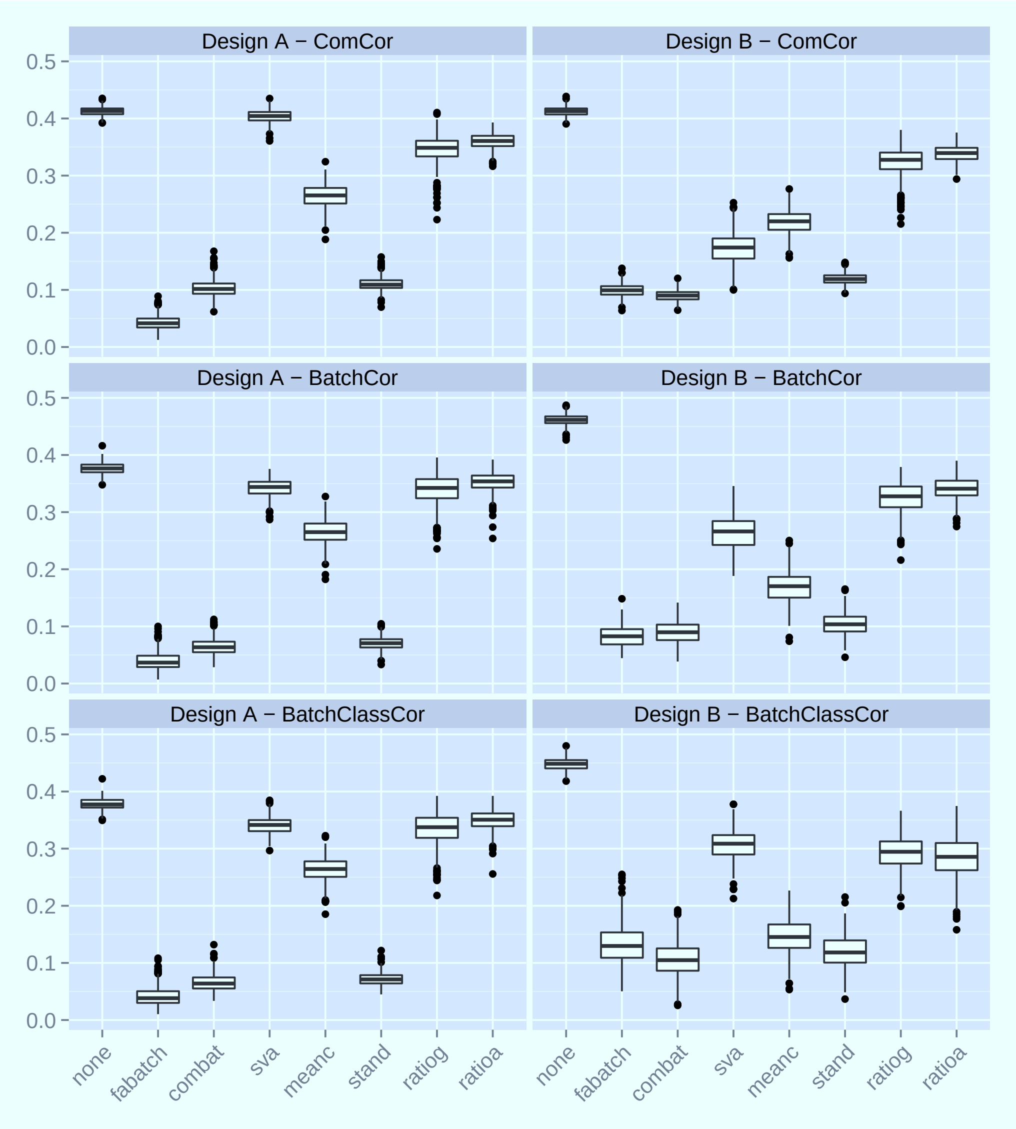

Fig. S8: Values of metric `sepscore` for all simulated datasets separated into simulation scenario and method.

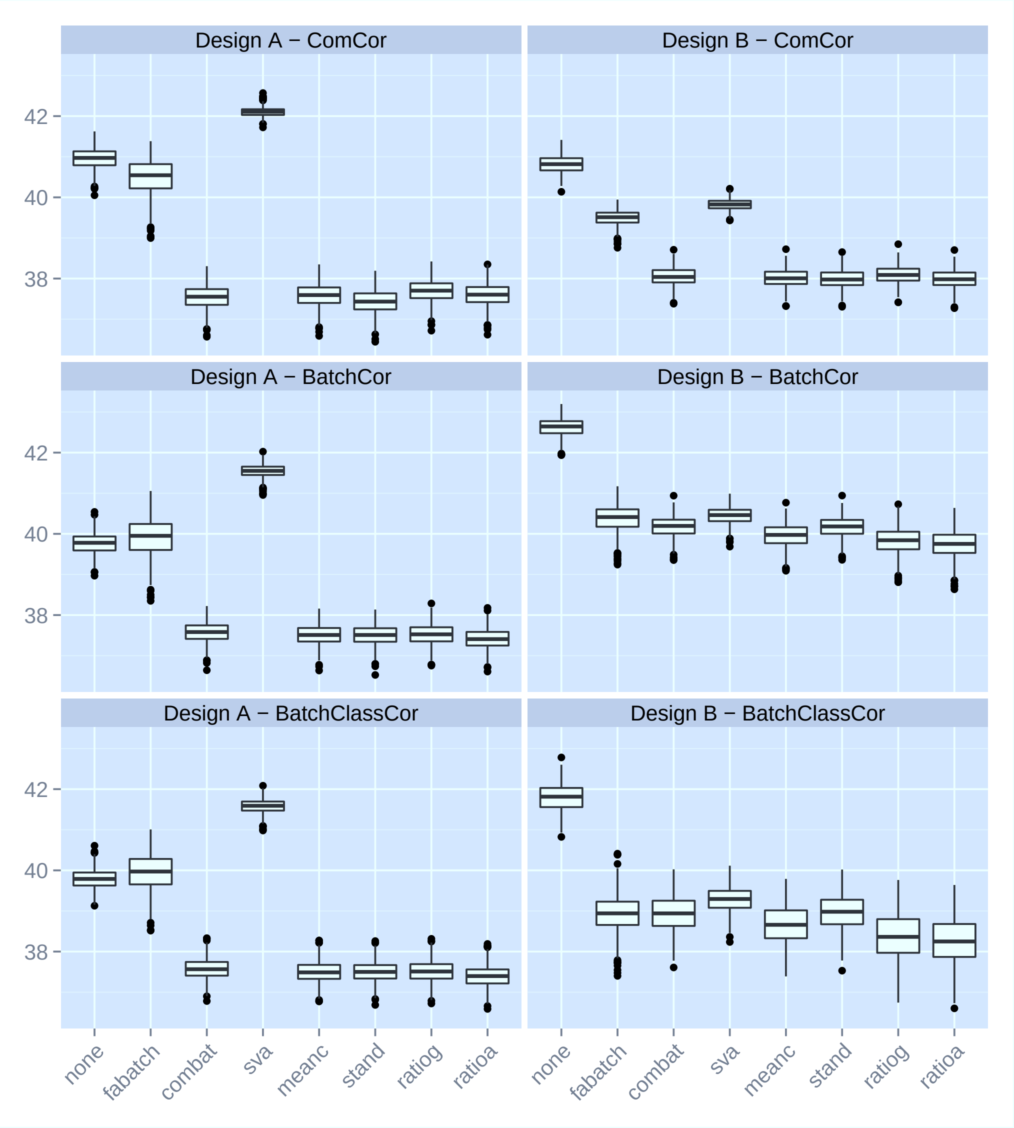

Fig. S9: Values of metric `avedist` for all simulated datasets separated into simulation scenario and method.

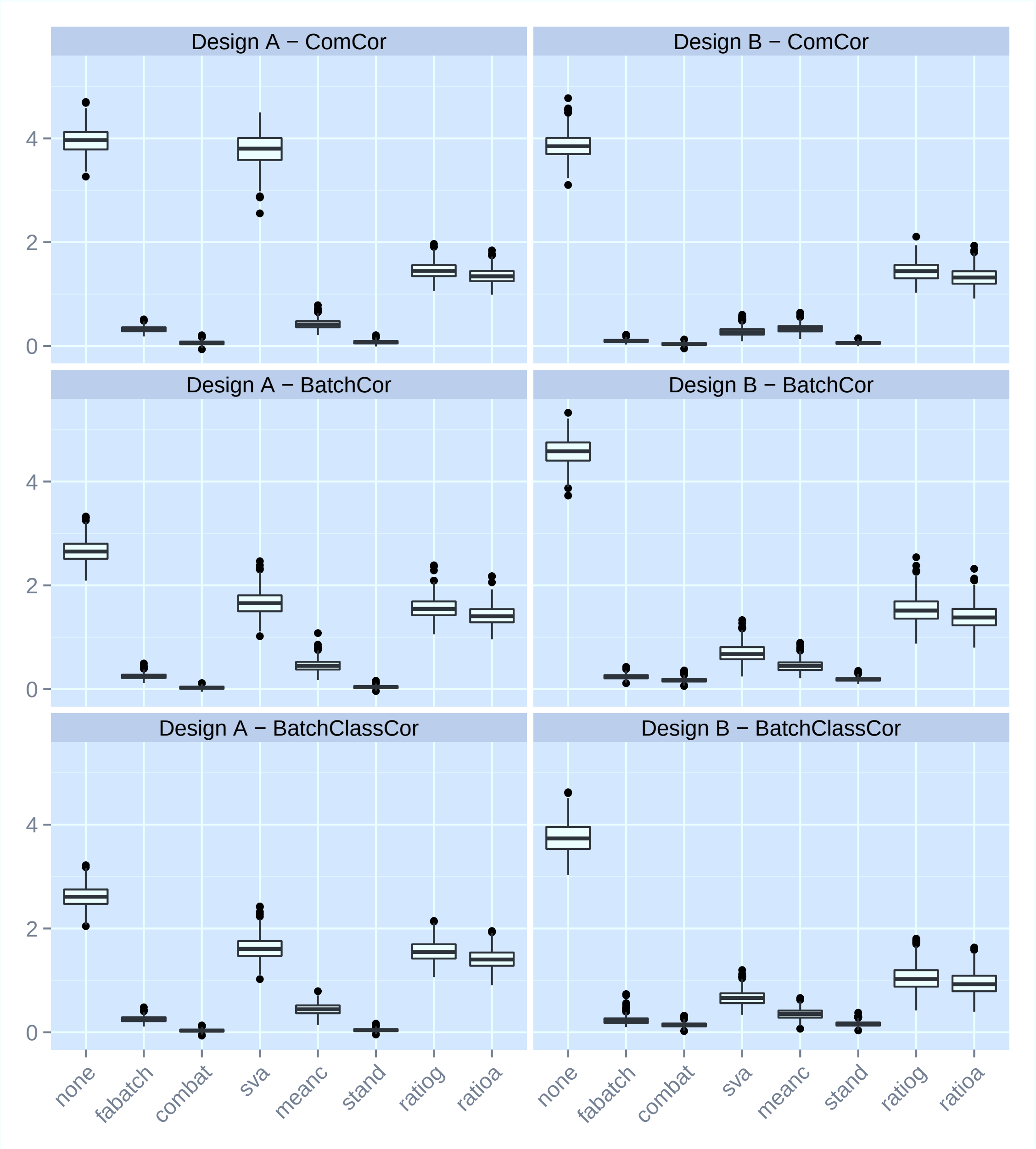

Fig. S10: Values of metric `klmetr` for all simulated datasets separated into simulation scenario and method.

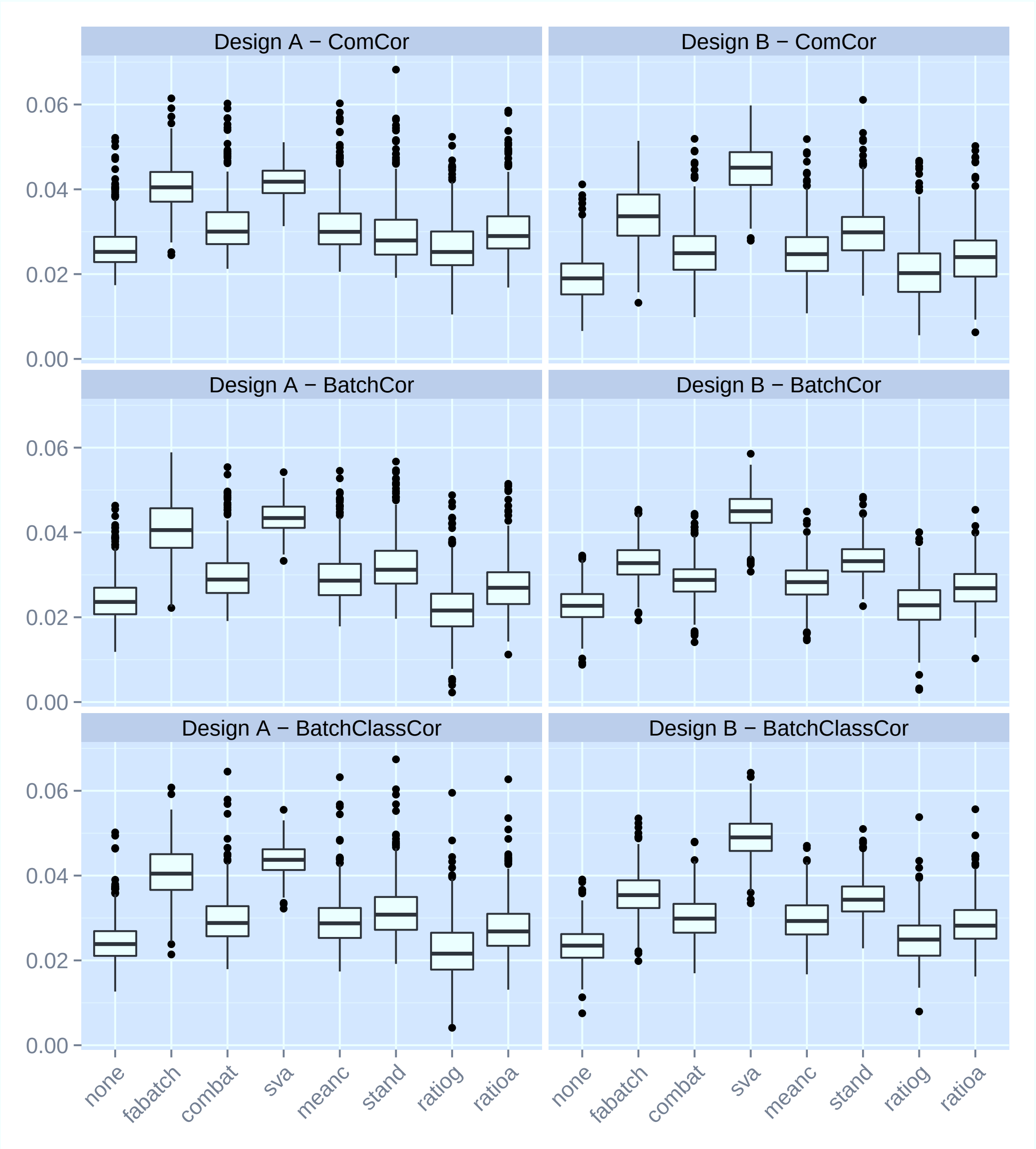

Fig. S11: Values of metric `pvca` for all simulated datasets separated into simulation scenario and method.

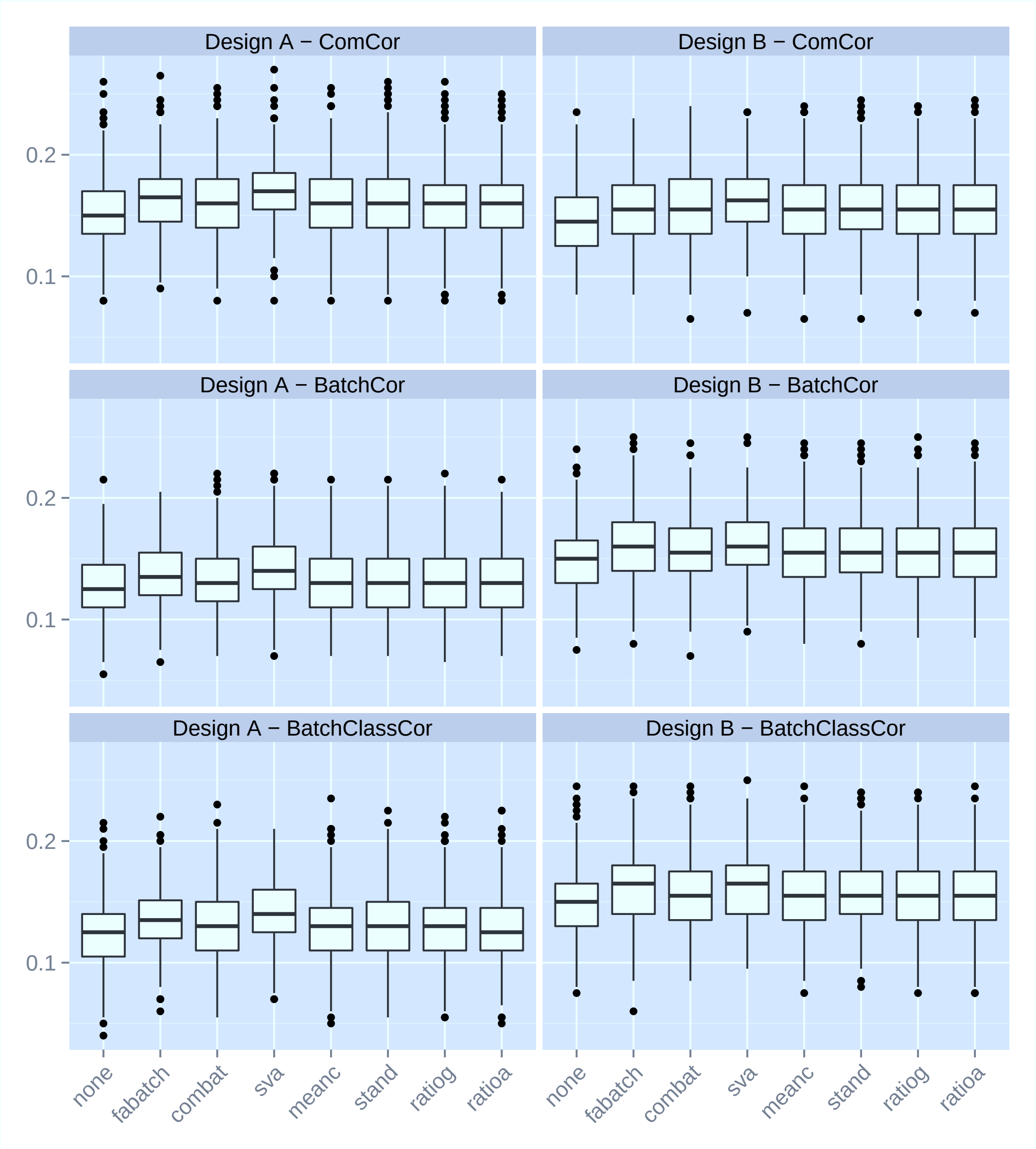

Fig. S12: Values of metric `diffexpr` for all simulated datasets separated into simulation scenario and method.

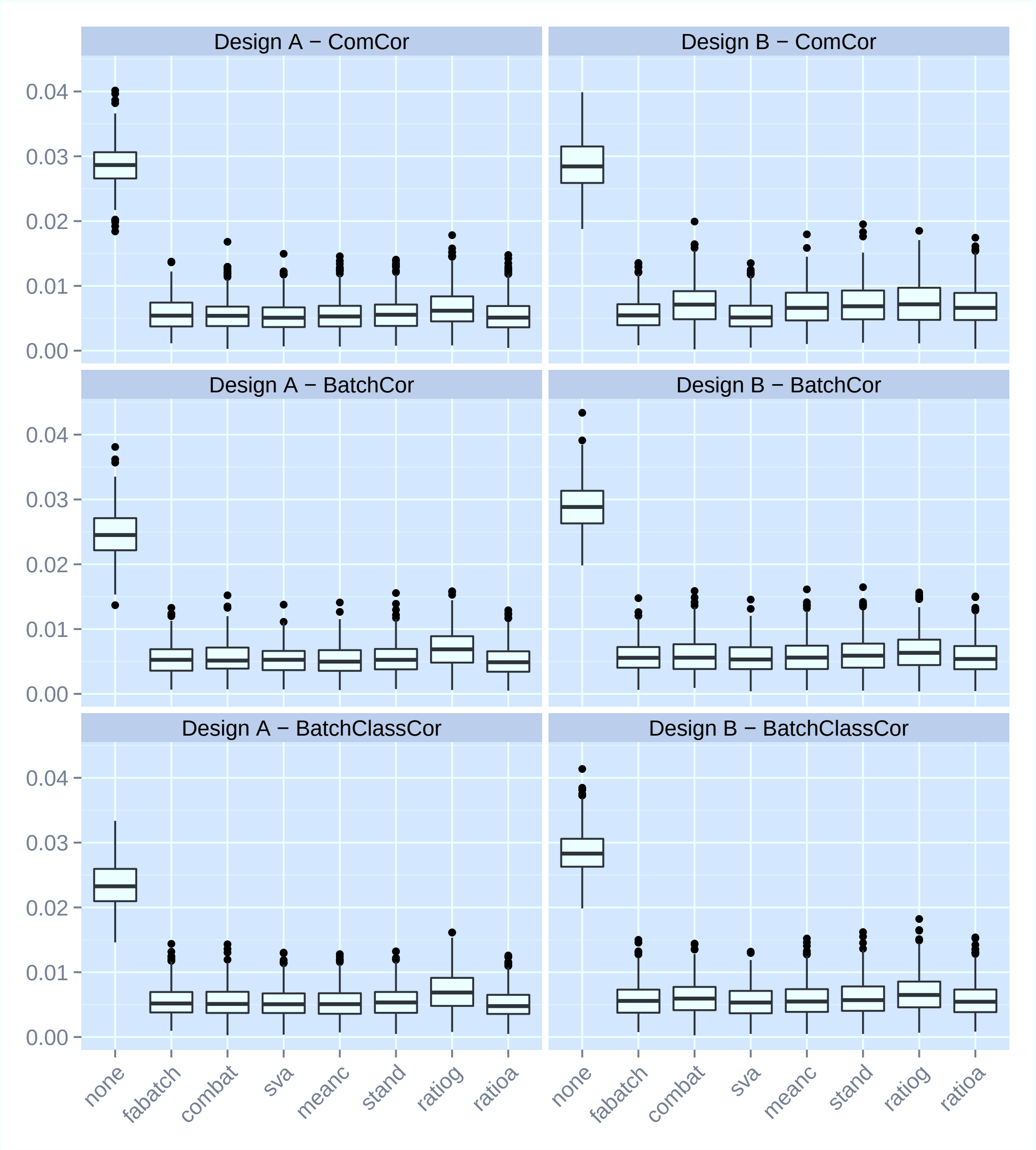

Fig. S13: Values of metric `skewdiv` for all simulated datasets separated into simulation scenario and method.

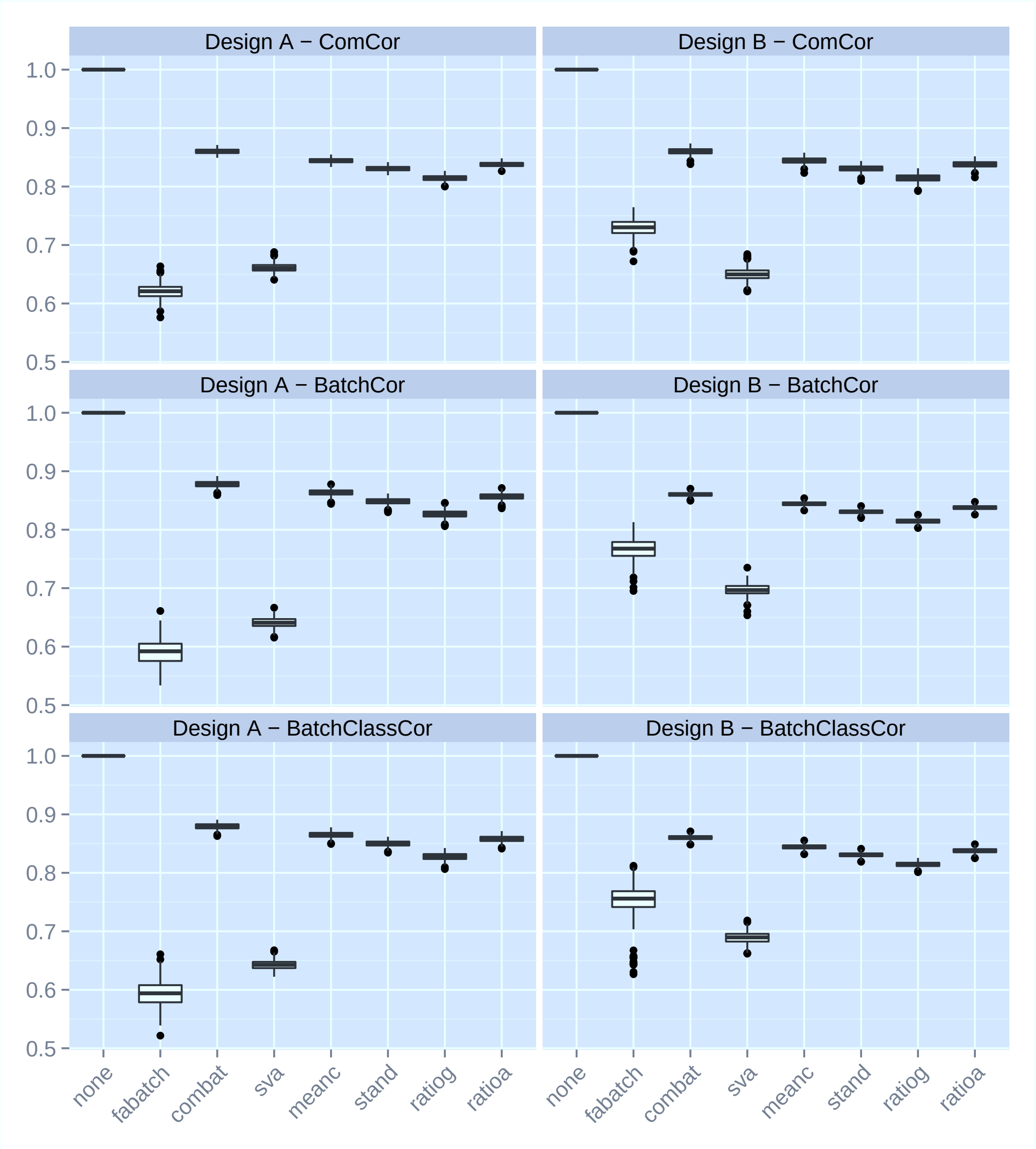

Fig. S14: Values of metric `corbeaf` for all simulated datasets separated into simulation scenario and method.

G Tables of the means of the metric values and their ranks for simulated datasets per method and scenario

Table S1: Means of the values of metric `sepscore` and of their ranks among the different methods over all simulated datasets separated into simulation scenario and method. In each row the results are listed in descending order according to mean performance in terms of the original values and their ranks, respectively.

Factor induced correlations - Common Correlations

mean values	**fabatch**<br>**0.04259**	combat<br>0.10329	stand<br>0.10983	meanc<br>0.26425	ratiog<br>0.34545	ratioa<br>0.36033	sva<br>0.40368	none<br>0.41272
mean ranks	**fabatch**<br>**1**	combat<br>2.272	stand<br>2.728	meanc<br>4.012	ratiog<br>5.159	ratioa<br>5.851	sva<br>7.236	none<br>7.742

Factor induced correlations - Batch-specific Correlations

mean values	**fabatch**<br>**0.03983**	combat<br>0.06467	stand<br>0.07055	meanc<br>0.2652	ratiog<br>0.33873	sva<br>0.34239	ratioa<br>0.3529	none<br>0.3767
mean ranks	**fabatch**<br>**1.184**	combat<br>2.168	stand<br>2.648	meanc<br>4.022	ratiog<br>5.807	sva<br>5.818	ratioa<br>6.485	none<br>7.868

Factor induced correlations - Batch-class-specific Correlations

mean values	**fabatch**<br>**0.04157**	combat<br>0.06515	stand<br>0.07127	meanc<br>0.263	ratiog<br>0.33364	sva<br>0.34064	ratioa<br>0.34989	none<br>0.37793
mean ranks	**fabatch**<br>**1.21**	combat<br>2.138	stand<br>2.652	meanc<br>4.024	ratiog<br>5.698	sva<br>5.841	ratioa<br>6.515	none<br>7.922

Correlations estimated on real data - Common Correlations

mean values	combat<br>0.09024	**fabatch**<br>**0.09931**	stand<br>0.11938	sva<br>0.17365	meanc<br>0.21915	ratiog<br>0.32436	ratioa<br>0.33918	none<br>0.41279
mean ranks	combat<br>1.252	**fabatch**<br>**1.825**	stand<br>2.939	sva<br>4.056	meanc<br>4.928	ratiog<br>6.215	ratioa<br>6.785	none<br>8

Correlations estimated on real data - Batch-specific Correlations

mean values	**fabatch**<br>**0.08246**	combat<br>0.09017	stand<br>0.10397	meanc<br>0.16851	sva<br>0.26416	ratiog<br>0.32547	ratioa<br>0.34139	none<br>0.46142
mean ranks	**fabatch**<br>**1.522**	combat<br>1.742	stand<br>2.806	meanc<br>3.932	sva<br>5.068	ratiog<br>6.098	ratioa<br>6.832	none<br>8

Correlations estimated on real data - Batch-class-specific Correlation

mean values	combat<br>0.10634	stand<br>0.11988	**fabatch**<br>**0.13248**	meanc<br>0.146	ratioa<br>0.28421	ratiog<br>0.29216	sva<br>0.30619	none<br>0.44789
mean ranks	combat<br>1.444	stand<br>2.616	**fabatch**<br>**2.809**	meanc<br>3.139	ratioa<br>5.574	ratiog<br>6.072	sva<br>6.346	none<br>8

Table S2: Means of the values of metric `avedist` and of their ranks among the different methods over all simulated datasets separated into simulation scenario and method. In each row the results are listed in descending order according to mean performance in terms of the original values and their ranks, respectively.

Factor induced correlations - Common Correlations

mean values	stand<br>37.42382	combat<br>37.53441	meanc<br>37.58121	ratioa<br>37.59354	ratiog<br>37.69106	**fabatch**<br>**40.49195**	none<br>40.95461	sva<br>42.10377
mean ranks	stand<br>1.016	combat<br>2.212	meanc<br>3.354	ratioa<br>3.446	ratiog<br>4.972	**fabatch**<br>**6.198**	none<br>6.802	sva<br>8

Factor induced correlations - Batch-specific Correlations

mean values	ratioa<br>37.40834	meanc<br>37.50749	stand<br>37.50828	ratiog<br>37.52306	combat<br>37.57991	none<br>39.7664	**fabatch**<br>**39.90564**	sva<br>41.54491
mean ranks	ratioa<br>1.228	stand<br>2.768	meanc<br>2.934	ratiog<br>3.448	combat<br>4.622	none<br>6.354	**fabatch**<br>**6.646**	sva<br>8

Factor induced correlations - Batch-class-specific Correlations

mean values	ratioa<br>37.39577	meanc<br>37.49533	stand<br>37.49946	ratiog<br>37.512	combat<br>37.57149	none<br>39.78901	**fabatch**<br>**39.93852**	sva<br>41.58123
mean ranks	ratioa<br>1.228	stand<br>2.752	meanc<br>2.878	ratiog<br>3.498	combat<br>4.644	none<br>6.358	**fabatch**<br>**6.642**	sva<br>8

Correlations estimated on real data - Common Correlations

mean values	stand<br>37.98839	ratioa<br>37.98975	meanc<br>38.01252	combat<br>38.05134	ratiog<br>38.09271	**fabatch**<br>**39.49123**	sva<br>39.82755	none<br>40.81266
mean ranks	stand<br>1.764	ratioa<br>2.064	meanc<br>2.598	combat<br>3.982	ratiog<br>4.592	**fabatch**<br>**6.044**	sva<br>6.956	none<br>8

Correlations estimated on real data - Batch-specific Correlations

mean values	ratioa<br>39.74457	ratiog<br>39.82068	meanc<br>39.95839	stand<br>40.1671	combat<br>40.1761	**fabatch**<br>**40.38741**	sva<br>40.44444	none<br>42.62075
mean ranks	ratioa<br>1.13	ratiog<br>2.152	meanc<br>3.112	stand<br>4.82	combat<br>5.024	**fabatch**<br>**5.736**	sva<br>6.026	none<br>8

Correlations estimated on real data - Batch-class-specific Correlation

mean values	ratioa<br>38.26292	ratiog<br>38.3701	meanc<br>38.66762	combat<br>38.93779	**fabatch**<br>**38.9477**	stand<br>38.97117	sva<br>39.28459	none<br>41.79299
mean ranks	ratioa<br>1.174	ratiog<br>2.218	meanc<br>3.354	**fabatch**<br>**4.69**	combat<br>4.946	stand<br>5.468	sva<br>6.15	none<br>8

Table S3: Means of the values of metric `klmetr` and of their ranks among the different methods over all simulated datasets separated into simulation scenario and method. In each row the results are listed in descending order according to mean performance in terms of the original values and their ranks, respectively.

Factor induced correlations - Common Correlations

mean values	combat	stand	**fabatch**	meanc	ratioa	ratiog	sva	none
	0.06141	0.07561	**0.32144**	0.4242	1.34917	1.44874	3.78636	3.96088
mean ranks	combat	stand	**fabatch**	meanc	ratioa	ratiog	sva	none
	1.344	1.656	**3.164**	3.836	5.002	5.998	7.342	7.658

Factor induced correlations - Batch-specific Correlations

mean values	combat	stand	**fabatch**	meanc	ratioa	ratiog	sva	none
	0.02796	0.03953	**0.25008**	0.45892	1.41785	1.56052	1.66582	2.65573
mean ranks	combat	stand	**fabatch**	meanc	ratioa	ratiog	sva	none
	1.344	1.656	**3.036**	3.964	5.198	6.376	6.426	8

Factor induced correlations - Batch-class-specific Correlations

mean values	combat	stand	**fabatch**	meanc	ratioa	ratiog	sva	none
	0.03325	0.04196	**0.25189**	0.44475	1.41077	1.55757	1.61859	2.61917
mean ranks	combat	stand	**fabatch**	meanc	ratioa	sva	ratiog	none
	1.394	1.606	**3.05**	3.95	5.2	6.382	6.418	8

Correlations estimated on real data - Common Correlations

mean values	combat	stand	**fabatch**	sva	meanc	ratioa	ratiog	none
	0.03725	0.06081	**0.09927**	0.27584	0.33733	1.32624	1.44356	3.86244
mean ranks	combat	stand	**fabatch**	sva	meanc	ratioa	ratiog	none
	1.278	1.938	**2.794**	4.286	4.704	6	7	8

Correlations estimated on real data - Batch-specific Correlations

mean values	combat	stand	**fabatch**	meanc	sva	ratioa	ratiog	none
	0.17189	0.19011	**0.23987**	0.44967	0.69586	1.40077	1.53715	4.57746
mean ranks	combat	stand	**fabatch**	meanc	sva	ratioa	ratiog	none
	1.382	1.888	**2.748**	4.066	4.922	5.994	7	8

Correlations estimated on real data - Batch-class-specific Correlation

mean values	combat	stand	**fabatch**	meanc	sva	ratioa	ratiog	none
	0.14284	0.16164	**0.23314**	0.35449	0.66427	0.94171	1.0381	3.75182
mean ranks	combat	stand	**fabatch**	meanc	sva	ratioa	ratiog	none
	1.372	1.874	**2.894**	3.882	5.218	5.856	6.904	8

Table S4: Means of the values of metric `pvca` and of their ranks among the different methods over all simulated datasets separated into simulation scenario and method. In each row the results are listed in descending order according to mean performance in terms of the original values and their ranks, respectively.

Factor induced correlations - Common Correlations

mean values	sva<br>0.04174	**fabatch**<br>**0.04079**	combat<br>0.03155	meanc<br>0.03139	ratioa<br>0.03047	stand<br>0.02972	none<br>0.02643	ratiog<br>0.02634
mean ranks	sva<br>1.69	**fabatch**<br>**1.88**	combat<br>3.874	meanc<br>4.086	ratioa<br>4.752	stand<br>5.386	ratiog<br>7.044	none<br>7.288

Factor induced correlations - Batch-specific Correlations

mean values	sva<br>0.04362	**fabatch**<br>**0.04099**	stand<br>0.03228	combat<br>0.02981	meanc<br>0.02956	ratioa<br>0.02743	none<br>0.02432	ratiog<br>0.02209
mean ranks	sva<br>1.482	**fabatch**<br>**1.868**	stand<br>3.212	combat<br>4.372	meanc<br>4.696	ratioa<br>5.626	none<br>7.122	ratiog<br>7.622

Factor induced correlations - Batch-class-specific Correlations

mean values	sva<br>0.04376	**fabatch**<br>**0.04053**	stand<br>0.03172	combat<br>0.02988	meanc<br>0.02964	ratioa<br>0.02771	none<br>0.02459	ratiog<br>0.02254
mean ranks	sva<br>1.37	**fabatch**<br>**1.986**	stand<br>3.436	combat<br>4.362	meanc<br>4.604	ratioa<br>5.568	none<br>7.154	ratiog<br>7.52

Correlations estimated on real data - Common Correlations

mean values	sva<br>0.04484	**fabatch**<br>**0.03385**	stand<br>0.0301	combat<br>0.02528	meanc<br>0.02505	ratioa<br>0.0242	ratiog<br>0.02065	none<br>0.01921
mean ranks	sva<br>1.032	**fabatch**<br>**2.394**	stand<br>2.932	combat<br>4.804	meanc<br>5.032	ratioa<br>5.276	ratiog<br>6.96	none<br>7.57

Correlations estimated on real data - Batch-specific Correlations

mean values	sva<br>0.04494	stand<br>0.03349	**fabatch**<br>**0.03294**	combat<br>0.02878	meanc<br>0.02822	ratioa<br>0.02702	ratiog<br>0.0229	none<br>0.02276
mean ranks	sva<br>1.008	stand<br>2.54	**fabatch**<br>**2.888**	combat<br>4.474	meanc<br>4.96	ratioa<br>5.41	ratiog<br>7.248	none<br>7.472

Correlations estimated on real data - Batch-class-specific Correlation

mean values	sva<br>0.04893	**fabatch**<br>**0.03542**	stand<br>0.03463	combat<br>0.0301	meanc<br>0.02965	ratioa<br>0.02862	ratiog<br>0.02497	none<br>0.02351
mean ranks	sva<br>1.008	stand<br>2.662	**fabatch**<br>**2.76**	combat<br>4.602	meanc<br>5.002	ratioa<br>5.33	ratiog<br>7.042	none<br>7.594

Table S5: Means of the values of metric `diffexpr` and of their ranks among the different methods over all simulated datasets separated into simulation scenario and method. In each row the results are listed in descending order according to mean performance in terms of the original values and their ranks, respectively.

Factor induced correlations - Common Correlations

mean values	sva 0.16971	**fabatch 0.16407**	combat 0.16098	stand 0.16097	meanc 0.1603	ratioa 0.15957	ratiog 0.15924	none 0.15215
mean ranks	sva 3.192	**fabatch 3.926**	combat 4.273	stand 4.361	meanc 4.558	ratioa 4.753	ratiog 4.844	none 6.093

Factor induced correlations - Batch-specific Correlations

mean values	sva 0.14241	**fabatch 0.13769**	combat 0.13267	stand 0.13246	meanc 0.13195	ratioa 0.13094	ratiog 0.13074	none 0.12615
mean ranks	sva 3.202	**fabatch 3.821**	combat 4.442	stand 4.448	meanc 4.548	ratioa 4.849	ratiog 4.898	none 5.792

Factor induced correlations - Batch-class-specific Correlations

mean values	sva 0.1414	**fabatch 0.13553**	stand 0.12975	combat 0.12949	meanc 0.12902	ratiog 0.12798	ratioa 0.12777	none 0.12367
mean ranks	sva 2.936	**fabatch 3.801**	stand 4.477	combat 4.508	meanc 4.618	ratiog 4.909	ratioa 5.015	none 5.736

Correlations estimated on real data - Common Correlations

mean values	sva 0.16337	stand 0.15697	combat 0.1568	meanc 0.15587	**fabatch 0.15579**	ratioa 0.15554	ratiog 0.1552	none 0.14817
mean ranks	sva 3.62	stand 4.134	combat 4.163	meanc 4.433	**fabatch 4.56**	ratioa 4.572	ratiog 4.64	none 5.878

Correlations estimated on real data - Batch-specific Correlations

mean values	sva 0.16086	**fabatch 0.16064**	combat 0.15724	stand 0.15721	meanc 0.15659	ratiog 0.15598	ratioa 0.15595	none 0.14802
mean ranks	**fabatch 3.772**	sva 3.791	combat 4.236	stand 4.299	meanc 4.448	ratiog 4.628	ratioa 4.635	none 6.191

Correlations estimated on real data - Batch-class-specific Correlation

mean values	sva 0.16286	**fabatch 0.16221**	stand 0.158	combat 0.15792	meanc 0.15711	ratioa 0.15637	ratiog 0.15621	none 0.14918
mean ranks	sva 3.58	**fabatch 3.689**	stand 4.235	combat 4.314	meanc 4.528	ratioa 4.755	ratiog 4.803	none 6.096

Table S6: Means of the values of metric `skewdiv` and of their ranks among the different methods over all simulated datasets separated into simulation scenario and method. In each row the results are listed in descending order according to mean performance in terms of the original values and their ranks, respectively.

Factor induced correlations - Common Correlations

mean values	sva	ratioa	meanc	combat	**fabatch**	stand	ratiog	none
	0.00535	0.0054	0.00547	0.0055	**0.00571**	0.00573	0.00651	0.02865
mean ranks	ratioa	meanc	sva	combat	**fabatch**	stand	ratiog	none
	3.514	3.696	3.832	3.852	**4.124**	4.252	4.73	8

Factor induced correlations - Batch-specific Correlations

mean values	ratioa	meanc	sva	stand	**fabatch**	combat	ratiog	none
	0.00509	0.00519	0.00524	0.00543	**0.00544**	0.00556	0.00701	0.0246
mean ranks	ratioa	meanc	sva	**fabatch**	stand	combat	ratiog	none
	3.402	3.612	3.722	**3.97**	4	4.08	5.214	8

Factor induced correlations - Batch-class-specific Correlations

mean values	ratioa	meanc	sva	combat	**fabatch**	stand	ratiog	none
	0.00515	0.00527	0.00528	0.00544	**0.00548**	0.0055	0.00712	0.02349
mean ranks	ratioa	meanc	sva	**fabatch**	combat	stand	ratiog	none
	3.486	3.724	3.796	**3.872**	3.89	4.022	5.21	8

Correlations estimated on real data - Common Correlations

mean values	sva	**fabatch**	meanc	ratioa	stand	combat	ratiog	none
	0.00545	**0.00571**	0.00692	0.00695	0.00716	0.00722	0.00751	0.02868
mean ranks	sva	**fabatch**	meanc	ratioa	combat	stand	ratiog	none
	3.072	**3.266**	4.062	4.094	4.426	4.432	4.648	8

Correlations estimated on real data - Batch-specific Correlations

mean values	sva	ratioa	**fabatch**	meanc	combat	stand	ratiog	none
	0.00552	0.00572	**0.00574**	0.00584	0.00591	0.00608	0.00659	0.02892
mean ranks	ratioa	sva	meanc	**fabatch**	combat	stand	ratiog	none
	3.592	3.762	3.832	**3.848**	3.956	4.47	4.54	8

Correlations estimated on real data - Batch-class-specific Correlation

mean values	sva	**fabatch**	ratioa	meanc	stand	combat	ratiog	none
	0.00547	**0.00573**	0.00573	0.00581	0.00601	0.00602	0.00674	0.0285
mean ranks	ratioa	sva	meanc	**fabatch**	combat	stand	ratiog	none
	3.566	3.626	3.876	**3.95**	4.074	4.222	4.686	8

Table S7: Means of the values of metric `corbeaf` and of their ranks among the different methods over all simulated datasets separated into simulation scenario and method. In each row the results are listed in descending order according to mean performance in terms of the original values and their ranks, respectively.

Factor induced correlations - Common Correlations

mean values	none<br>1	combat<br>0.86018	meanc<br>0.84427	ratioa<br>0.83774	stand<br>0.83067	ratiog<br>0.81446	sva<br>0.66124	**fabatch**<br>**0.62064**
mean ranks	none<br>1	combat<br>2	meanc<br>3	ratioa<br>4	stand<br>5	ratiog<br>6	sva<br>7	**fabatch**<br>**8**

Factor induced correlations - Batch-specific Correlations

mean values	none<br>1	combat<br>0.87781	meanc<br>0.86351	ratioa<br>0.85656	stand<br>0.84845	ratiog<br>0.82682	sva<br>0.64111	**fabatch**<br>**0.59071**
mean ranks	none<br>1	combat<br>2	meanc<br>3	ratioa<br>4	stand<br>5	ratiog<br>6	sva<br>7.006	**fabatch**<br>**7.994**

Factor induced correlations - Batch-class-specific Correlations

mean values	none<br>1	combat<br>0.87943	meanc<br>0.8651	ratioa<br>0.85816	stand<br>0.85006	ratiog<br>0.828	sva<br>0.64263	**fabatch**<br>**0.59331**
mean ranks	none<br>1	combat<br>2	meanc<br>3	ratioa<br>4	stand<br>5	ratiog<br>6	sva<br>7.006	**fabatch**<br>**7.994**

Correlations estimated on real data - Common Correlations

mean values	none<br>1	combat<br>0.86037	meanc<br>0.84442	ratioa<br>0.8379	stand<br>0.83086	ratiog<br>0.81468	**fabatch**<br>**0.72987**	sva<br>0.64994
mean ranks	none<br>1	combat<br>2	meanc<br>3	ratioa<br>4	stand<br>5	ratiog<br>6	**fabatch**<br>**7**	sva<br>8

Correlations estimated on real data - Batch-specific Correlations

mean values	none<br>1	combat<br>0.86035	meanc<br>0.84433	ratioa<br>0.83781	stand<br>0.83078	ratiog<br>0.81458	**fabatch**<br>**0.76653**	sva<br>0.69691
mean ranks	none<br>1	combat<br>2	meanc<br>3	ratioa<br>4	stand<br>5	ratiog<br>6	**fabatch**<br>**7.002**	sva<br>7.998

Correlations estimated on real data - Batch-class-specific Correlation

mean values	none<br>1	combat<br>0.86023	meanc<br>0.84423	ratioa<br>0.83771	stand<br>0.83071	ratiog<br>0.81446	**fabatch**<br>**0.75314**	sva<br>0.68924
mean ranks	none<br>1	combat<br>2	meanc<br>3	ratioa<br>4	stand<br>5	ratiog<br>6.002	**fabatch**<br>**7.024**	sva<br>7.974

[1] Johnson WE, Rabinovic A, Li C. Adjusting batch effects in microarray expression data using Empirical Bayes methods. Biostatistics. 2007;8:118–127.

[2] Parker HS, Bravo HC, Leek JT. Removing batch effects for prediction problems with frozen surrogate variable analysis. PeerJ. 2014;2:e561.

[3] Leek JT, Storey JD. Capturing heterogeneity in gene expression studies by surrogate variable analysis. PLoS Genetics. 2007;3:1724–1735.

[4] Luo J, Schumacher M, Scherer A, Sanoudou D, Megherbi D, Davison T, et al. A comparison of batch effect removal methods for enhancement of prediction performance using MAQC-II microarray gene expression data. The Pharmacogenomics Journal. 2010;10:278–291.

[5] Moen EL, Zhang X, Mu W, Delaney SM, Wing C, McQuade J, et al. Genome-Wide Variation of Cytosine Modifications Between European and African Populations and the Implications for Complex Traits. Genetics. 2013;194:987–996.

[6] Godfrey AC, Xu Z, Weinberg CR, Getts RC, Wade PA, DeRoo LA, et al. Serum microRNA expression as an early marker for breast cancer risk in prospectively collected samples from the Sister Study cohort. Breast Cancer Research. 2013;15:R42.
