# Supplementary material for: Combining location-and-scale batch effect adjustment with data cleaning by latent factor adjustment
Source: BMC Bioinformatics. 2016 Jan 12;17:27. doi: 10.1186/s12859-015-0870-z (PMC4710051; doi:10.1186/s12859-015-0870-z)
Supplement: Additional file 2 — This folder contains all necessary R-Code to reproduce and evaluate the real-data analyses and simulations, as well as Rda-files enabling fast evaluation of the corresponding results. (ZIP 2406 kb) [file 12859_2015_870_MOESM2_ESM.zip › FAbatchPaper/Results/SupplementaryFigure10.pdf]

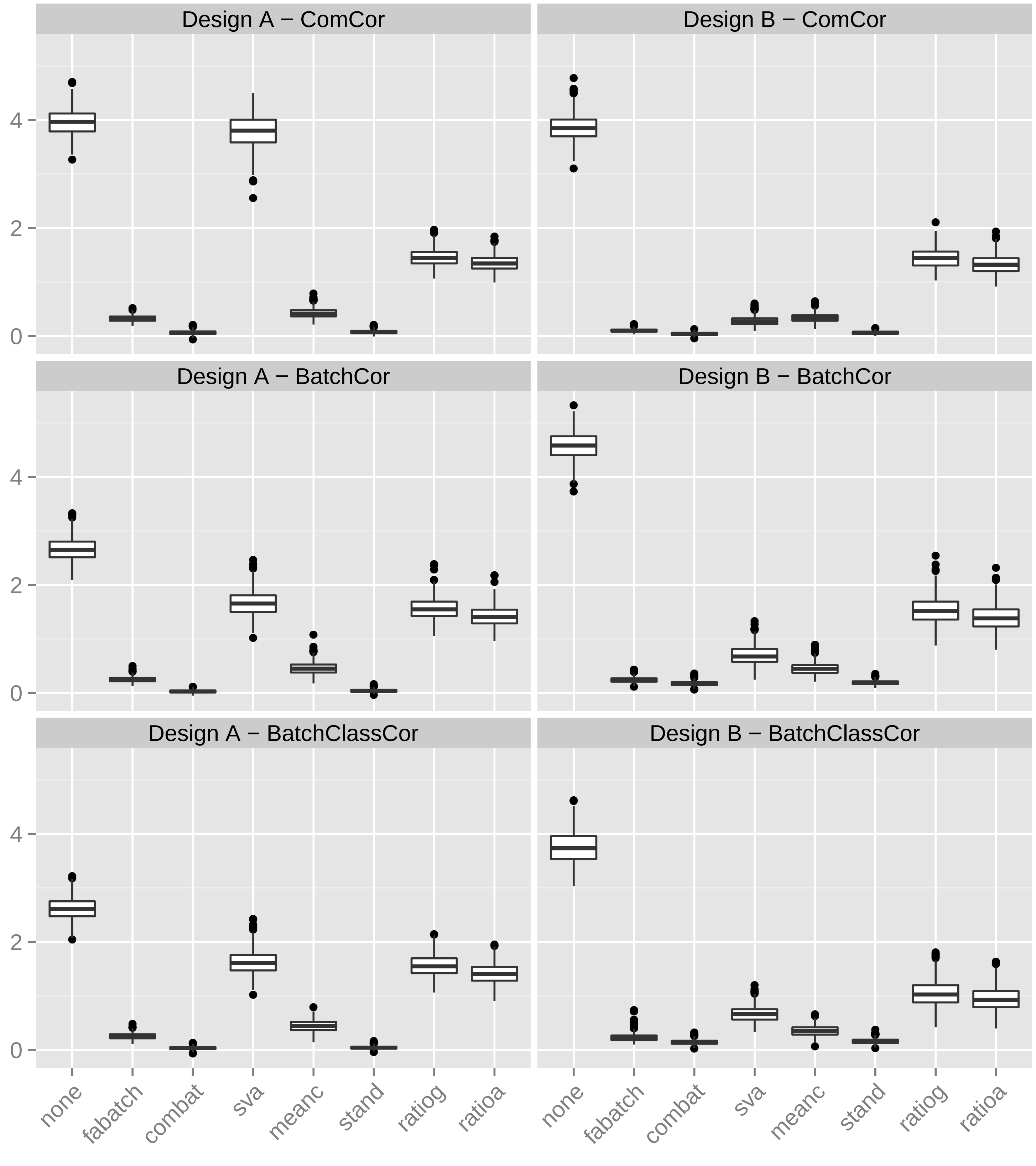
Design A − ComCor
Design B − ComCor
Design A − BatchCor
Design B − BatchCor
Design A − BatchClassCor
Design B − BatchClassCor
0
2
4
none
fabatch
combat
sva
meanc
stand
ratiog
ratioa
