# Supplementary figures and images for: Combining location-and-scale batch effect adjustment with data cleaning by latent factor adjustment
Source: BMC Bioinformatics. 2016 Jan 12;17:27. doi: 10.1186/s12859-015-0870-z (PMC4710051; doi:10.1186/s12859-015-0870-z)

Without batch effect adjustment

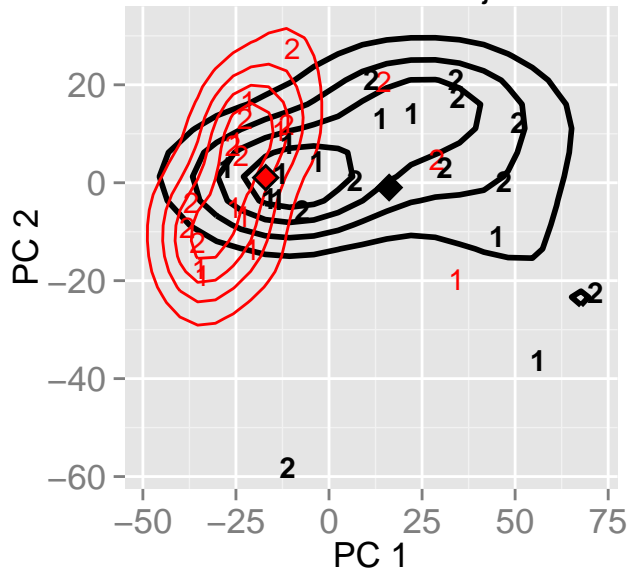

ComBat

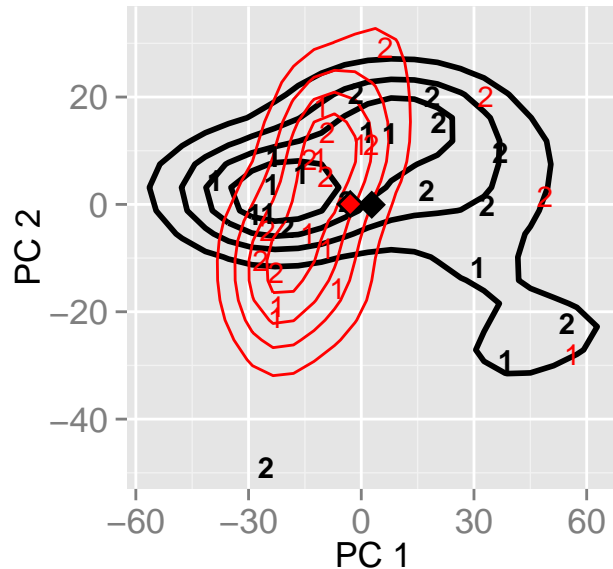

SVA

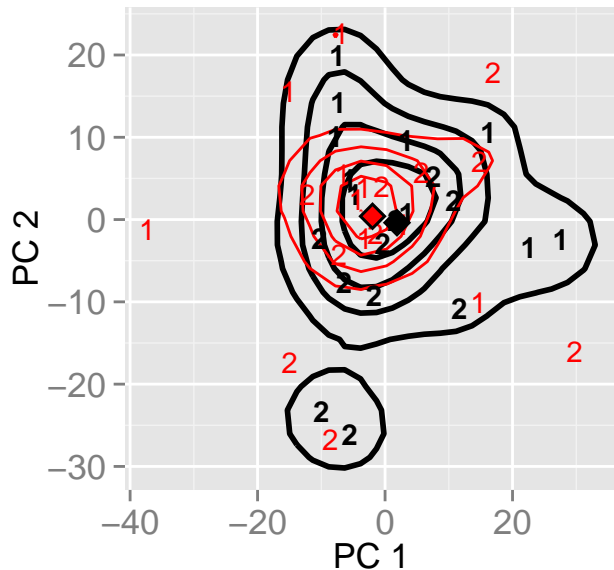

FABatch

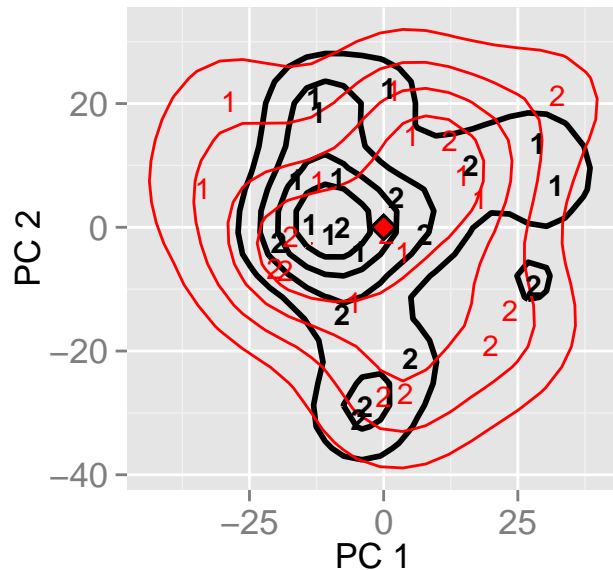

Supplement: Additional file 2 — This folder contains all necessary R-Code to reproduce and evaluate the real-data analyses and simulations, as well as Rda-files enabling fast evaluation of the corresponding results. (ZIP 2406 kb) [file 12859_2015_870_MOESM2_ESM.zip › FAbatchPaper/Results/Figure1.pdf]

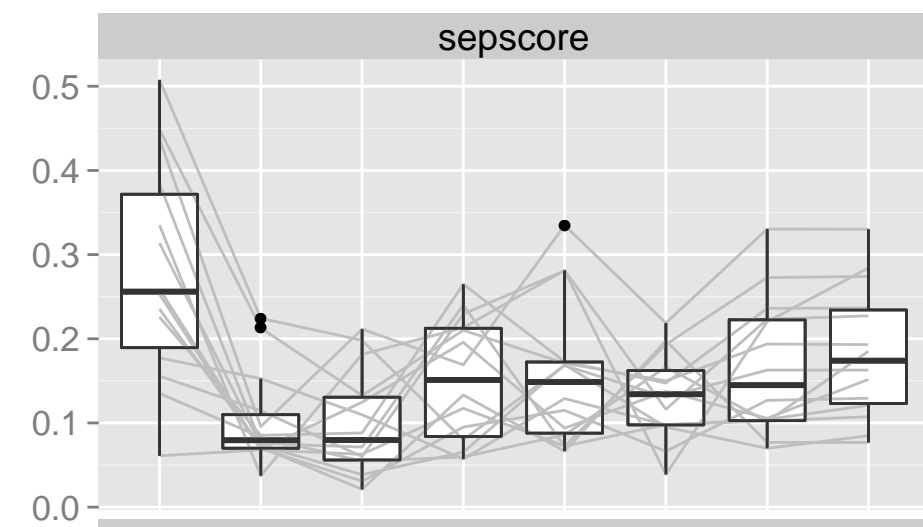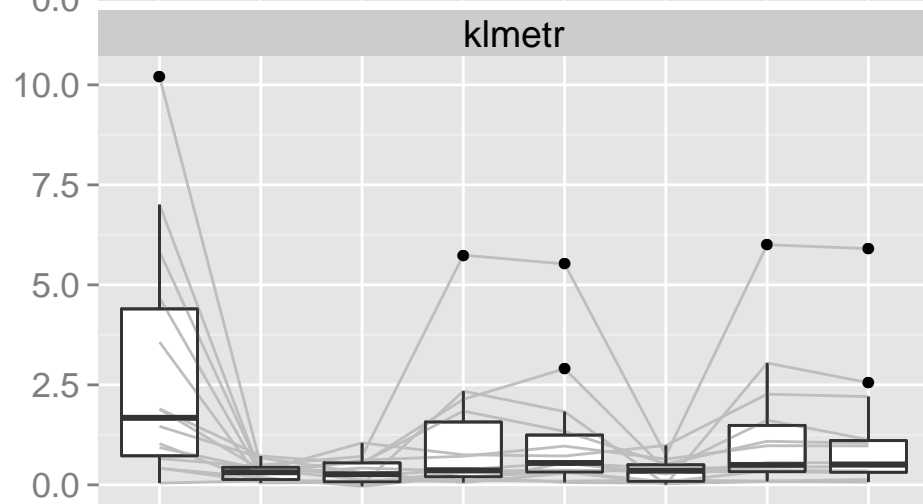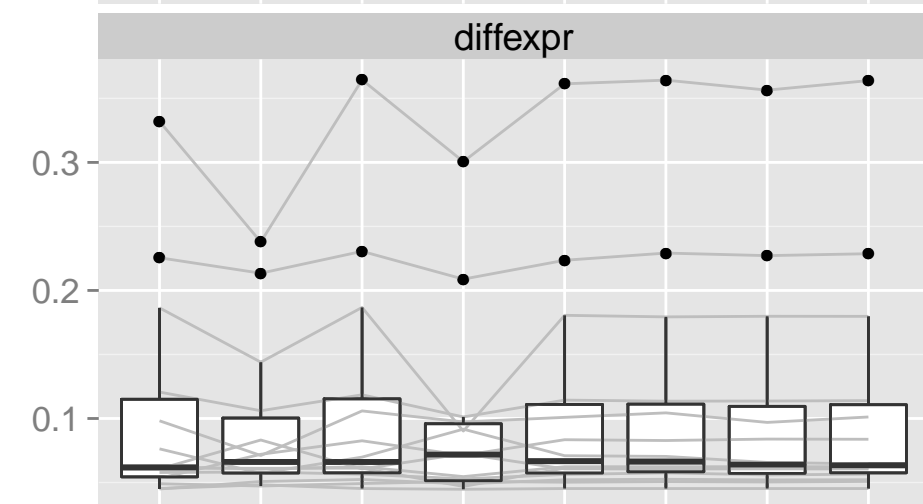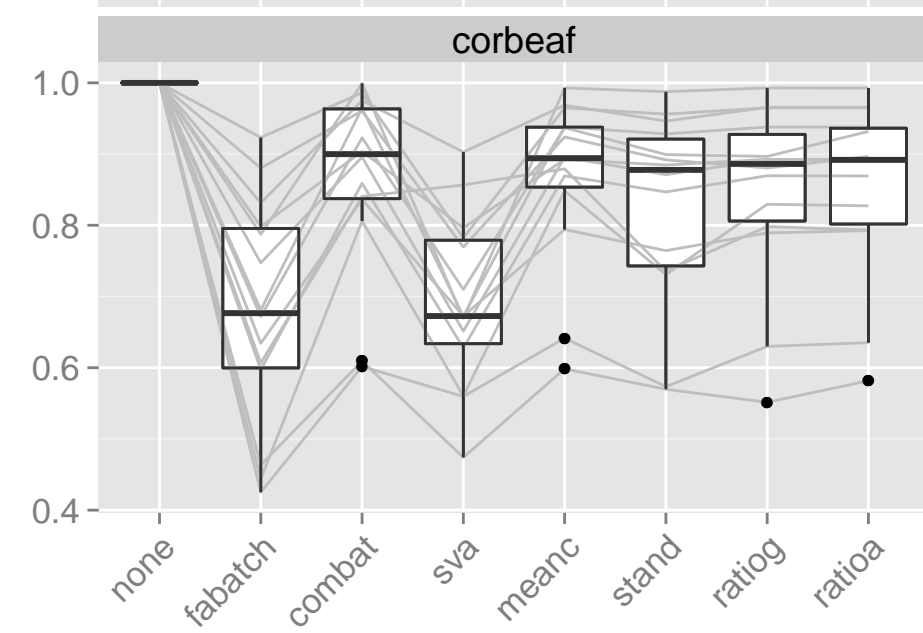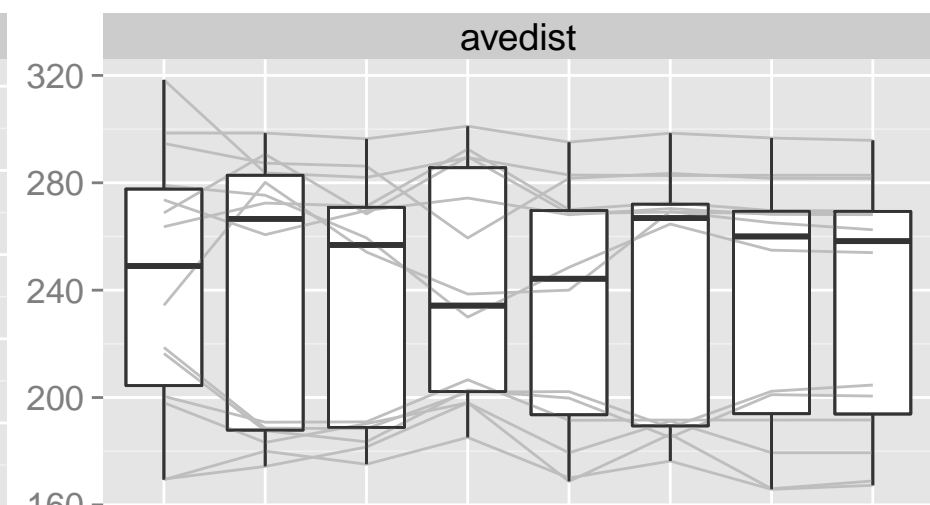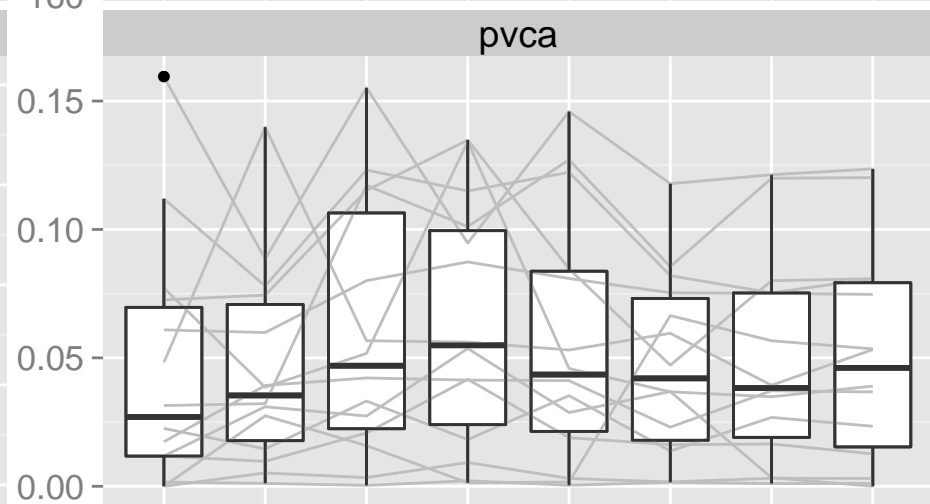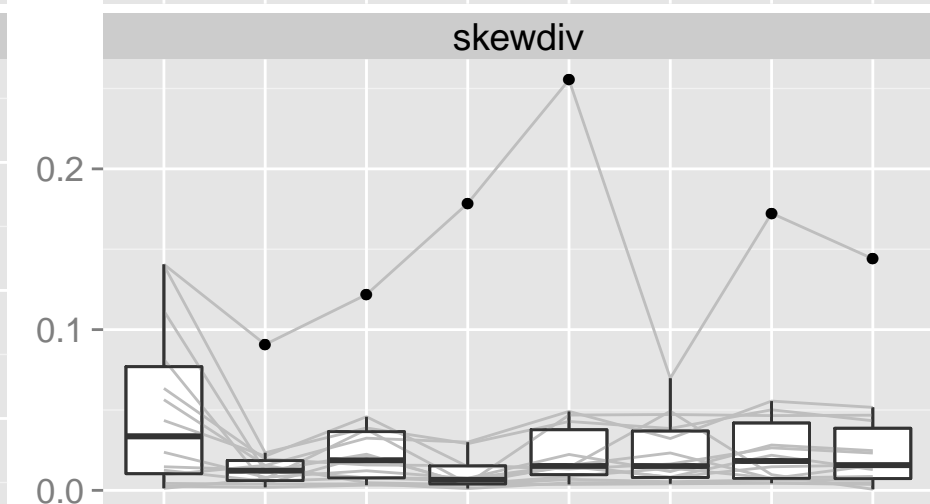

Supplement: Additional file 2 — This folder contains all necessary R-Code to reproduce and evaluate the real-data analyses and simulations, as well as Rda-files enabling fast evaluation of the corresponding results. (ZIP 2406 kb) [file 12859_2015_870_MOESM2_ESM.zip › FAbatchPaper/Results/Figure2.pdf]

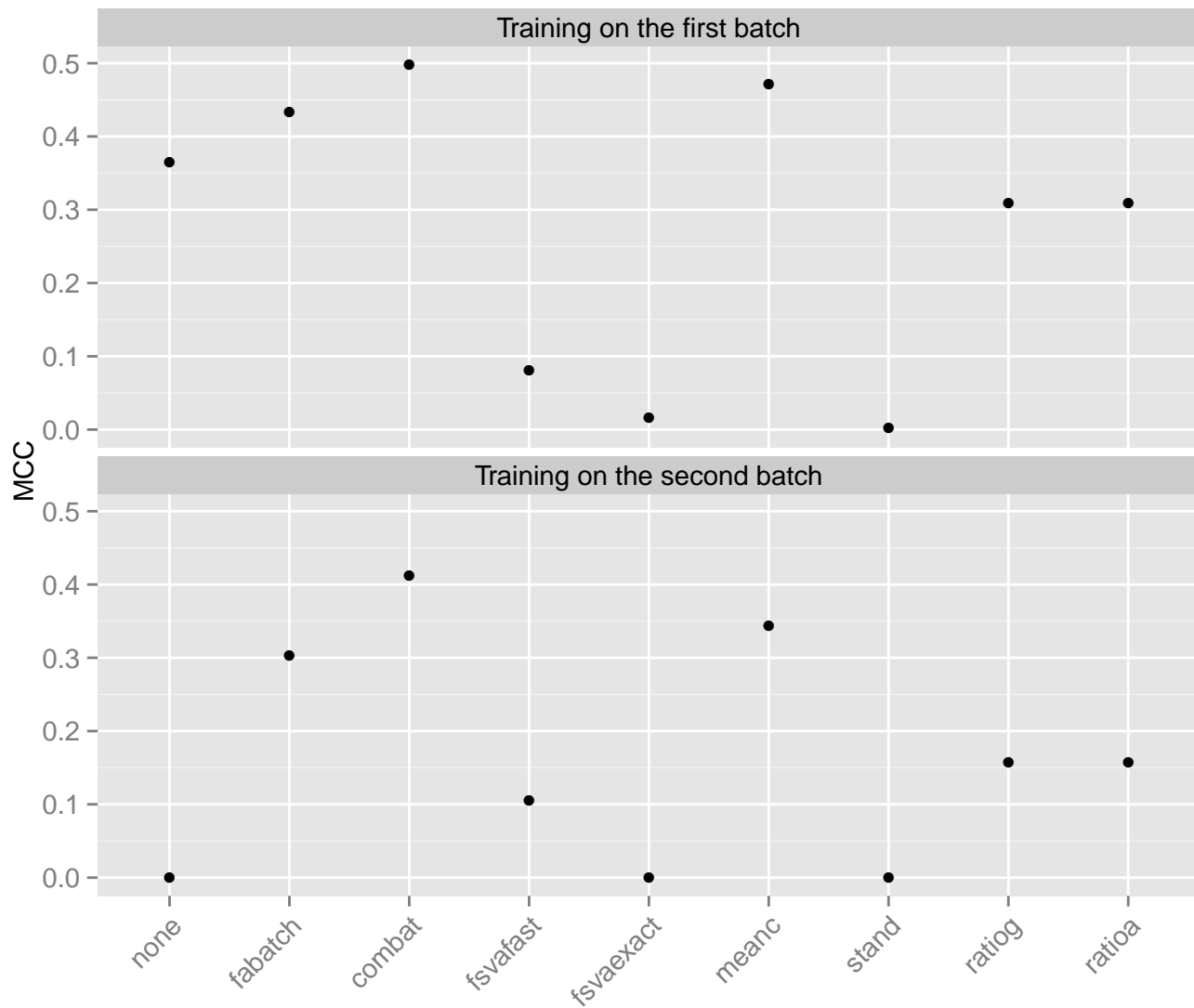

Supplement: Additional file 2 — This folder contains all necessary R-Code to reproduce and evaluate the real-data analyses and simulations, as well as Rda-files enabling fast evaluation of the corresponding results. (ZIP 2406 kb) [file 12859_2015_870_MOESM2_ESM.zip › FAbatchPaper/Results/Figure3.pdf]

none

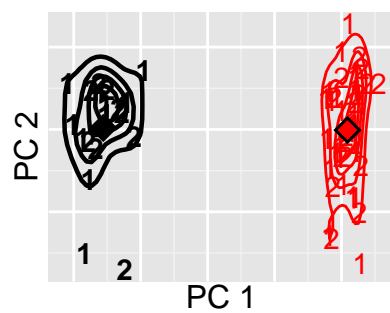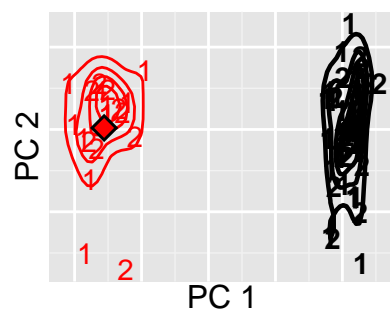

fabatch

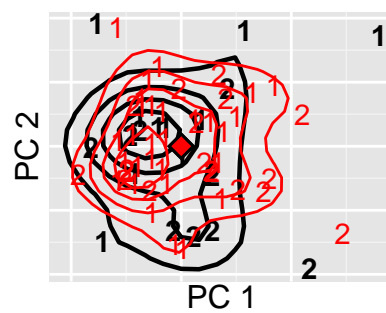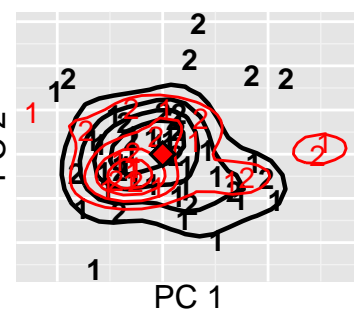

combat

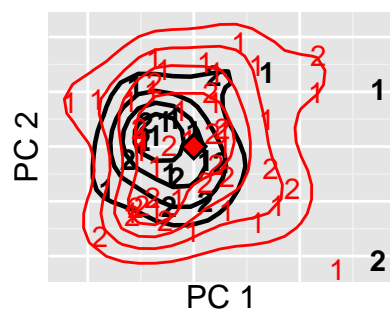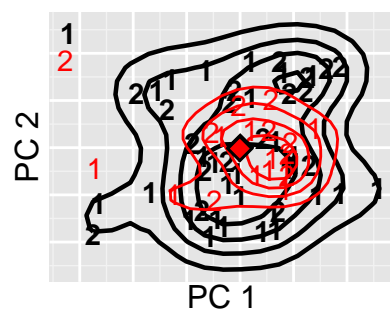

fsvafast

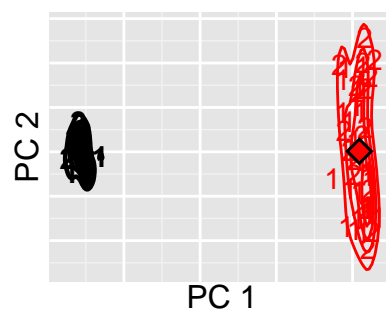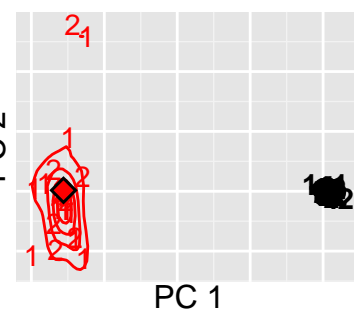

fsvaeexact

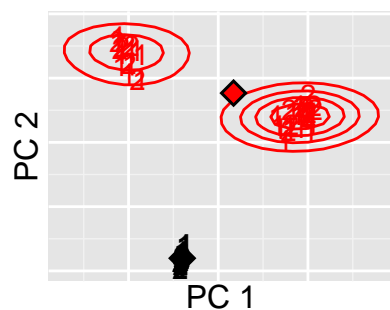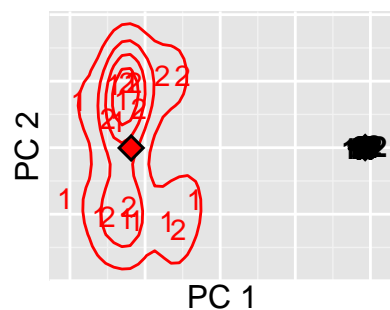

meanc

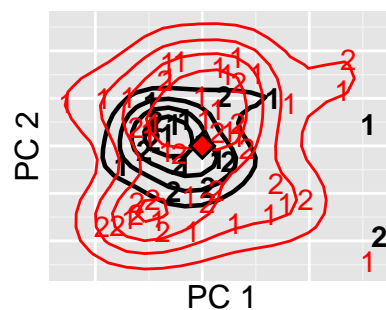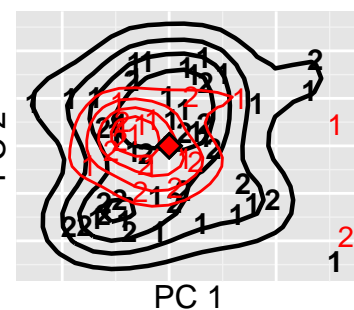

stand

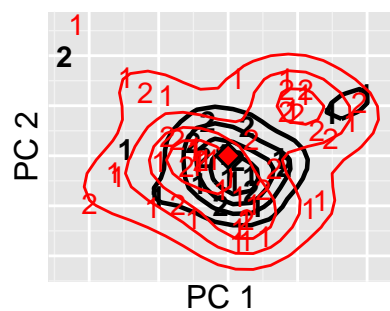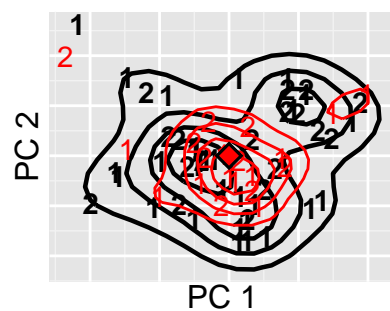

ratiog

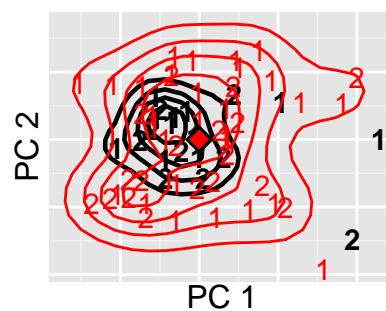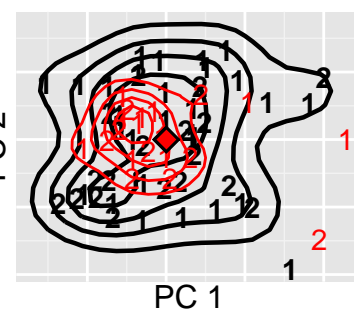

ratioa

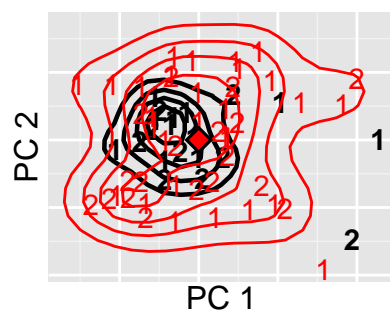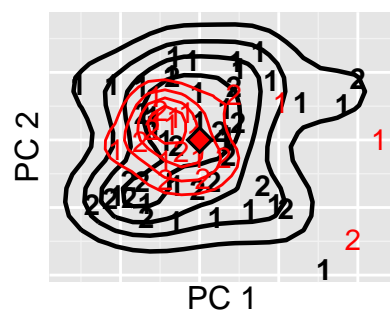

Supplement: Additional file 2 — This folder contains all necessary R-Code to reproduce and evaluate the real-data analyses and simulations, as well as Rda-files enabling fast evaluation of the corresponding results. (ZIP 2406 kb) [file 12859_2015_870_MOESM2_ESM.zip › FAbatchPaper/Results/Figure4.pdf]

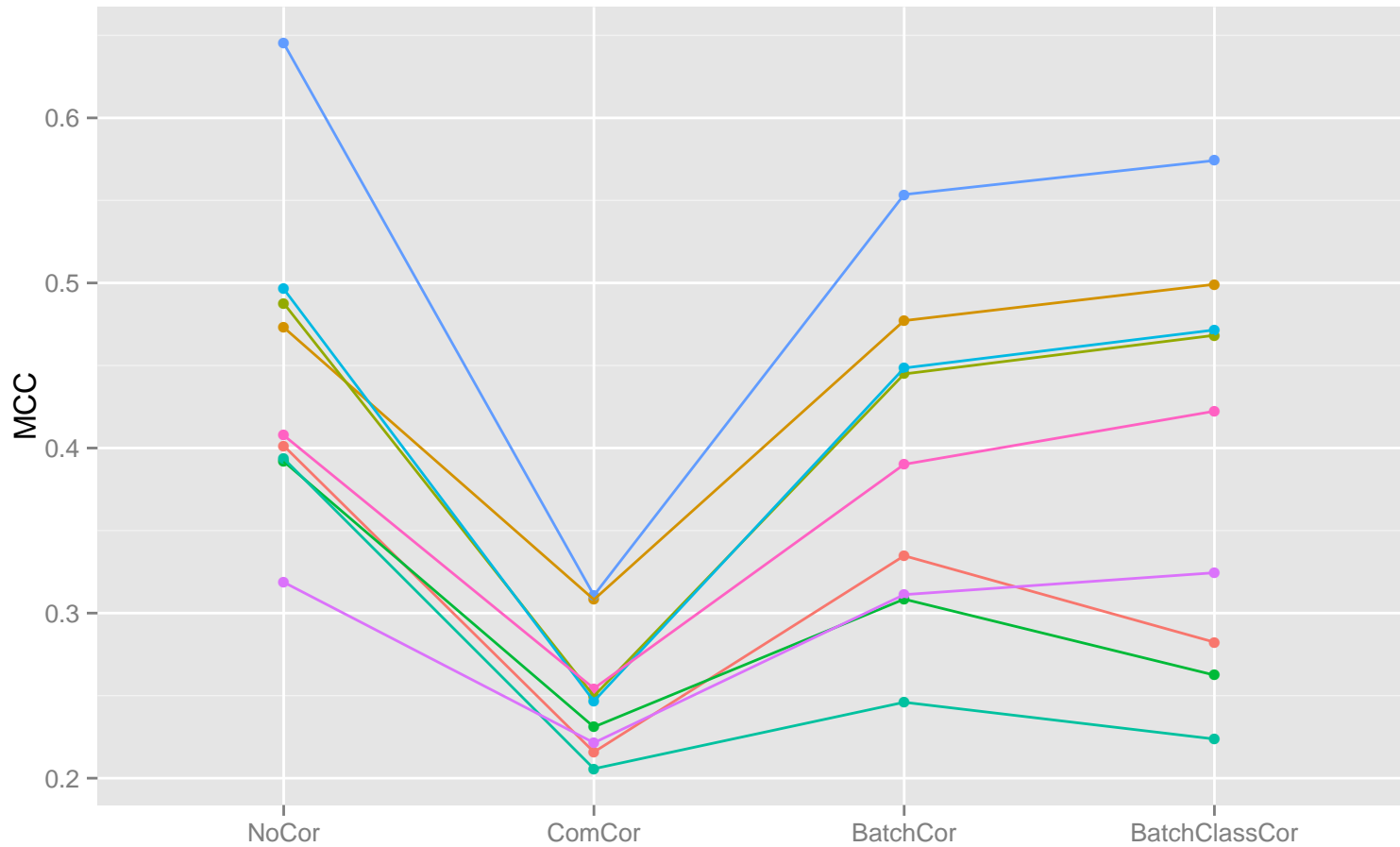

Supplement: Additional file 2 — This folder contains all necessary R-Code to reproduce and evaluate the real-data analyses and simulations, as well as Rda-files enabling fast evaluation of the corresponding results. (ZIP 2406 kb) [file 12859_2015_870_MOESM2_ESM.zip › FAbatchPaper/Results/Figure5.pdf]

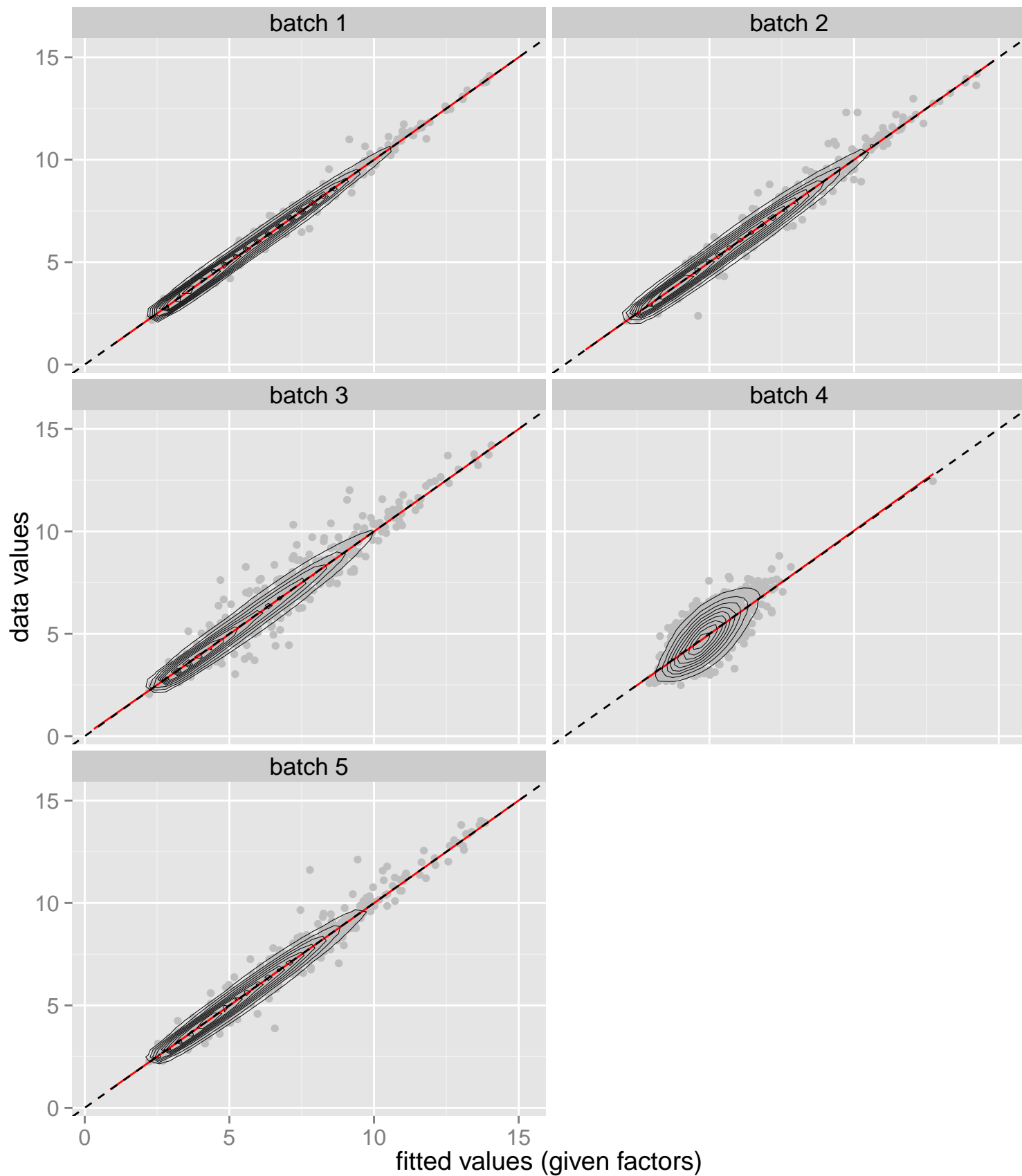

Supplement: Additional file 2 — This folder contains all necessary R-Code to reproduce and evaluate the real-data analyses and simulations, as well as Rda-files enabling fast evaluation of the corresponding results. (ZIP 2406 kb) [file 12859_2015_870_MOESM2_ESM.zip › FAbatchPaper/Results/SupplementaryFigure1.pdf]

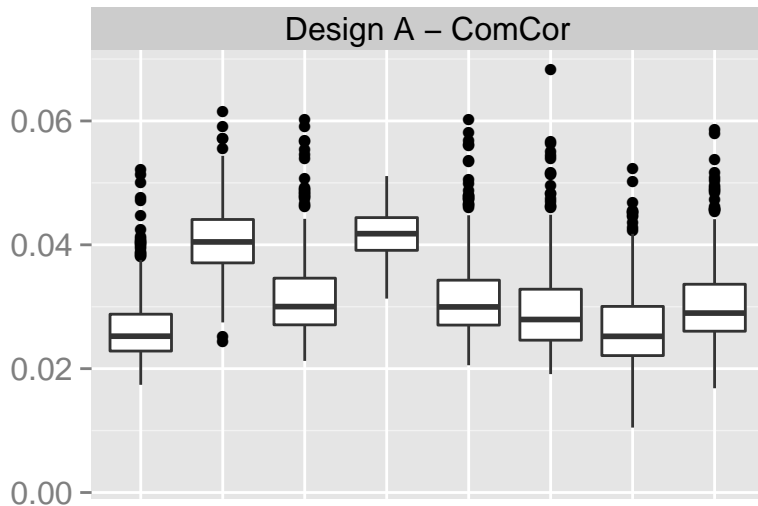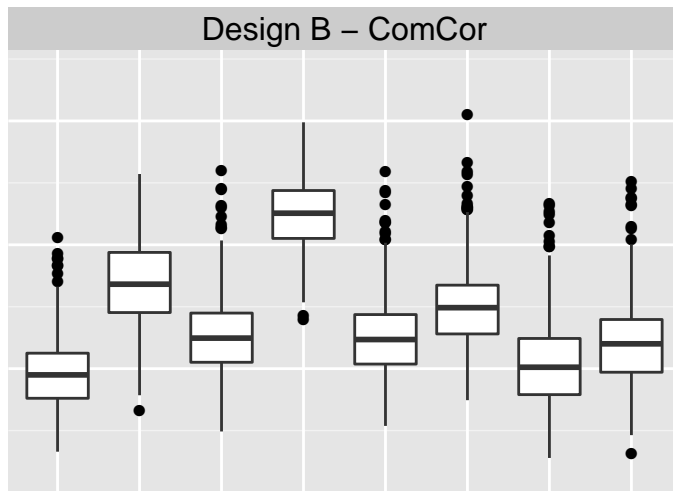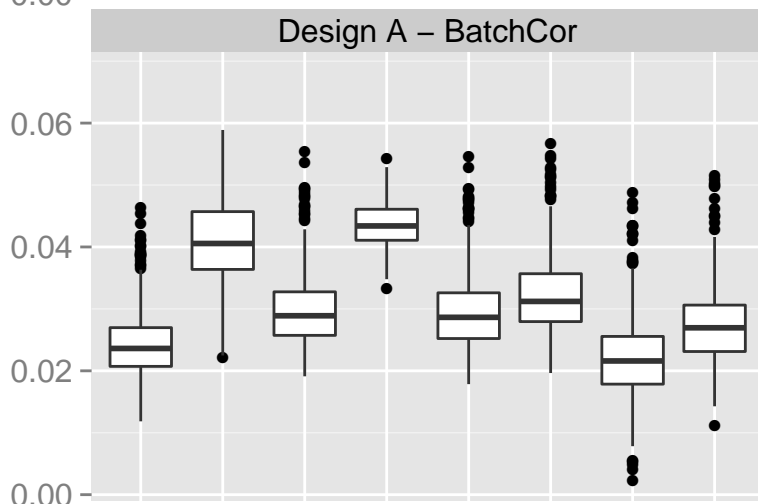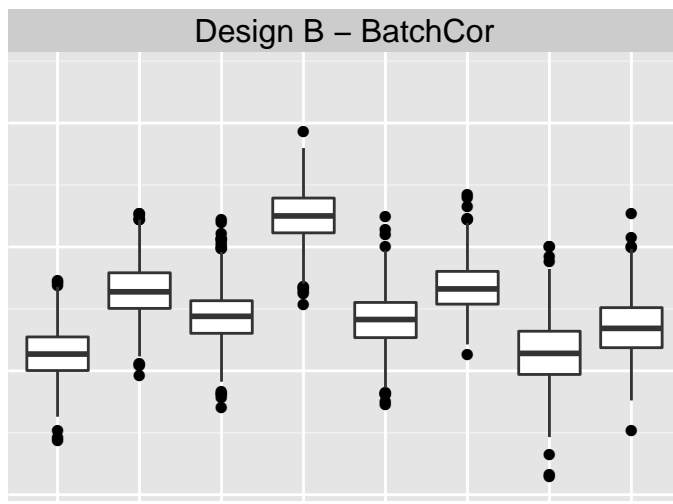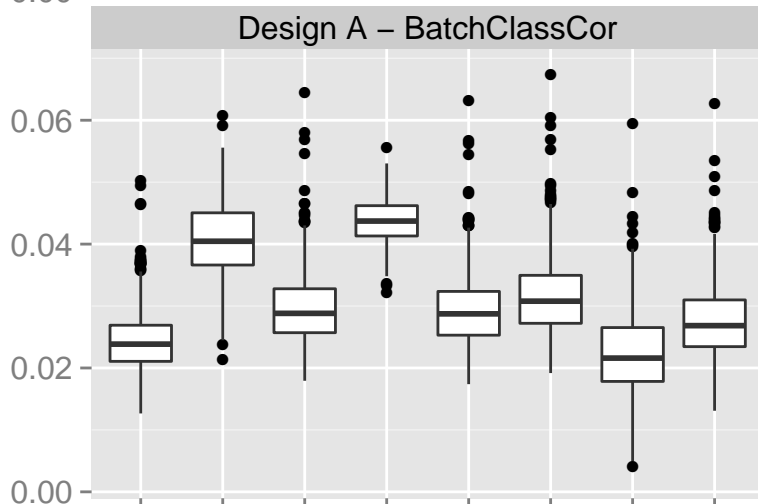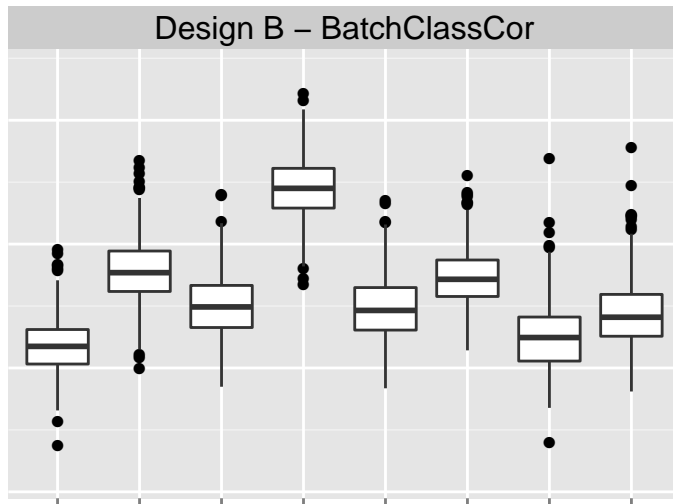

none fabatch combat sva meanc stand ratiog ratioa

Supplement: Additional file 2 — This folder contains all necessary R-Code to reproduce and evaluate the real-data analyses and simulations, as well as Rda-files enabling fast evaluation of the corresponding results. (ZIP 2406 kb) [file 12859_2015_870_MOESM2_ESM.zip › FAbatchPaper/Results/SupplementaryFigure11.pdf]

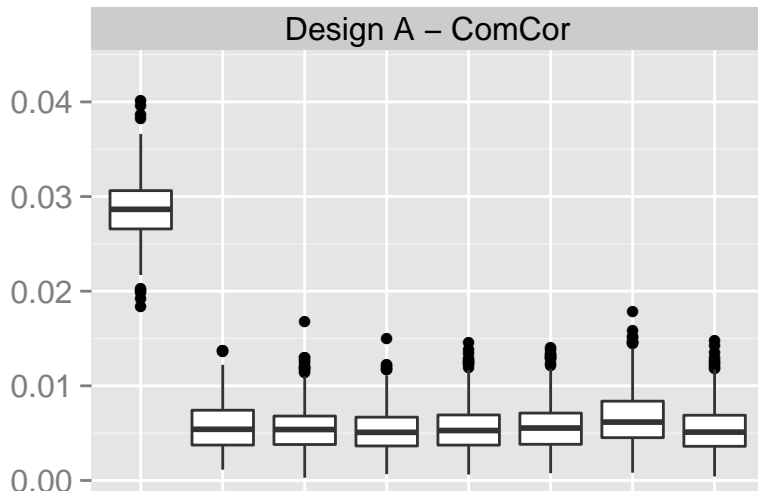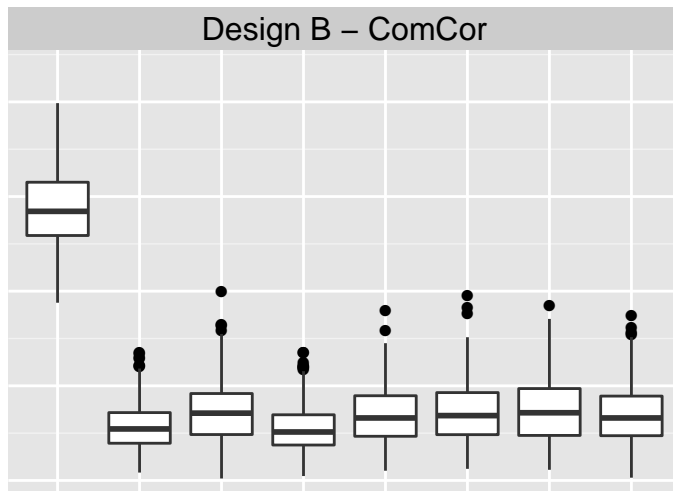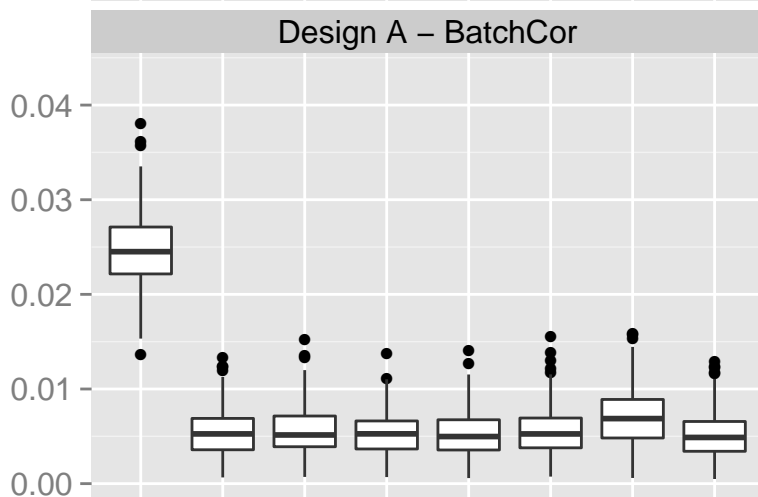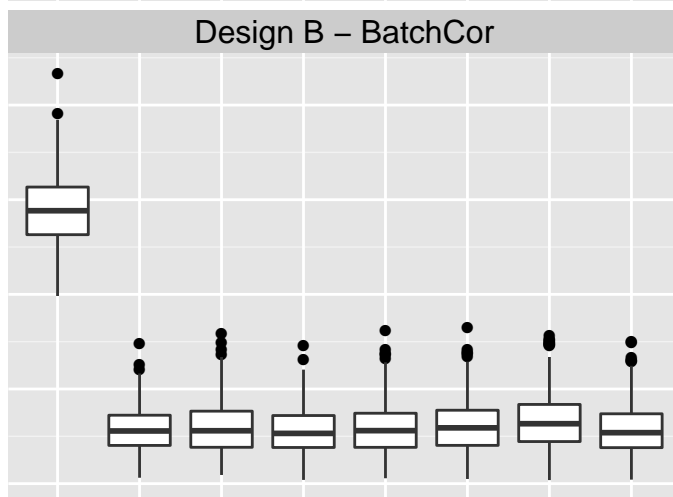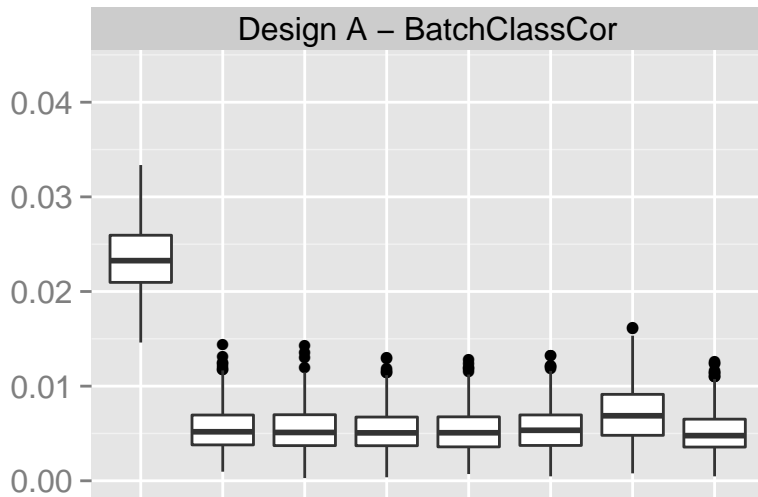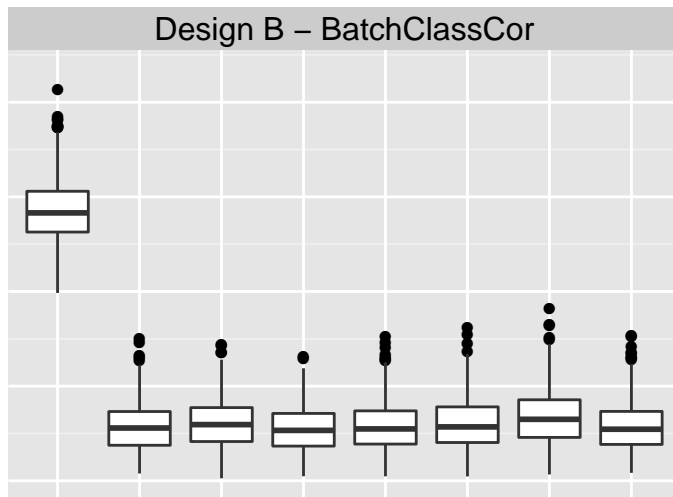

none fabatch combat sva meanc stand ratiog ratioa

Supplement: Additional file 2 — This folder contains all necessary R-Code to reproduce and evaluate the real-data analyses and simulations, as well as Rda-files enabling fast evaluation of the corresponding results. (ZIP 2406 kb) [file 12859_2015_870_MOESM2_ESM.zip › FAbatchPaper/Results/SupplementaryFigure13.pdf]

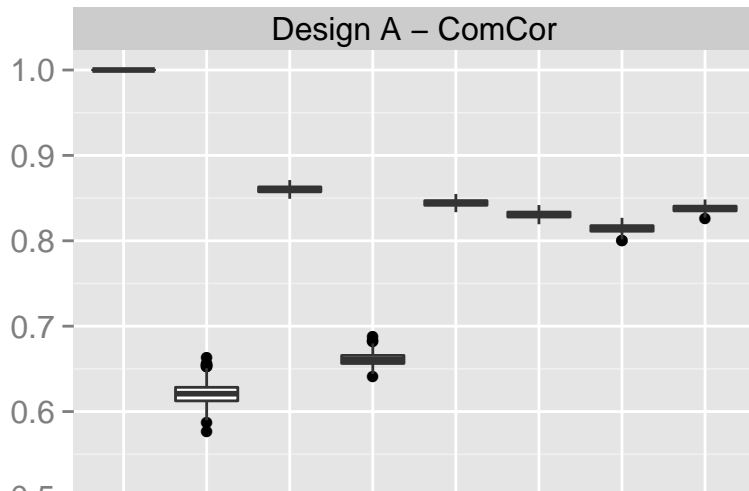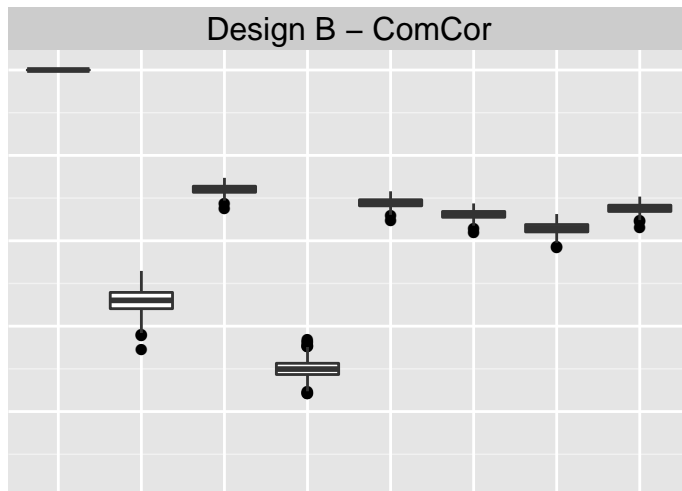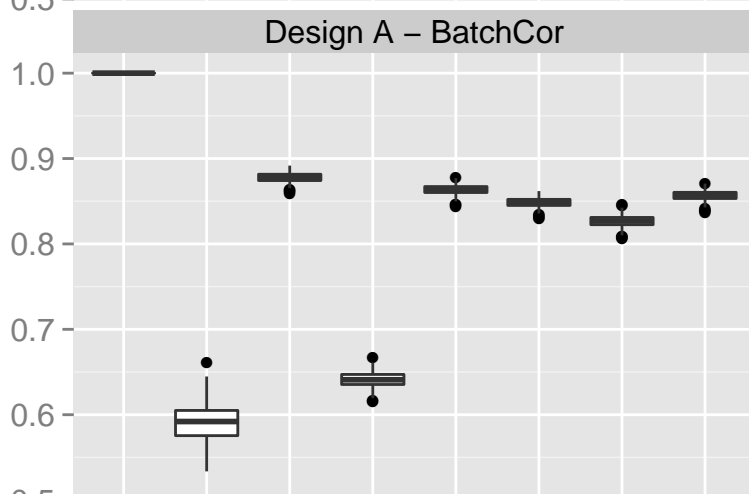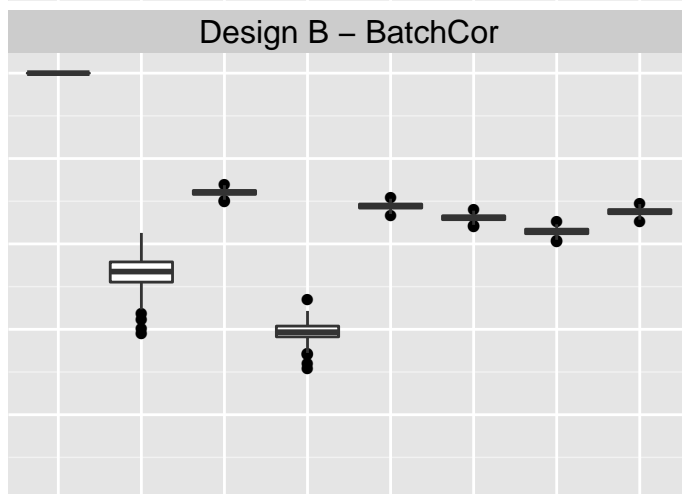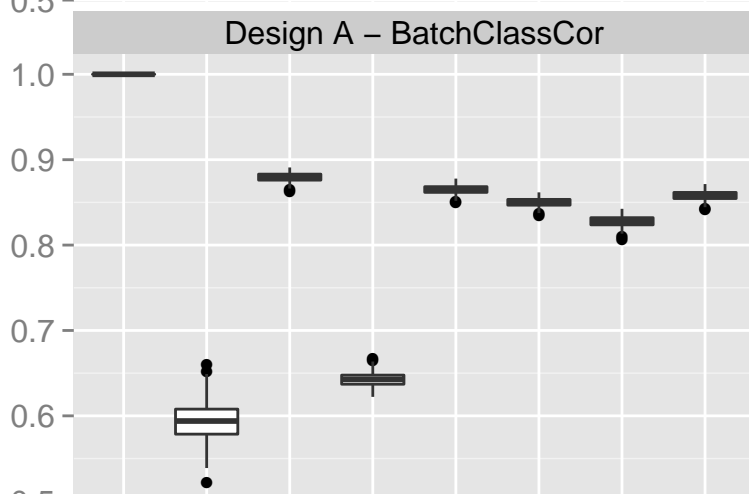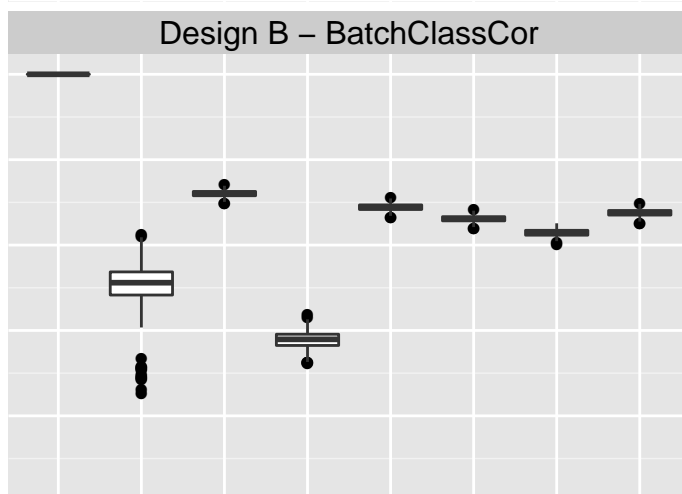

Supplement: Additional file 2 — This folder contains all necessary R-Code to reproduce and evaluate the real-data analyses and simulations, as well as Rda-files enabling fast evaluation of the corresponding results. (ZIP 2406 kb) [file 12859_2015_870_MOESM2_ESM.zip › FAbatchPaper/Results/SupplementaryFigure14.pdf]

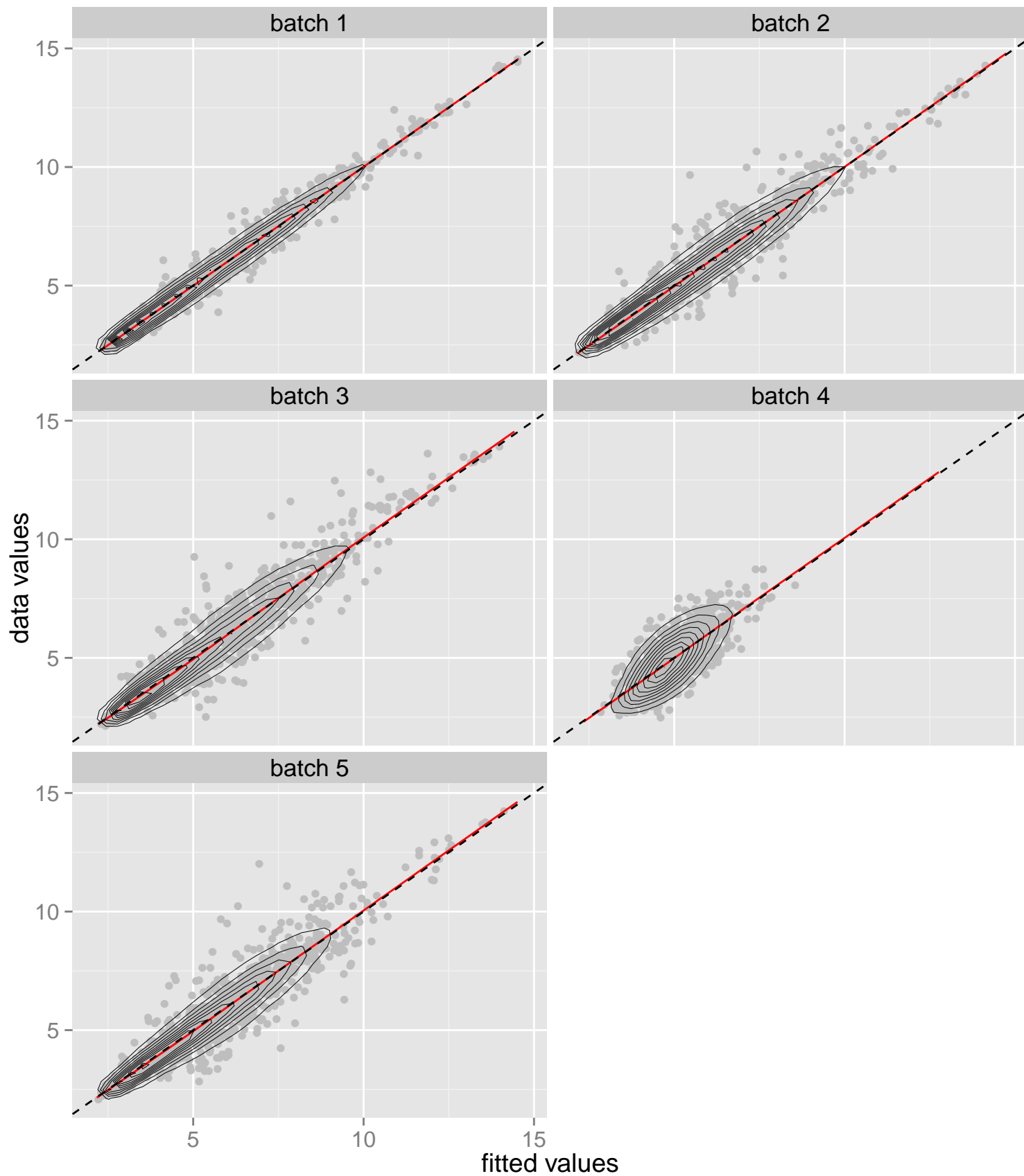

Supplement: Additional file 2 — This folder contains all necessary R-Code to reproduce and evaluate the real-data analyses and simulations, as well as Rda-files enabling fast evaluation of the corresponding results. (ZIP 2406 kb) [file 12859_2015_870_MOESM2_ESM.zip › FAbatchPaper/Results/SupplementaryFigure2.pdf]

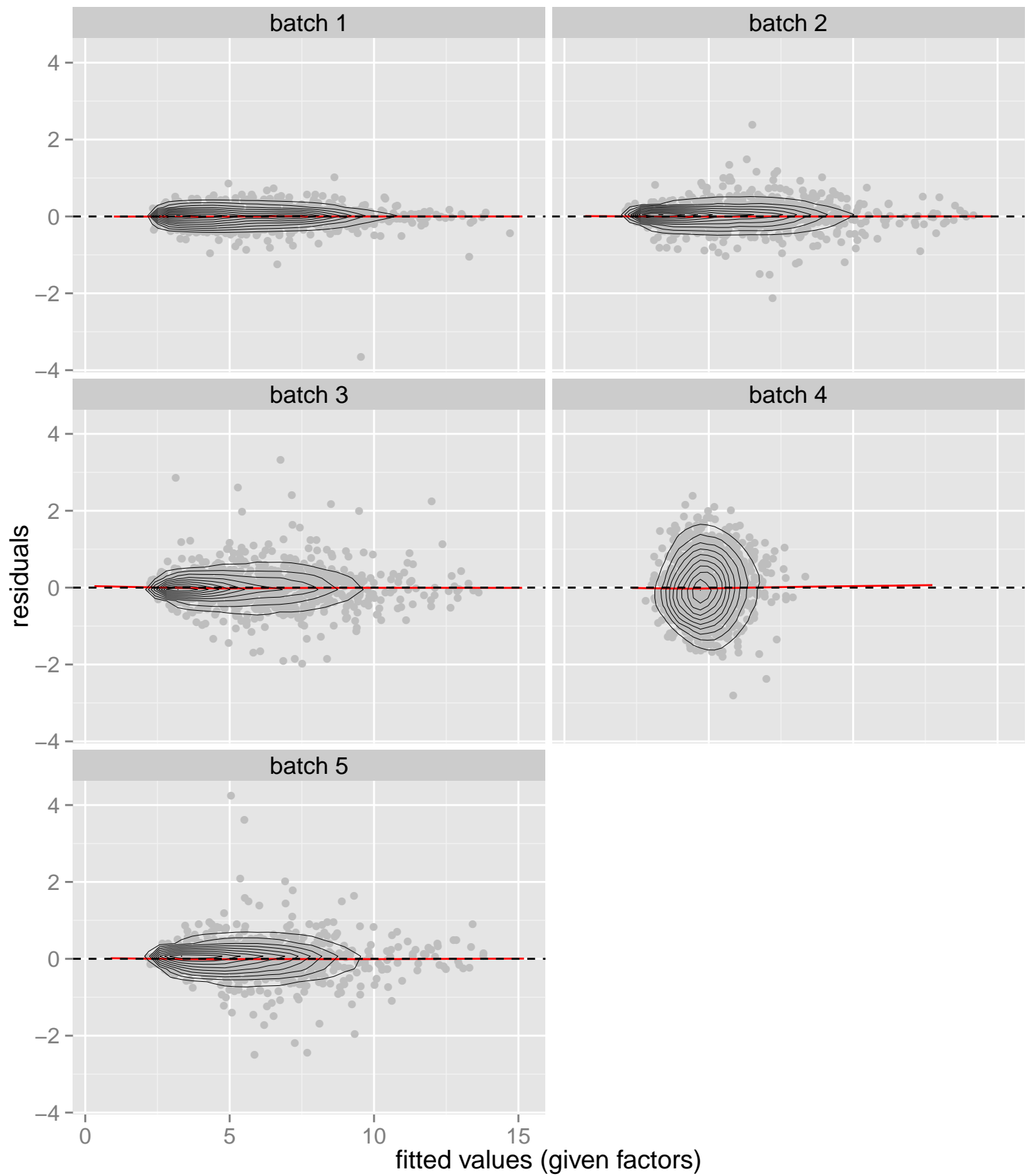

Supplement: Additional file 2 — This folder contains all necessary R-Code to reproduce and evaluate the real-data analyses and simulations, as well as Rda-files enabling fast evaluation of the corresponding results. (ZIP 2406 kb) [file 12859_2015_870_MOESM2_ESM.zip › FAbatchPaper/Results/SupplementaryFigure3.pdf]

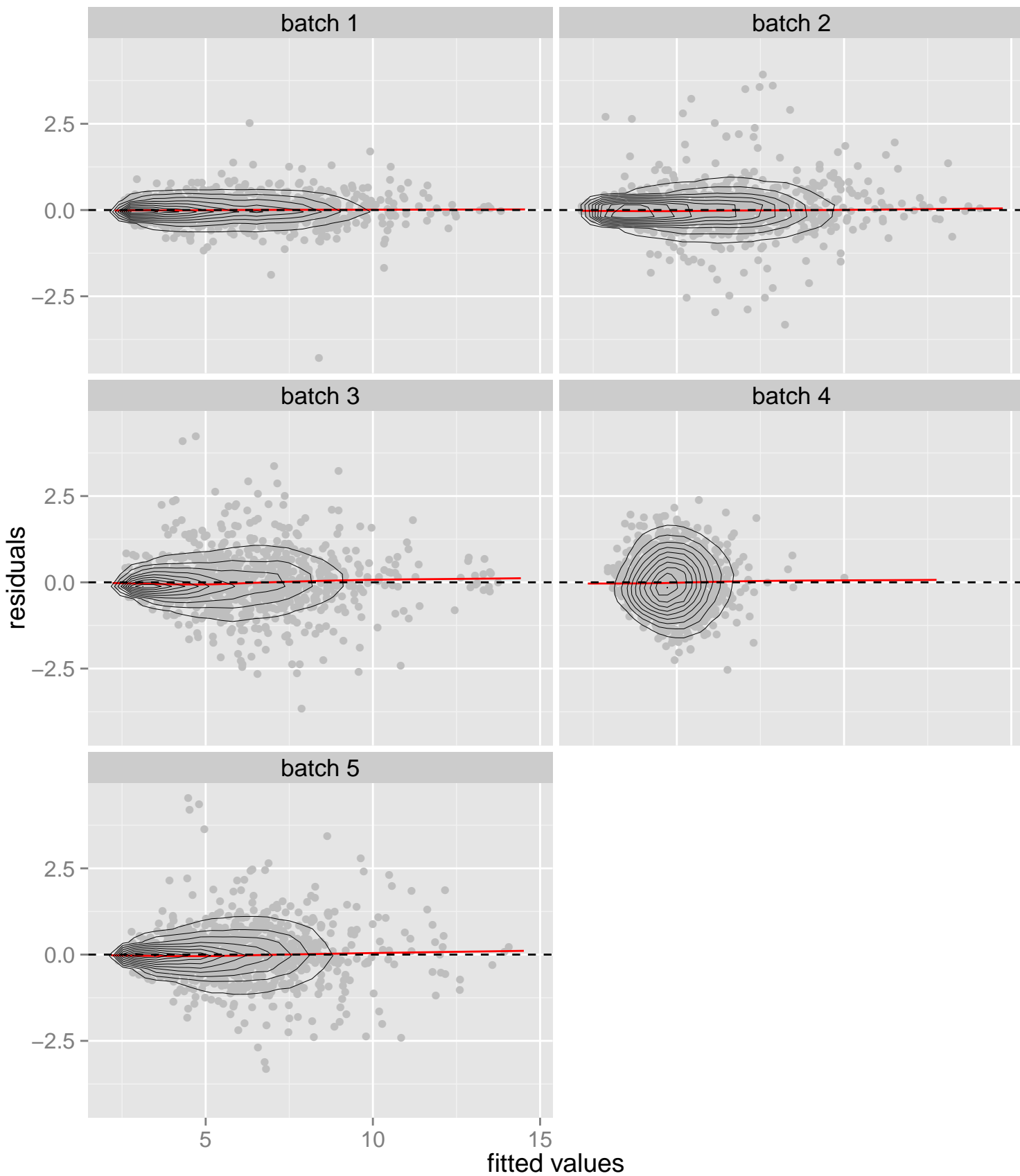

Supplement: Additional file 2 — This folder contains all necessary R-Code to reproduce and evaluate the real-data analyses and simulations, as well as Rda-files enabling fast evaluation of the corresponding results. (ZIP 2406 kb) [file 12859_2015_870_MOESM2_ESM.zip › FAbatchPaper/Results/SupplementaryFigure4.pdf]

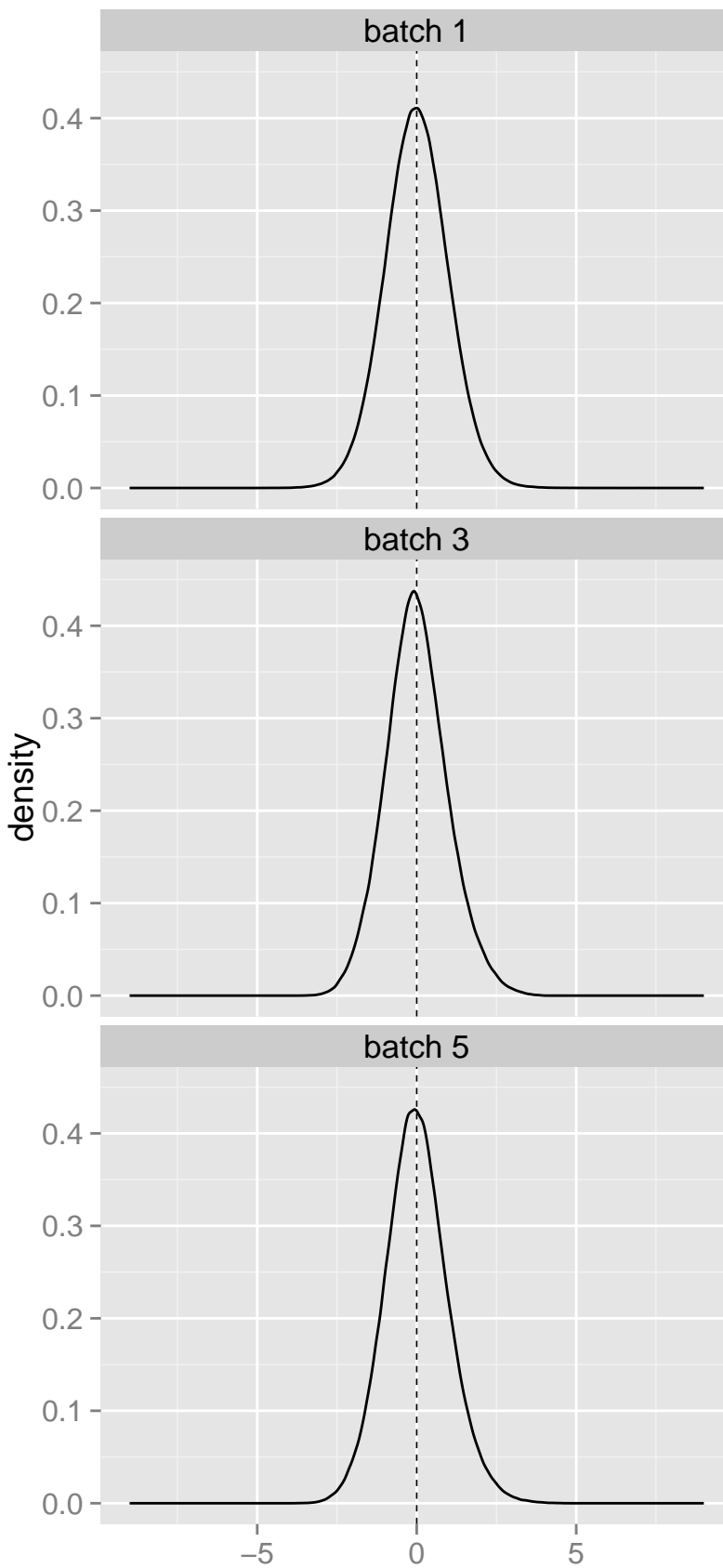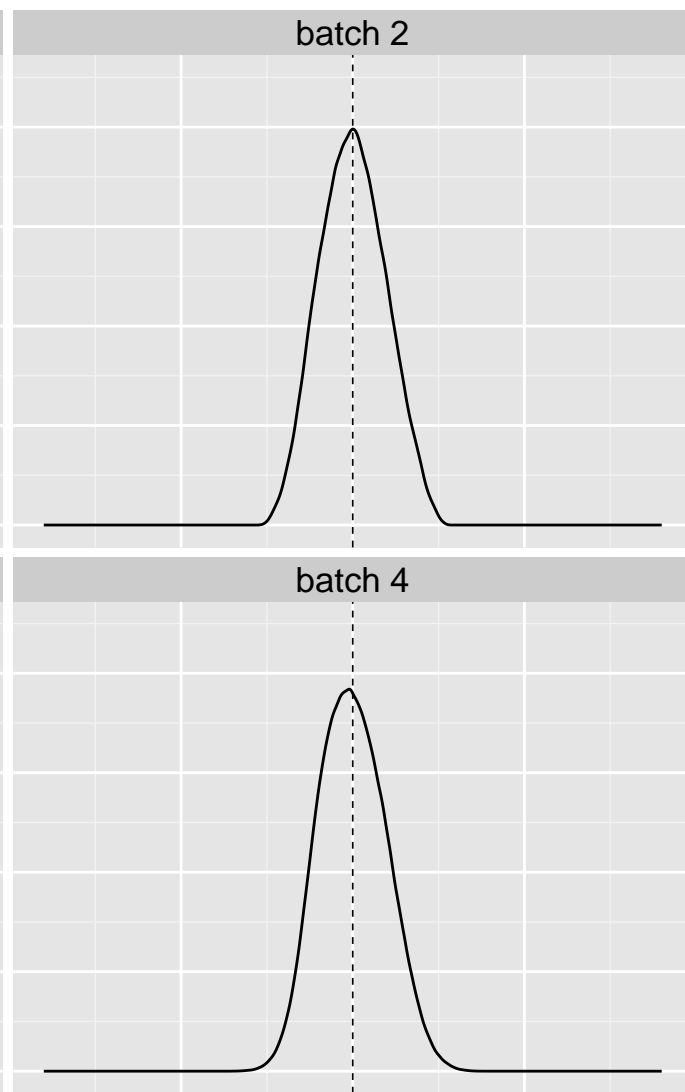

Supplement: Additional file 2 — This folder contains all necessary R-Code to reproduce and evaluate the real-data analyses and simulations, as well as Rda-files enabling fast evaluation of the corresponding results. (ZIP 2406 kb) [file 12859_2015_870_MOESM2_ESM.zip › FAbatchPaper/Results/SupplementaryFigure5.pdf]

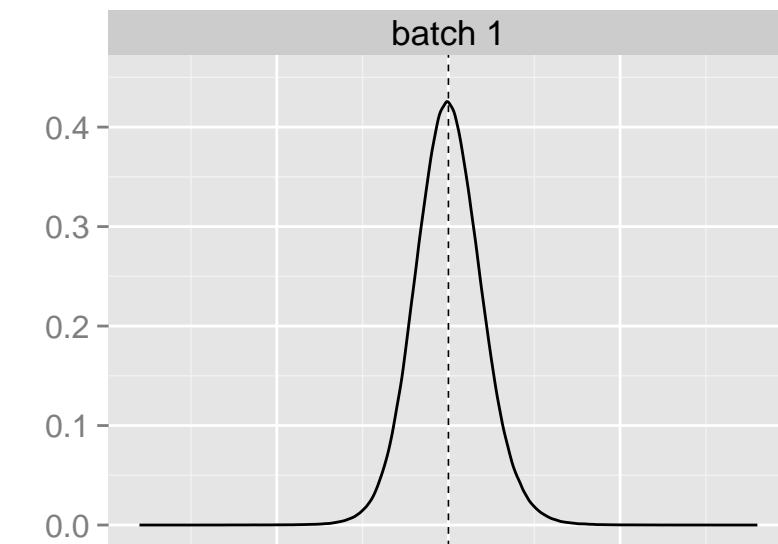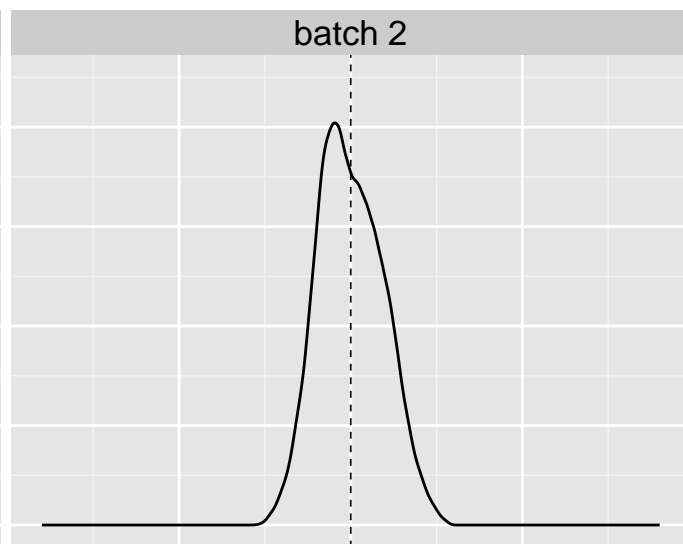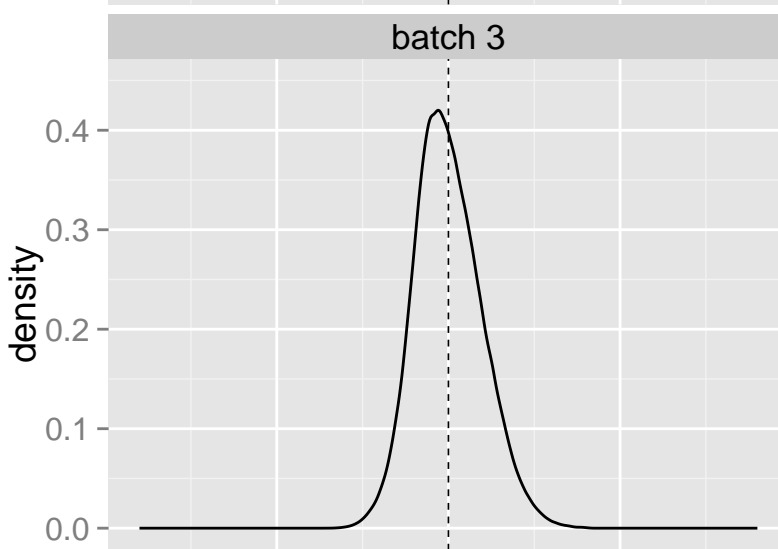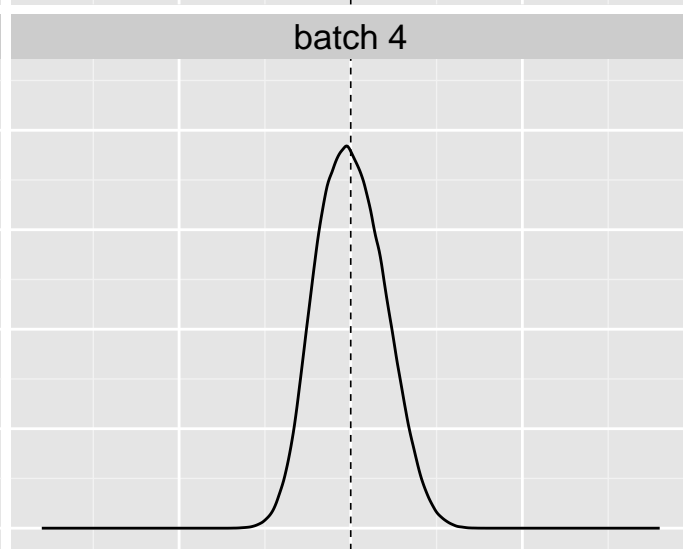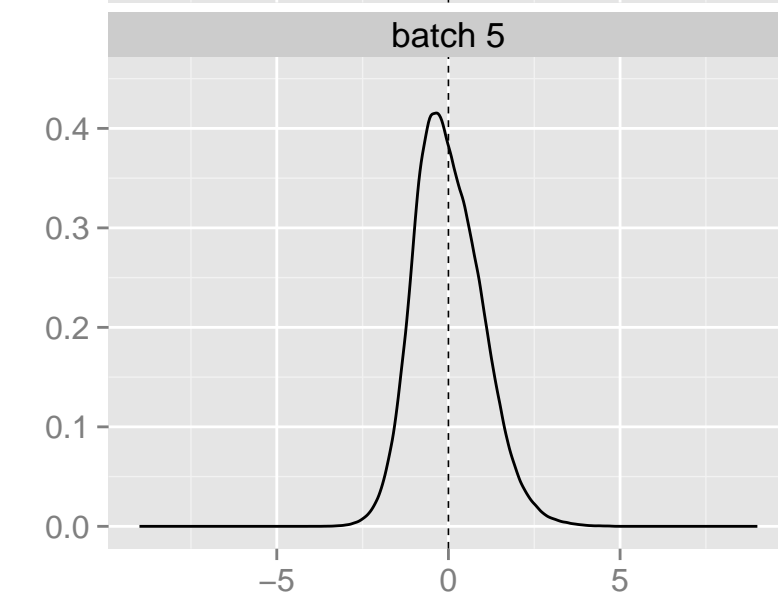

Supplement: Additional file 2 — This folder contains all necessary R-Code to reproduce and evaluate the real-data analyses and simulations, as well as Rda-files enabling fast evaluation of the corresponding results. (ZIP 2406 kb) [file 12859_2015_870_MOESM2_ESM.zip › FAbatchPaper/Results/SupplementaryFigure6.pdf]

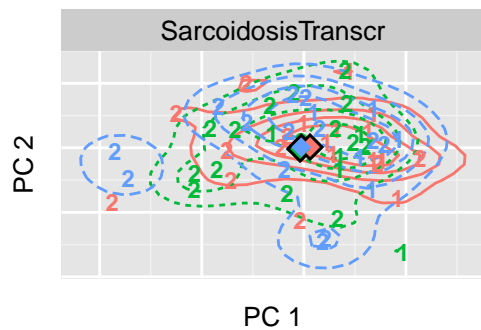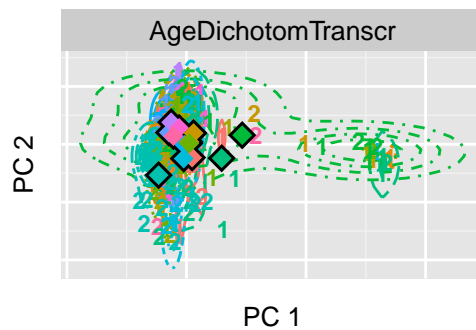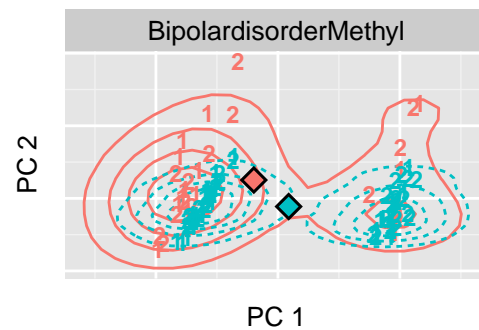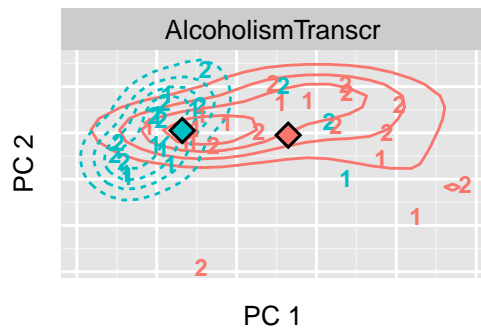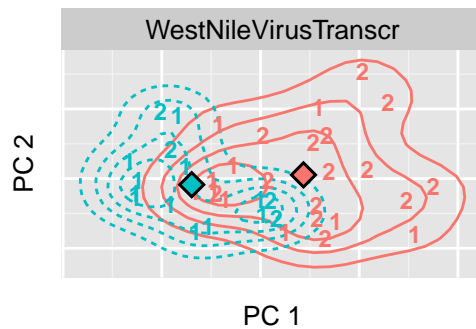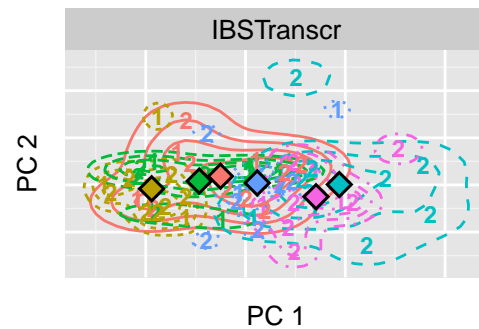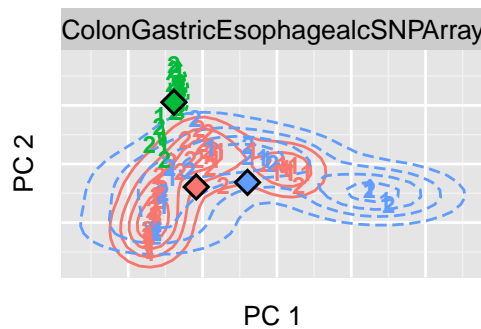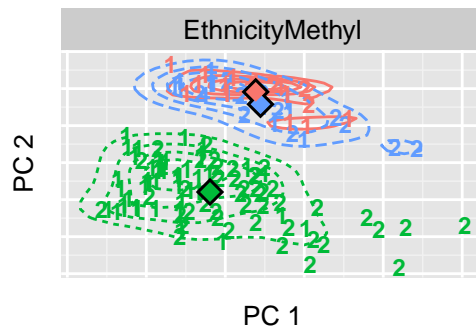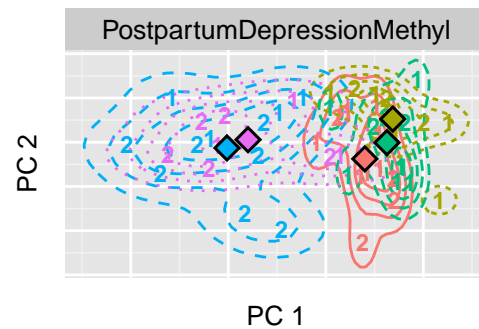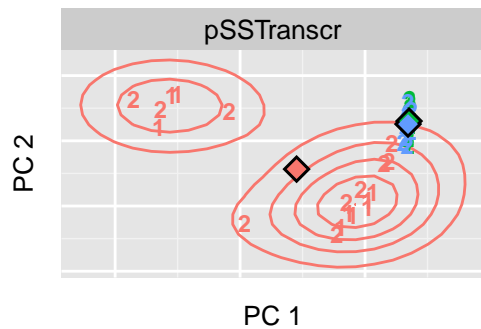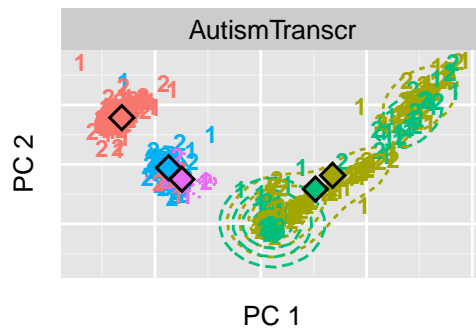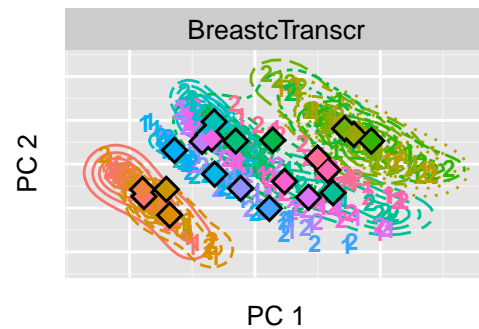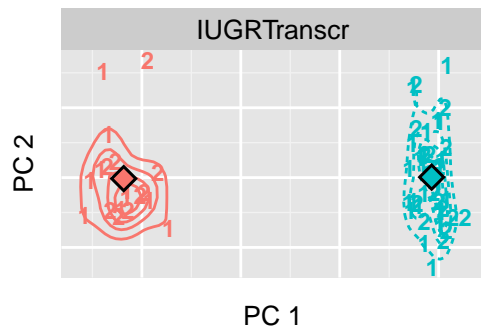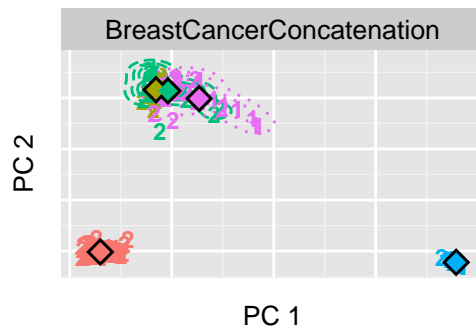

Supplement: Additional file 2 — This folder contains all necessary R-Code to reproduce and evaluate the real-data analyses and simulations, as well as Rda-files enabling fast evaluation of the corresponding results. (ZIP 2406 kb) [file 12859_2015_870_MOESM2_ESM.zip › FAbatchPaper/Results/SupplementaryFigure7.pdf]

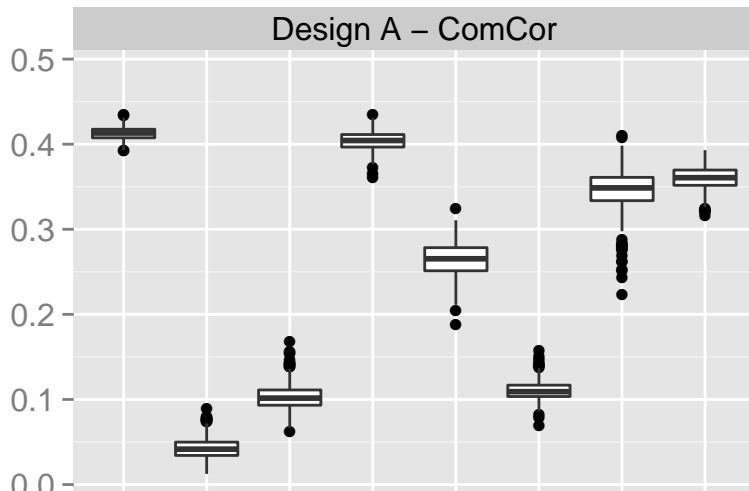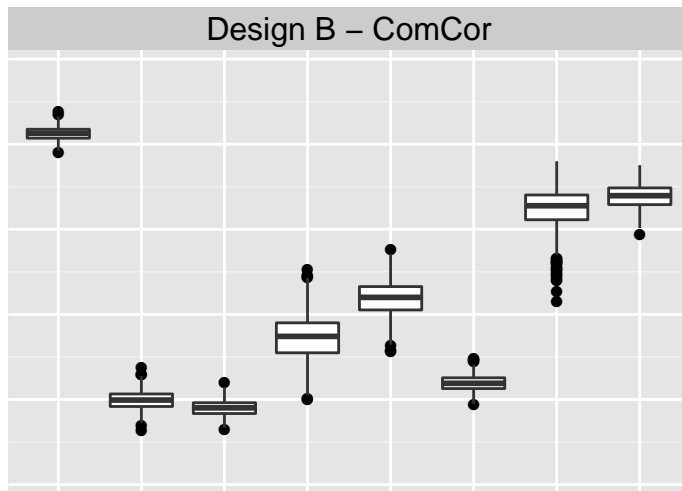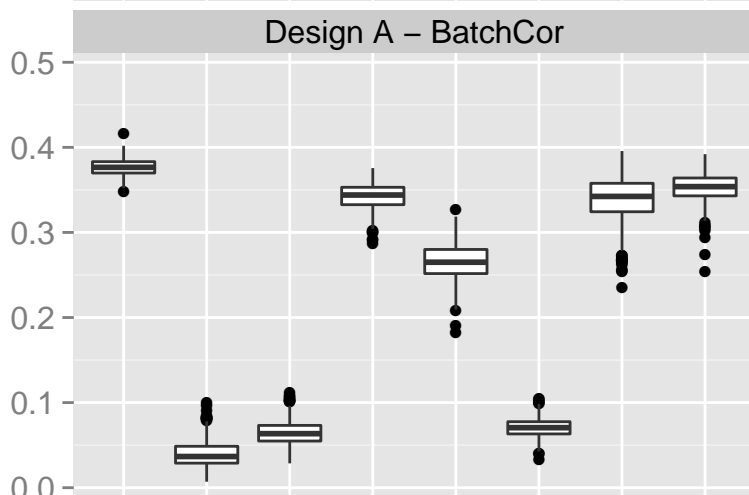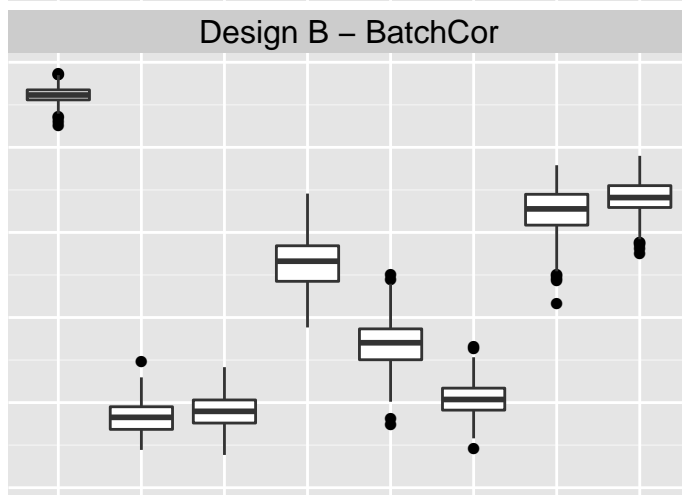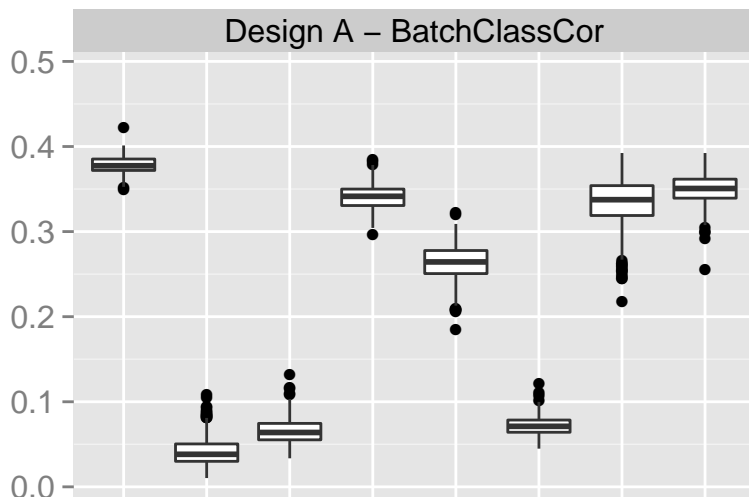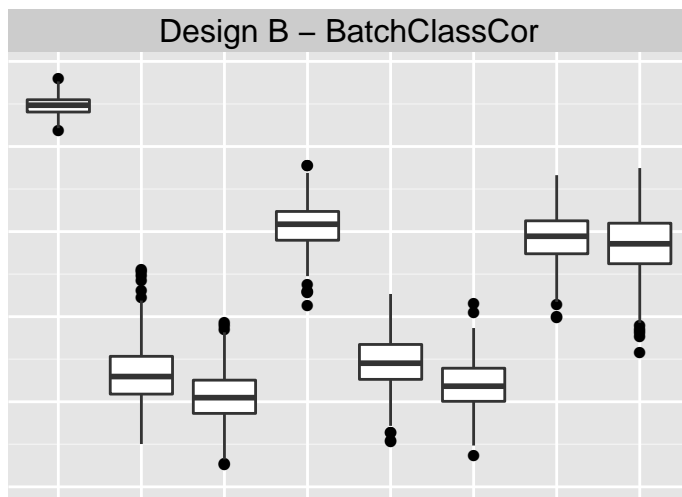

none fabatch combat sva meanc stand ratiog ratioa

Supplement: Additional file 2 — This folder contains all necessary R-Code to reproduce and evaluate the real-data analyses and simulations, as well as Rda-files enabling fast evaluation of the corresponding results. (ZIP 2406 kb) [file 12859_2015_870_MOESM2_ESM.zip › FAbatchPaper/Results/SupplementaryFigure8.pdf]
